# Molecular Tools for Guiding Therapy in Patients With Staphylococcal Bone and Joint Infections: A Diagnostic Test Accuracy Meta-analysis

**DOI:** 10.3389/fendo.2022.792679

**Published:** 2022-07-13

**Authors:** Ke Chen, Yanqiu Wang, Yue Yuan, Wen Qin, Yun-Jian Sheng, Sarfraz Ahmed, Changfeng Sun, Cun-Liang Deng, Suvash Chandra Ojha

**Affiliations:** ^1^ Department of Infectious Diseases, The Affiliated Hospital of Southwest Medical University, Luzhou, China; ^2^ Southwest Medical University, Luzhou, China; ^3^ Department of Basic Sciences, University of Veterinary and Animal Sciences Lahore, Narowal, Pakistan

**Keywords:** bone and joint infection, NAAT accuracy, anti-staphylococcal therapy, systematic review, meta-analysis

## Abstract

**Background:**

Timely detection of causative pathogens and their antimicrobial resistance are essential for guiding targeted therapies in bone and joint infections (BJI) patients. We performed a systematic review and meta-analysis to assess the diagnostic value of testing osteoarticular samples with the nucleic acid amplification tests (NAAT) for effective staphylococcal strain identification and the administration of appropriately targeted antimicrobial agents in BJI patients.

**Methods:**

Five databases, including PubMed, Embase, Scopus, Web of Science, and the Cochrane Library, were searched for related publications from inception to July 24, 2021. Studies comparing the diagnostic accuracy of NAAT to a microbiological culture reference standard of osteoarticular specimens were eligible. Pooled summary values of sensitivity, specificity, positive likelihood ratio (PLR), negative likelihood ratio (NLR), and diagnostic odds ratio (DOR) of NAAT compared to the microbiological culture reference standard were calculated using bivariate random-effects meta-analyses.

**Results:**

From 906 citations, 11 studies were included. Eleven studies comprising 13 datasets (*n* = 1047) evaluated NAAT accuracy for methicillin-sensitive *Staphylococcus aureus* (MSSA) identification, while seven studies comprising nine datasets (*n* = 727) evaluated methicillin-resistant *Staphylococcus aureus* (MRSA) identification. Against the microbiological culture reference standard, the pooled summary estimates for detection of both MSSA [sensitivity: 0.89 (95% confidence interval [CI] 0.84–0.93), specificity: 0.99 (95% CI 0.97–0.99), PLR: 34.13 (95% CI 20.54–56.73), NLR: 0.19 (95% CI 0.12–0.3), and DOR: 283.37 (95% CI 129.49–620.1)] and MRSA [sensitivity: 0.81 (95% CI 0.67–0.91), specificity: 1.0 (95% CI 0.99–1.0), PLR: 62.1 (95% CI 24.5–157.6), NLR: 0.33 (95% CI 0.16–0.69), and DOR: 300.25 (95% CI 85.01–1060.5)] were comparable. Heterogeneity was moderate. GeneXpert was frequently used among NAA tests, and its diagnostic accuracy was in line with the overall pooled summary estimates. The heterogeneity in diagnostic efficacy (*P >*0.05) could not be explained by a meta-regression and subgroup analysis of the research design, sample condition, and patient selection technique.

**Conclusions:**

Our study suggested that NAAT can be applied as the preferred prescreening test for the timely diagnosis of staphylococcal strains associated with BJI in osteoarticular samples for successful antimicrobial therapy.

## Introduction

Bone and joint infections (BJI) are linked to extraordinarily high rates of morbidity and mortality, and *Staphylococcus aureus* is the most frequent pathogen in virtually all forms of BJI (1, 2). Several studies published in the previous decade found that the relative frequencies of methicillin-resistant *S. aureus* (MRSA) increased faster than that of methicillin-susceptible *S. aureus* (MSSA), and that MRSA was a major contributor to the difficult-to-treat BJI (3–5). Both children and adults with a weakened immune system are susceptible to illness (6). Pathogenic strains enter the circulation and infiltrate numerous organs through open wounds as a result of being in an immunosuppressive environment, eventually infecting the bones (osteomyelitis), joints (septic arthritis), or developing a biofilm on a prosthesis (septic arthritis) (7–9). The surface of prosthetic implants for knees, hips, shoulders, ankles, or elbows serves as a reservoir site for *S. aureus*, where they are known to develop a biofilm and colonize to encourage the establishment of highly resistant strains that are very difficult to remove with traditional antibiotics. Despite improvements in the knowledge and treatment of bone and joint infections (BJI) (1, 6), these infections continue to represent a diagnostic challenge to clinicians and often leave patients disabled owing to high recurrence rates.

Treatment delays may have catastrophic consequences for patients with BJI. Therefore, intervention with broad-spectrum glycopeptide antibiotics active against staphylococci is typically initiated in the community as an empirical antimicrobial therapy while conventional culture results are awaited (10). Clindamycin monotherapy is also used successfully in certain instances, either in conjunction with or after initial broad-spectrum antibiotic treatment (11). Alternatively, antibiotics such as linezolid, daptomycin, and quinolones have all been proven to be effective for MRSA-associated BJI (6, 12–14). However, choosing empirical antistaphylococcal treatment is challenging since, in addition to staphylococcal strains that cause BJI, other pathogens such as Gram-negative bacilli, streptococci, enterococci, and anaerobes are less often identified (15). Once microbe identification and susceptibility are confirmed, therapy may be adjusted to isolated microorganisms, including discontinuation of glycopeptides when MSSA is identified. There are risks associated with this approach, such as patients receiving an excessive number of broad-spectrum antibiotics, which can alter the patient’s microflora, expose them to drug-induced toxicity, and increase the number of drug-resistant bacteria (16). Vancomycin, the most often prescribed antibiotic for MRSA infections, is less efficient than oxacillin in treating MSSA infections (17). If the first antibiotics are severely insufficient and are changed once the diagnostic tests are accessible, the death rate does not improve substantially. Therefore, balancing these two conflicting objectives, notably the requirement for comprehensive coverage while avoiding needless medicines, is becoming more essential.

Medical intervention of BJI has mainly depended on direct Gram stain and regular identification of pathogenic organisms through conventional culture-based methods to guide treatment. However, BJI diagnosis remains challenging owing to Gram stain’s poor sensitivity (18, 19), and a microbiological diagnosis cannot be established in up to 50% of BJI patients using conventional microbiological culture methods (6). Poor microbial culture detection rates can be attributed to a combination of prior antimicrobial therapy prior to obtaining specimens, low microbial concentration in osteoarticular fluid samples, and possibly causal agents that are difficult to isolate in the laboratory due to stringent requirements (20, 21). Furthermore, the conventional culture-based method, which comprises growth-based assays, colony morphology, and microdilution resistance testing, is time-consuming and labor-intensive. Even with a positive microbial culture, it takes 48-72 hours for staphylococcal culture and antibiotic susceptibility tests to identify the causal organism. The high proportion of culture-negative episodes complicates patient care and antimicrobial selection, resulting in patients missing out on the best treatment options. Therefore, in patients with BJI, clinical suspicion of staphylococcal infection is important for enabling diagnostic and therapeutic action.

Previously, a few studies showed that NAAT was accurate in the diagnosis of staphylococcal pneumonia and pediatric sepsis (22, 23); however, to the best of our knowledge, there is no published information on the functioning of the NAAT test for the identification of pathogens in BJI. NAAT, which usually have a faster reaction time and are unaffected by antibiotic exposure, may assist in the development of an etiological diagnosis to help guide patient treatment. Furthermore, NAAT detection of the *mecA* gene is widely regarded as the gold standard for MRSA identification, which could be useful in directing therapy and preventing needless inpatient care. The 16S rDNA polymerase chain reaction (PCR) methods may be considered; however, in most cases, the requirement to utilize a sequencing step of the amplified product is costly and time-consuming (24). NAAT has been studied extensively in recent years for the diagnosis of staphylococcal BJI, including conventional PCR (25), real-time PCR (26), multiplex PCR (27, 28), multiplex PCR-UITI (29–31), and GeneXpert (32–35); however, evidence is scarce on the relevance of these tests in staphylococcal BJI treatment. Given the significance of decision support in patients with staphylococcal BJI, we performed a systematic review and analyzed the available data to show the diagnostic performance of NAAT vs microbiological culture.

## Methods

### Search Strategy

Preferred Reporting Items for Systematic Reviews and Meta-Analyses (PRISMA) guidelines were followed for diagnostic test accuracy (36). PubMed, Embase, Web of Science, Scopus, and the Cochrane Library were systematically searched through electronic databases from the establishment of the library until July 24, 2021. The search strategy was developed based on key terms used in literature for BJI, which includes: (‘*Staphylococcus aureus’* OR ‘*S. aureus*’ OR ‘Methicillin-resistant *Staphylococcus aureus*’ OR ‘MRSA’) AND (‘Osteomyelitis’ OR ‘Arthritis’ OR ‘Bone’ OR ‘Joint’ OR ‘Musculoskeletal infection’ OR ‘Bone and joint infection’ OR ‘Osteoarticular infection’ OR ‘Discitis’ OR ‘Orthopedic’ OR ‘BJI’ OR ‘Synovial fluid’ OR ‘Joint effusion’ OR ‘Bone sample’) AND (‘Nucleic acid amplification’ OR ‘NAAT’ OR ‘Molecular assay’ OR ‘Loop-mediated isothermal amplification’ OR ‘LAMP’ OR ‘Polymerase chain reaction’ OR ‘PCR’ OR ‘Ligase chain reaction’ OR ‘LCR’ OR ‘Real-time PCR’ OR ‘qPCR’ OR ‘RT-PCR’ OR ‘Xpert’ OR ‘GeneXpert’ OR ‘Amplicor’ OR ‘SeptiFast’ OR ‘ProbeTec’ OR ‘Roche’ OR ‘Gen-Probe’ OR ‘FilmArray’ OR ‘Cepheid’ OR ‘Abbott’ OR ‘hyplex StaphyloResist’ OR ‘GeneOhm’ OR ‘LightCycler’) AND (‘Sensitivity’ OR ‘Specificity’ OR ‘Accuracy’). Furthermore, citations of reviews and included publications were also searched.

### Study Selection

The studies generated by the search results were imported into the EndNote X9 citation manager (Thomson Reuters, New York, NY, USA), and duplicates were manually removed to ensure no overlapping studies. Two authors (K. Chen and Y. Wang) independently screened citations by title and abstract per predefined eligibility criteria, and irrelevant studies were removed. All studies that met the standard BJI definition, including persistent fever, osteomyelitis, septic arthritis, diabetic foot, pyomyositis, discitis, and deep vein thrombosis, were included. The full-text review for all eligible studies was carried out and analyzed for diagnostic accuracy data. The data from two separate researchers were compared, and any disparities were sorted by mutual agreement.

### Inclusion and Exclusion Criteria

Inclusion criteria comprised: (i) patients suspected of having septic arthritis or osteomyelitis, in whom staphylococcal strains were cultivated from synovial fluid, blood, or bone biopsies; (ii) in instances of clinically or radiographically identified BJI complicated by abscess development, a culture from an abscess or orthopedic implant was suitable; (iii) NAAT accuracy as an index test in osteoarticular specimens; (iv) detect staphylococcal strains and methicillin resistance using microbiological culture as the gold standard; and (v) inclusion of specificity, sensitivity, or adequate information to construct 2×2 contingency tables.

Exclusion criteria involved reviews, letters to the editor, meta-analyses, editorials, conference proceedings and abstracts, case reports, animal experiments, commentaries, and mechanism studies, as were studies with fewer than ten participants. NAAT results other than diagnostic accuracy and studies using 16S rRNA PCR followed by sequencing of the generated product were not eligible. Studies with non-interpretable test findings and those that failed to identify staphylococcal strains in suspected patients using both the index test and the microbiological reference standard were eliminated.

### Data Extraction

Two reviewers (K. Chen and Y. Wang) piloted the data extraction form, with critical feedback from a third (S.C. Ojha). Two investigators (K. Chen and Y. Wang) independently extracted results from all selected studies using a predefined strategy. Following data extraction, findings were compared, and discussions were resolved until a consensus was achieved. The authors of published studies were contacted where accuracy data or sample preparation procedures were ambiguous. We generated two-by-two contingency tables for NAAT performance vs. the microbiological culture reference standard using data from the articles.

### Quality Assessment

The Quality Assessment of Diagnostic Accuracy Studies-2 (QUADAS-2) tool was used to assess each included study’s risk of bias (37). The methodological quality was assessed independently by two investigating reviewers. Acceptable microbiological reference standards were microbiological culture against NAAT, performed on specimens conventionally used to diagnose staphylococcal BJI (osteoarticular samples, synovial fluid, blood, bone tissue, and abscess). NAAT was not included in the reference standard. The risk of bias was assessed in four QUADAS-2 domains (patient selection, index test, reference standard, and flow and timing), and three domains (patient selection, index test, and reference standard) were evaluated for applicability. The spectrum and selection biases of participants were determined. Each domain was evaluated for bias risk using signalling questions that can be answered with “yes,” “no,” or “unclear,” and are categorized as “low,” “high,” or “unclear.” A third reviewer (S.C. Ojha) was consulted in the event of an unresolved disagreement.

### Statistical Analysis

Data obtained from two-by-two contingency tables were utilized to compute pooled sensitivity and specificity and the related 95% confidence intervals (CIs). In two-by-two contingency tables, missing values were replaced with 0.5 to attain a zero correction. RevMan (version 5.4; Nordic Cochrane Centre, Copenhagen, Denmark) was used to assess the methodological quality of included studies and generate summary plots (38). On forest plots, results from individual research and accuracy estimates are displayed. Meta-DiSc 1.4 (Cochrane Colloquium, Barcelona, Spain) was used to generate pooled summary estimates of specificity, sensitivity, diagnostic odds ratio (DOR), likelihood ratios, and data heterogeneity using bivariate random-effect hierarchical models (39). To assess between-study heterogeneity, we used the *I*-square (*I* ^2^) statistics (40). Different sample conditions (fresh/frozen), study design (prospective/other), and country status (developing/developed) were analyzed as possible sources of heterogeneity using subgroup analysis. To determine publication bias, Deek’s funnel plot asymmetry test was used (41). Generally, a *P*-value of <0.05 was considered statistically significant.

## Results

### Literature Selection

**Figure 1:**
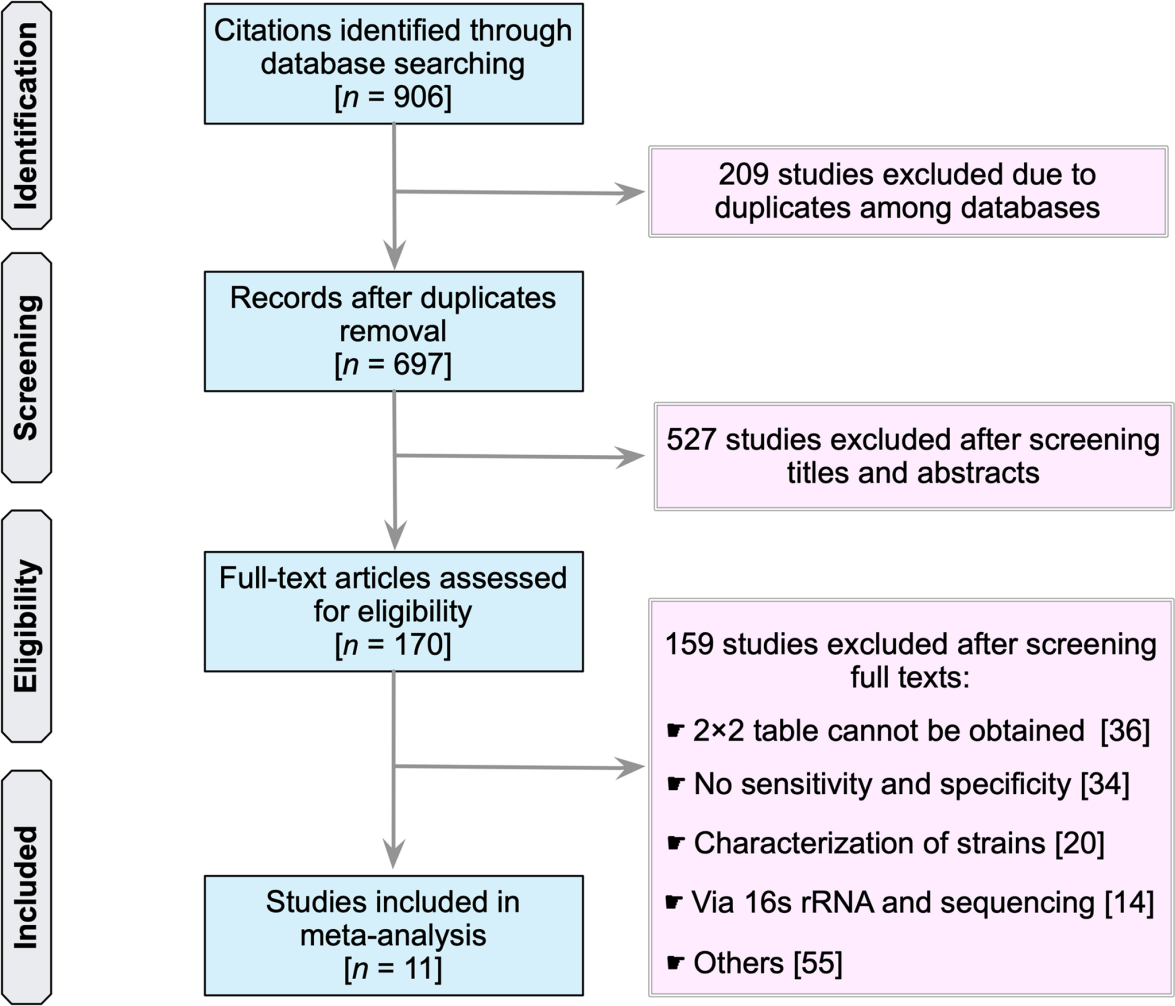
Flow chart of study selection.

Our search identified a total of 906 studies (PubMed, 405; Embase, 37; Scopus, 344; Web of Science, 115; and the Cochrane Library, 5) (**Figure 1**). The first step was to remove 209 duplicate articles manually. Next, 697 studies were screened for relevancy based on their titles and abstracts. Subsequently, 170 studies that were deemed potentially relevant were subjected to a full-text review. References of potentially relevant articles were screened for their relevancy. **Table S1** summarizes the reviewed studies and the reasons why these studies were excluded (*see* **Supplementary Table 1** **)**. Finally, 11 publications fulfilled the inclusion criteria and were used in subsequent analyses (25–35).

### Characteristics of the Included Studies

**Table 1** summarizes the characteristics of 11 eligible studies. Ten studies were conducted in high-income countries (25, 26, 28–35), while one study was conducted in middle-income countries (27). Eleven studies included 13 datasets. All studies comprising 13 datasets (*n* = 1047) assessed the accuracy of NAAT for MSSA detection, while seven studies containing nine datasets (*n* = 727) assessed the accuracy of NAAT for MRSA detection (25–27, 32–35). With a median value of 72, the total number of diagnostic samples received varied from 19 to 182. All experimental procedures were performed in tertiary care hospitals or a reference laboratory. Only studies that were published in English before July 24, 2021, were considered.

**Table 1:** Baseline features of included studies.

Author [Ref.]	Year	Location	Setting	Prosenroll	Patients selection	Specimen type	Specimencondition	Total Sample	NAAT specifics	Reported feature
Bonilla [25]	2011	USA	TCC	No	Convenience	Synovial fluid	Fresh/Frozen	63	PCR, LC PCR, TaqMan PCR	Inflammmatory arthritis
Dubouix-Bourandy [32]	2011	France	TCC	Yes	Convenience	Synovial fluid, tissue	Fresh	135	Xpert	Osteoarticular infection
Gan [27]	2020	China	TCC	No	Convenience	Osteoarticular samples	Fresh/Frozen	41	mPCR	Osteoarticular infection
Kim [28]	2010	Korea	RL	No	Convenience	Synovial fluid	Fresh/Frozen	80	mPCR	Septic arthritis
Morgenstern [29]	2018	Germany	TCC	Yes	Consecutive	Synovial fluid	Fresh	142	Unyvero-ITI	PJI
Saeed [26]	2010	UK	RL	No	Convenience	Tissue	Fresh	19	RT-PCR	BJI
Sambri [33]	2017	Italy	RL	Yes	Convenience	Prosthesis/implant	Fresh	70	Xpert	PJI
Searns [34]	2019	USA	TCC	No	Convenience	Bone, synovial fluid	Fresh/Frozen	182	Xpert	Musculoskeletal infections
Sigmund [30]	2019	Austria	TCC	Yes	Consecutive	Synovial fluid	Fresh	72	Unyvero-ITI	Septic arthritis
Suren [31]	2020	Germany	RL	Yes	Convenience	Synovial fluid	Frozen	26	Unyvero-ITI	PJI
Valour [35]	2014	France	TCC	No	Convenience	Osteoarticular sample	Frozen	91	GeneXpert	BJI

BJI, bone and joint infections; mPCR, multiplex PCR; LC PCR, LightCycler PCR; PJI, prosthetic joint infection; Pros enroll, prospective enrollment; RL, reference laboratory; RT-PCR, real-time PCR; TCC, tertiary care center.

### Quality Appraisal

**Figure 2:**
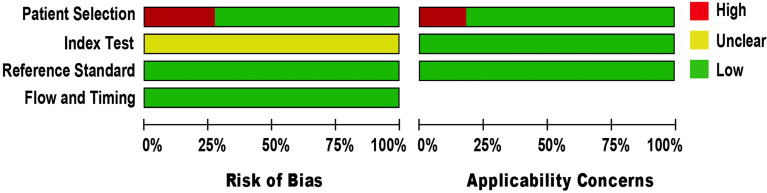
Methodological quality and risk of bias assessment of the eligible studies.

The methodological quality of eligible studies was determined using QUADAS-2 (*see* **Figure 2**). Three studies demonstrated a high risk of bias in the domain of patient selection, as the studies were unable to prevent improper sample exclusion (30, 33, 34) while two studies (26, 35) were partial in their patient selection, this could introduce a high risk of bias in the domain of patient selection applicability (*see* **Supplementary Figure 1** **)**. The risk of bias in the index test domain was unclear because the studies did not report on index test blinding (25–35). The index test’s applicability was not a major concern since there isn’t a globally accepted test methodology. The reference standard domain was supposedly at low risk of bias, as NAAT used pre-established binary response investigation criteria. All studies’ reference standards were performed in either a tertiary care center or a reference laboratory; thus, we expect operator error bias to be of low concern. Subsequently, there was no room for bias in the flow and timing domains since the index test and reference standards were conducted on identical samples. All articles met the criteria for the three domains of applicability concerns, as the majority of studies used osteoarticular samples from patients suspected of having BJI, indicating a low risk of bias.

### Summary Estimates

**Figure 3:**
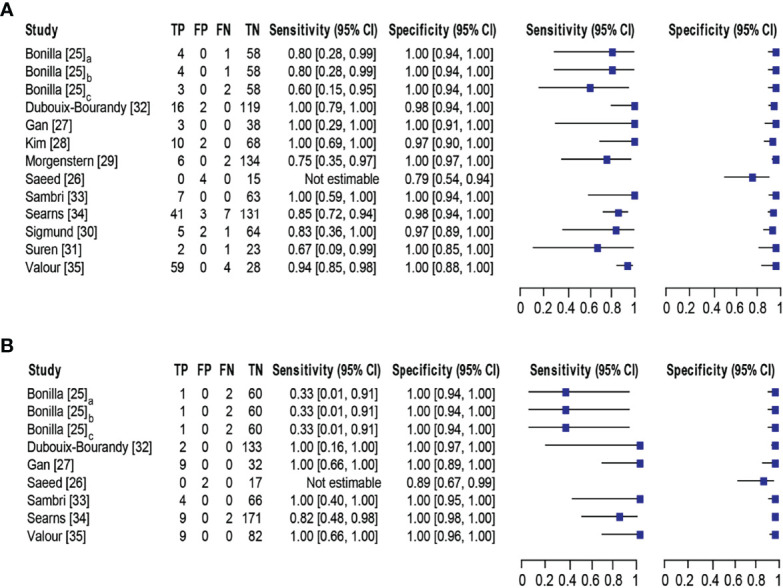
Forest plot for detection of **(A)** MSSA and **(B)** MRSA. Bonilla [25] comprises three datasets that have been designated as Bonilla [25]_a_, Bonilla [25]_b_, and Bonilla [25]_c_ to distinguish them. Bonilla [25]_a_, Bonilla [25]_b_, and Bonilla [25]_c_ compares the sensitivity/specificity of conventional PCR, LightCycler PCR, and TaqMan PCR to microbiological culture, respectively. The black line shows the study’s confidence interval, while the square reflects its sensitivity and specificity. Abbreviations: TP, true positive; FP, false positive; FN, false negative; TN, true negative; CI, confidence interval.

**Figure 4:**
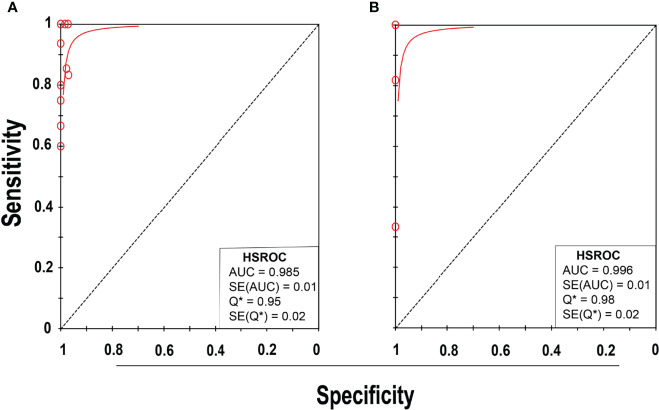
SROC plot of NAAT for **(A)** MSSA and **(B)** MRSA detection. Red circles represent each investigation’s data point, while the solid blue line shows the SROC curve.

Eleven studies (25–35) with 13 datasets comprising 1047 samples met the inclusion criteria for comparing NAAT with a microbiological culture for MSSA detection in suspected BJI patients. The NAAT’s MSSA detection sensitivity and specificity ranged from 0.0 to 1.0 (0.79–1.0) and from 0.79 (0.54–0.94) to 1.00 (0.97–1.0), respectively (*see* **Figure 3A**). The pooled sensitivity was 0.89 (95% CI 0.84–0.93), and pooled specificity was 0.99 (95% CI 0.97–0.99) (*see* **Supplementary Figures 2A,B** **)**. The pooled positive likelihood ratio (PLR) for NAAT was 34.13 (95% CI 20.54–56.73), and the pooled negative likelihood ratio (NLR) for NAAT was 0.19 (95% CI 0.12–0.3) (*see* **Supplementary Figures 2C, D**). Additionally, the pooled DOR of NAAT was 283.37 (95% CI 129.49–620.08) (*see* **Supplementary Figure 2E** **)**. The DOR (283.37 >1) indicated that NAAT was effective in our study. The statistical values for *I* ^2^ sensitivity and specificity were 31.3% and 62.4%, respectively (*see* **Supplementary Figures 2A,B** **)**, suggesting low to moderate heterogeneity. The summary receiver operating characteristics (SROC) area under the curve (AUC) was 0.98 (95% CI 0.97–1.0), suggesting fairly decent diagnostic validity (**Figure 4A**).

Concerning MRSA detection (25–27, 32–35), a total of seven studies involving nine datasets and 727 samples evaluated the accuracy of NAAT against a microbiological culture reference standard. NAAT’s sensitivity and specificity for detecting MRSA ranged from 0.0 to 1.0 (95% CI 0.66–1.0) and 0.89 (95% CI 0.67–0.99) to 1.00 (95% CI 0.98–1.00), respectively (*see* **Figure 3B**). The pooled sensitivity was 0.81 (95% CI 0.67–0.91), and pooled specificity was 1.0 (95% CI 0.99–1.0) (*see* **Supplementary Figures 3A, B** **)**. The pooled PLR for NAAT was 62.1 (95% CI 24.47–157.6), and the pooled NLR for NAAT was 0.33 (95% CI 0.16–0.69) (*see* **Supplementary Figures 3C, 3D** **)**. Additionally, the pooled DOR of NAAT was 300.25 (95% CI 85.01–1060.5) (*see* **Supplementary Figure 3E** **)**. In this study, the DOR (300.25 >1) indicated that the NAAT was effective. The sensitivity and specificity of MRSA detection have *I* ^2^ statistical values of 60.7% and 45.0%, respectively (*see* **Supplementary Figures 3A, B** **)**, inferring moderate heterogeneity. The AUC of SROC for MRSA was 1.0 (95% CI 0.99–1.0), indicating that the diagnostic validity was overall acceptable (**Figure 4B**).

### Diagnostic Accuracy of In-House vs. Commercial Tests

**Table 2** summarizes the diagnostic accuracy of research findings based on different NAA tests (*see* **Supplementary Figures 4–12** **)**. The pooled estimates of the in-house NAA tests for MSSA detection [sensitivity: 0.85 (95% CI 0.66–0.95), specificity: 0.98 (95% CI 0.96–0.99), PLR: 29.39 (95% CI 9.53–90.59), NLR: 0.30 (95% CI 0.16–0.55), DOR: 204.5 (95% CI 49.72–841.43) and AUC: 0.97 (95% CI 0.95–0.99)] were slightly higher than those for MRSA [sensitivity: 0.66 (95% CI 0.41–0.86), specificity: 0.99 (95% CI 0.97–1.0), PLR: 25.76 (95% CI 7.36–90.18), NLR: 0.56 (95% CI 0.31–1.0), DOR: 84.32 (95% CI 16.41–433.24) and AUC 0.98 (95% CI 0.94–1.0)] (*see* **Supplementary Figures 4, 5** **)**. The pooled summary estimates of the commercial tests for MSSA identification are shown in **Table 2** (*see* **Supplementary Figure 6** **)**. Among NAA tests, GeneXpert was consistently applied in osteoarticular samples to detect staphylococcal strains in BJI (*see* **Table 2** **)**.

**Table 2:** Subgroup analysis of studies using various NAA tests.

DiagnosticTarget against culture reference standard	Subgroup	NAAT methods	No. of data	% Sensitivity (95% CI)	% Specificity(95% CI)	PLR (95% CI)	NLR (95% CI)	DOR (95% CI)	AUC (95% CI)
***S. aureus***	In-house		6	85 (66-99)	98 (96-99)	29.39 (9.5-90.6)	0.3 (0.16-0.55)	204.5 (49.72-841.43)	97 (95-98)
		RT-PCR	3	68 (35-92)	97 (93-99)	21.26 (1.61-281.53)	0.39 (0.19-0.8)	75.77 (5.67-1011.1)	49 (35-96)
		mPCR	2	100 (75-100)	98 (94-100)	31.5 (10.27-96.59)	0.08 (0.01-0.51)	561.7 (47.58-6631.9)	–
		PCR	1	80 (28-100)	100 (94-100)	88.5 (5.39-1453.1)	0.25 (0.06-1.01)	351.0 (12.4-9916.1)	–
	Commercial		7	90 (84-94)	99 (97-1.0)	38.72 (20.52-73.06)	0.15 (0.09-0.26)	327.3 (127.8-838-2)	99 (97-99)
		Xpert	4	92 (86-96)	99 (97-100)	46.5 (21.66-99.76)	0.1 (0.06-0.18)	445.7 (145.2-1368.3)	99 (98-100)
		mPCR-UITI	3	77 (50-93)	99 (96-100)	31.0 (7.04-136.17)	0.29 (0.14-0.6)	157.59 (28.1-884.9)	92 (82-99)
**MRSA**	In-house		5	66 (41-86)	99 (97-100)	25.76 (7.36-90.18)	0.56 (0.31-1.0)	84.32 (16.41-433.2)	98 (96-99)
		RT-PCR	3	36 (7-77)	99 (95-100)	16.41 (3.34-80.6)	0.63 (0.37-1.05)	42.22 (5.15-346.07)	61 (11-80)
		mPCR	1	100 (66-100)	100 (89-100)	62.7 (3.99-985.1)	0.05 (0.0-0.76)	1235.0 (22.9-66493.8)	–
		PCR	1	33 (1-90)	100 (94-100)	45.8 (2.2-953.1)	0.63 (0.3-1.35)	72.6 (2.32-2267.6)	–
	Commercial		4	92 (75-99)	100 (99-100)	184.1 (45.7-740.7)	0.16 (0.07-0.39)	1560.1(241.6-10075.9)	99 (98-100)
		Xpert	4	92 (75-99)	100 (99-100)	184.1 (45.7-740.7)	0.16 (0.07-0.39)	1560.1(241.6-10075.9)	99 (98-100)

-, not estimable; AUC, area under the curve; DOR, diagnostic odds ratio; mPCR, multiplex PCR; NAAT, nucleic acid amplification tests; PCR, polymerase chain reaction; NLR, negative likelihood ratio; PLR, positive likelihood ratio; qPCR, quantitative PCR; RT-PCR, real-time PCR.

### Meta-Regression and Subgroup Analysis

The potential cause of heterogeneity was investigated using a meta-regression analysis on pre-specified subgroups. According to the findings of the meta-regression analysis, country (developing *vs* developed), setting (tertiary care center *vs* reference laboratory), study design (prospective *vs* others), patient selection (consecutive *vs* convenience), and sample condition (fresh *vs* frozen) were not significant sources of heterogeneity (meta-regression *P* = 0.77, *P* = 0.48, *P* = 0.76, *P* = 0.69, and *P* = 0.84, respectively) (*see* **Supplementary Figure 13** **)**.

### Publication Bias

Deek’s funnel plot asymmetry test was utilized to evaluate publication bias. In this study, we did not detect striking publication bias (*P* = 0.1) (*see* **Supplementary Figure 14** **)**.

## Discussion

Identifying the causal organism and using adequate antibiotic therapy are essential in the management of staphylococcal BJI. The traditional bacterial culture and Gram stain testing, both of which have low to intermediate sensitivity, obscure the treatment plan of staphylococcal BJI (6, 42), causing a delay in the administration of antibacterial drugs against the offending microorganisms. In general, inadequate source management, underlying comorbidities, or delays in delivering definitive BJI treatment have been associated to increased recurrence, poor prognosis, and death (1, 6). Therefore, in patients with suspected staphylococcal BJI, it is essential to identify staphylococcal species and resistance indicators as soon as possible, as an early response may substantially improve overall survival rates and decrease hospital burden. Several recent investigations have indicated that NAAT is a potential technique for differentiating staphylococcal BJI from other pathogenic organisms (25–35). However, the results of these research have not been thoroughly reviewed. Therefore, we performed a meta-analysis to evaluate NAAT’s diagnostic performance in detecting staphylococcal BJI in clinically suspected patients.

**Figure 5:**
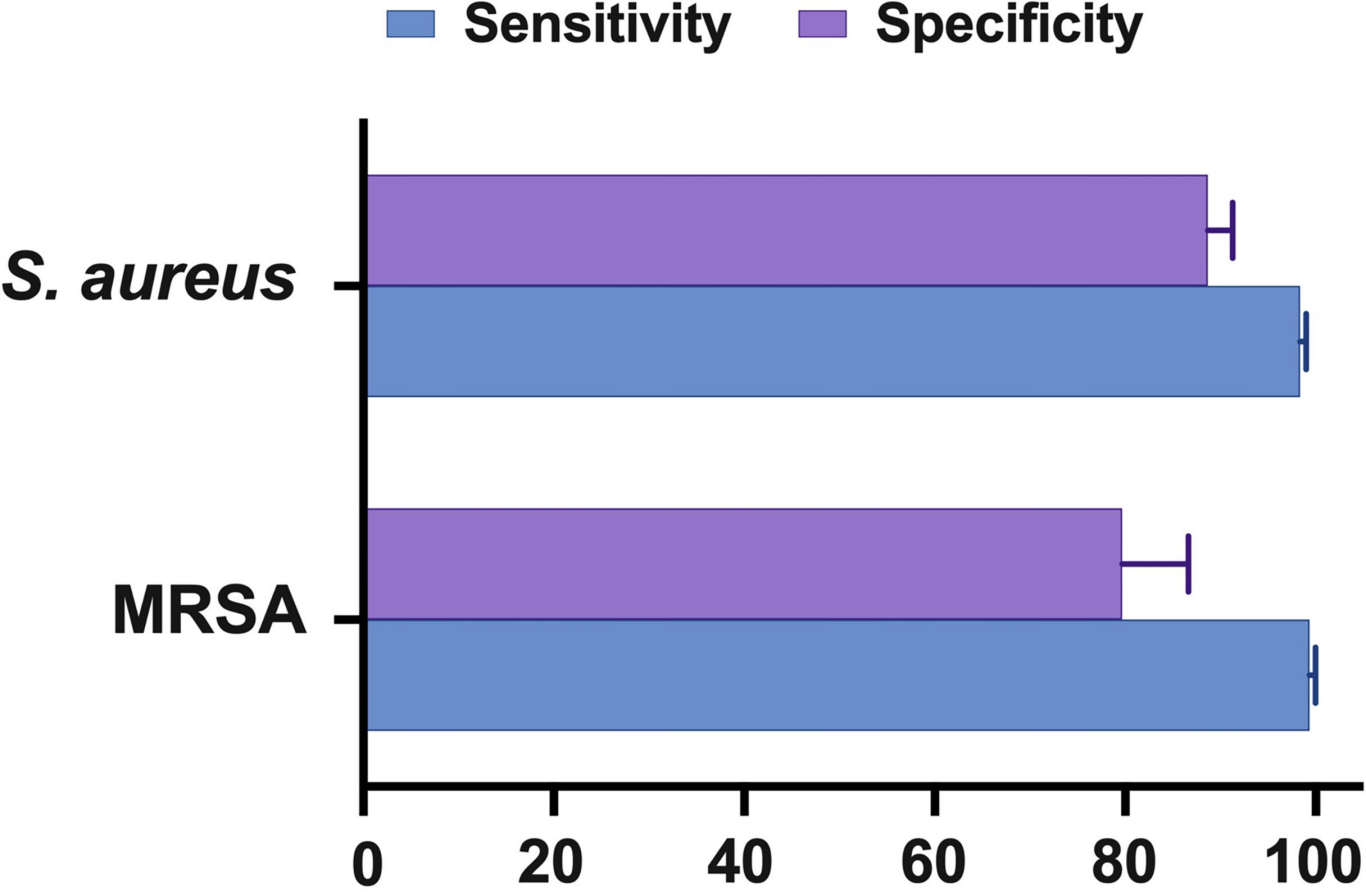
Summary of NAAT’s pooled sensitivity and specificity.

According to the findings of this study, the NAAT overall summary estimates for MSSA [sensitivity: 0.89 (95% CI 0.84–0.93), specificity: 0.99 (95% CI 0.97–0.99), PLR: 34.13 (95% CI 20.54–56.73), NLR: 0.19 (95% CI 0.12–0.3), and DOR: 283.37 (95% CI 129.49–620.1)] and MRSA [sensitivity: 0.81 (95% CI 0.67–0.91), specificity: 1.0 (95% CI 0.99–1.0), PLR: 62.1 (95% CI 24.5–157.6), NLR: 0.33 (95% CI 0.16–0.69), and DOR: 300.25 (95% CI 85.01–1060.5)] detection were comparable (*see* **Supplementary Figures 2**, **3** **)**, which is consistent with other scientists’ independent investigations in BJI (33, 34). NAAT had a greater sensitivity for identifying MSSA and MRSA than microbiological culture, which may be ascribed to factors such as antibiotic pre-administration, low microbial content in osteoarticular fluid samples, and stringent laboratory technique. **Figure 5** depicts NAAT’s cumulative sensitivity and specificity for identifying staphylococcal isolates in suspected BJI patients.

We found that NAAT detected more infectious organisms than microbiological cultures alone, and that the test’s specificity (>93%) was much greater for both MSSA and MRSA detection, implying that NAAT diagnostic accuracy was higher. It could be possibly due to NAAT, unlike traditional culture, relies on detecting bacterial DNA rather than the recovery of viable bacteria, and is less affected by biofilm extracellular polymeric matrix and antibiotic preadministration. Since it gives fast results and can identify dead microbes, we suggest utilizing NAAT in principle to diagnose staphylococcal BJI. In comparison to previously published systematic reviews, we found that the meta-analysis by Chen et al. reported on the greater diagnostic value of NAAT for staphylococcal strains in LRT specimens (22) and blood specimens (23), which is consistent with the findings of this study. However, these reviews focused solely on the detection of MSSA or MRSA in fluids other than osteoarticular samples.

Additionally, NAAT subgroup analysis revealed that commercial tests for detection of both MSSA [sensitivity: 0.9 (95% CI 0.84–0.94), specificity: 0.99 (95% CI 0.97–1.00)] and MRSA [sensitivity: 0.92 (95% CI 0.75–0.99), specificity: 1.00 (95% CI 0.99–1.00)] were higher than overall diagnostic accuracy of NAAT (**Table 2**). The PLR for the commercial test was markedly higher, indicating that individuals with staphylococcal BJI are more likely to have a positive NAA test than those who do not have BJI. The sensitivity of the in-house staphylococcal strain detection test was lower than that of commercial assays. **Table 2** shows the pooled summary estimates for MSSA detection from independent commercial testing. Xpert was commonly used in NAAT studies to identify staphylococcal BJI, and mPCR demonstrated the highest diagnostic accuracy in osteoarticular fluids. In our study, countries, settings, study design, patient selection, and sample conditions were not shown to be major drivers of heterogeneity (*P >*0.05) (*see* **Supplementary Figure 13**).

Our study’s merits include a comprehensive search strategy that found all relevant articles from five of the most often used databases, with no language restrictions. The searches were carried out in a methodical manner, and the titles and abstracts of all papers were evaluated by at least two researchers. Following a group discussion, the authors’ collective judgment was reflected in the papers included in this systematic review. The PRISMA criteria for systematic reviews were followed in this research, and the QUADAS-2 tool was utilized to evaluate the methodological quality of the included publications. The following analysis eliminated studies that did not adhere to certain criteria for identifying staphylococcal BJI. For data scrutiny, this research utilized a bivariate random-effects model and meta-regression analysis on specified subgroups. Furthermore, studies combining nucleic acid amplification with sequencing and enrichment stages before molecular testing were omitted since they may exaggerate the index test’s diagnostic performance.

There are a few limitations to this research that should be considered. We are likely to have overlooked a few significant research by conducting comprehensive literature searches across databases. The subgroup and meta-regression analysis showed that factors such the NAA methods and standard tests may be the source of the variability. We were unable to examine the effect of variables including sample volume, gene target, primers utilized, amplification procedures, processing stages, individual experience with NAA testing, and laboratory infrastructure on NAA test accuracy owing to a high degree of variability in these parameters and/or reporting these factors in the studies. In addition, this meta-analysis was constrained due to a limited number of studies evaluating the accuracy of molecular tests in osteoarticular specimen and should be interpreted with caution. Lastly, as with any meta-analysis, possible publication bias was a matter of concern.

## Conclusions

The findings of this study suggest that using NAAT on osteoarticular samples may be helpful as a rule-in test for therapeutic interventions in BJI patients. Furthermore, future study should examine other metrics, such as NAAT’s influence on cost-effectiveness, reduced hospitalizations, and adverse antimicrobial effects, to enable treatment adjustments.

## Data Availability Statement

The original contributions presented in the study are included in the article/**Supplementary Material**. Further inquiries can be directed to the corresponding author.

## Author Contributions

KC and SCO conceptualized the study. KC, YW, and YY performed the literature search, analyzed data, and drafted the manuscript. WQ, Y-JS, SA, CS, C-LD, and SCO reviewed and edited the manuscript. All authors read and approved the final manuscript.

## Funding

This study was funded in part by the Doctoral Research Fund of Affiliated Hospital of Southwest Medical University to SCO and KC.

## Conflict of Interest

The authors declare that the research was conducted in the absence of any commercial or financial relationships that could be construed as a potential conflict of interest.

## Publisher’s Note

All claims expressed in this article are solely those of the authors and do not necessarily represent those of their affiliated organizations, or those of the publisher, the editors and the reviewers. Any product that may be evaluated in this article, or claim that may be made by its manufacturer, is not guaranteed or endorsed by the publisher.
