# Supplementary material for: Molecular Tools for Guiding Therapy in Patients With Staphylococcal Bone and Joint Infections: A Diagnostic Test Accuracy Meta-analysis
Source: Front Endocrinol (Lausanne). 2022 Jul 13;13:792679. doi: 10.3389/fendo.2022.792679 (PMC9326260; doi:10.3389/fendo.2022.792679)
Supplement: Supplementary file 1 [file DataSheet_1.zip › Supplementary figures & table.docx]

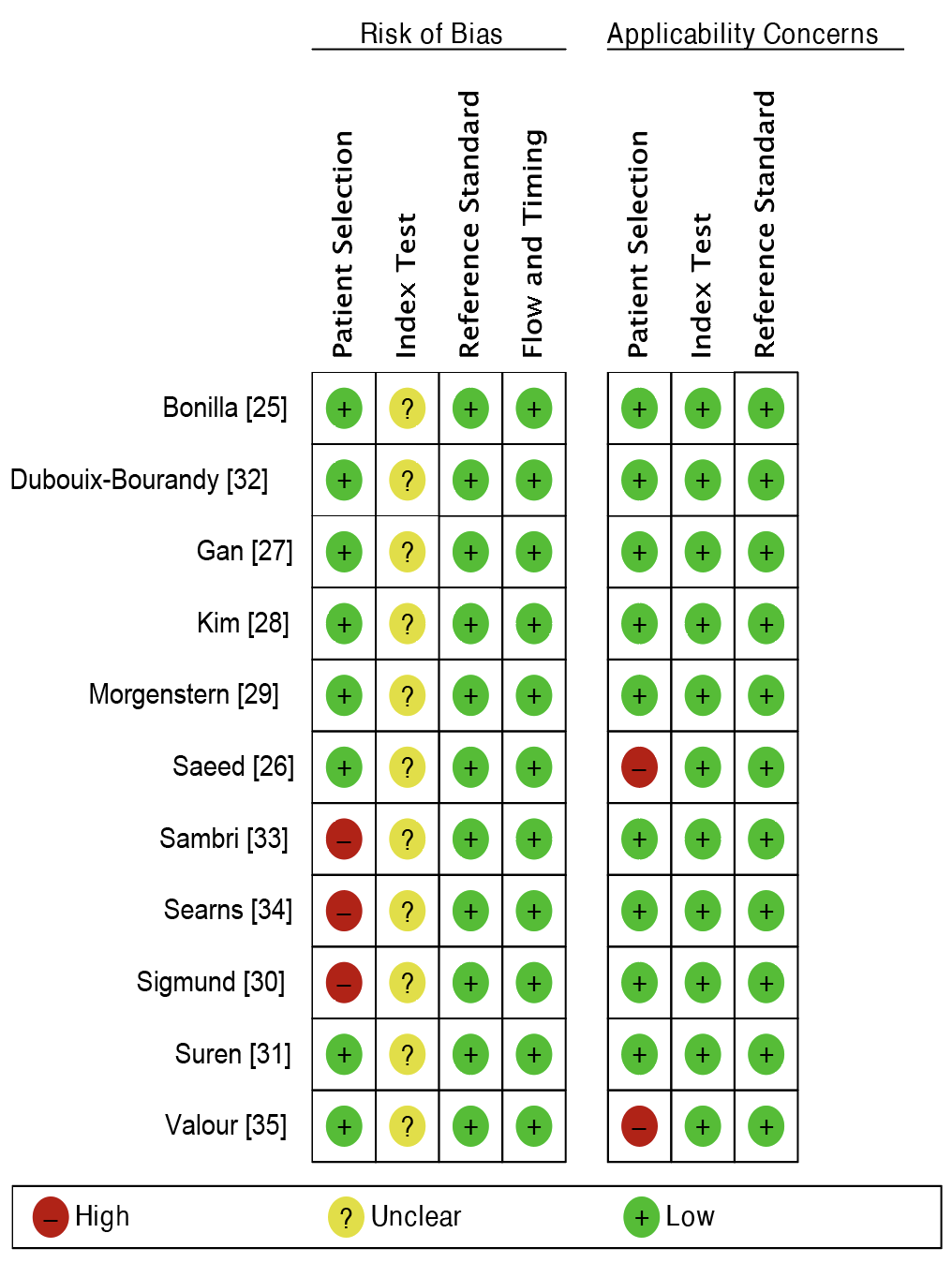


**﻿Supplementary Fig. 1** Individual study methodological quality and risk of bias assessment using the QUADAS-2 tool.

A


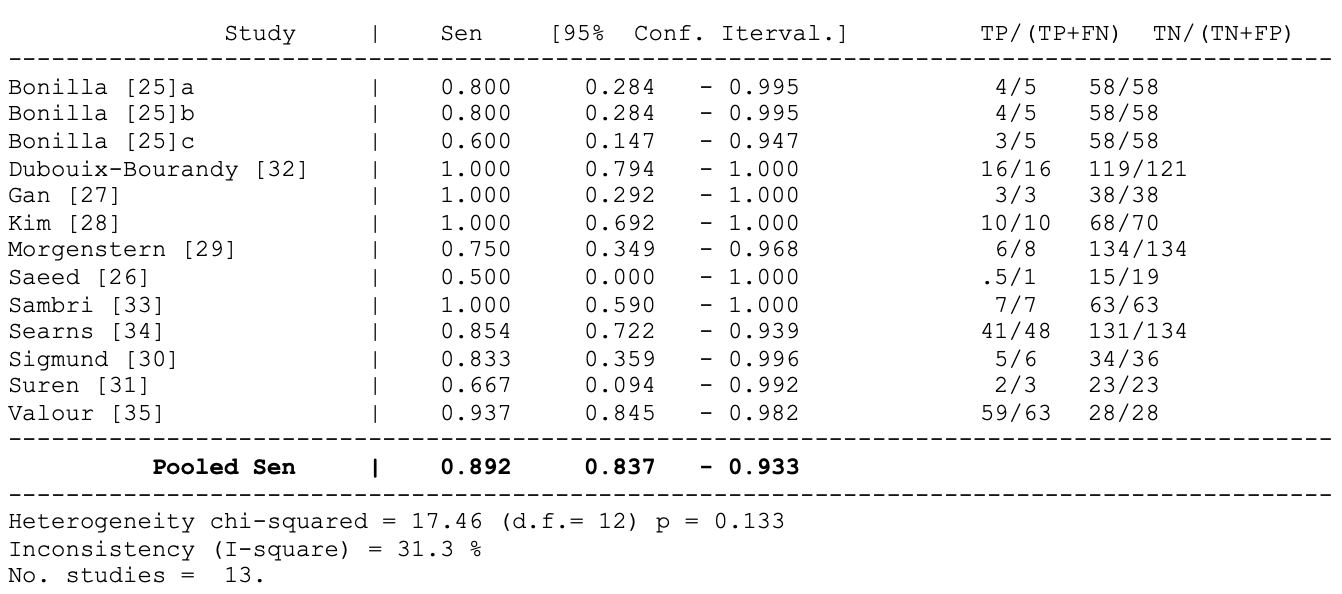


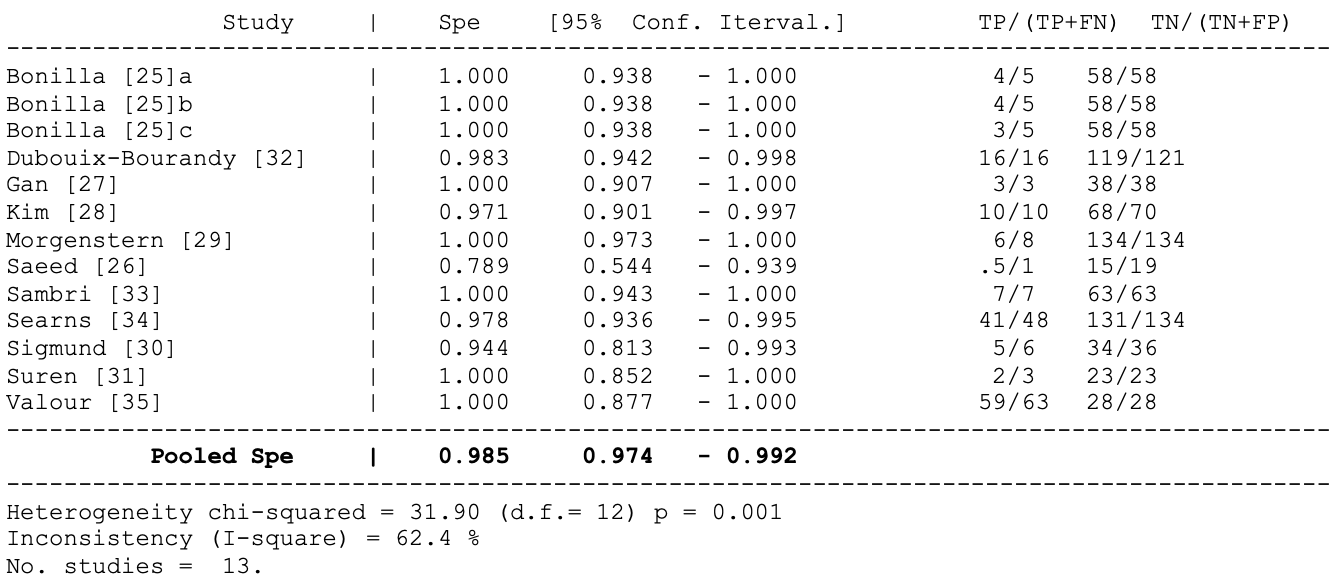


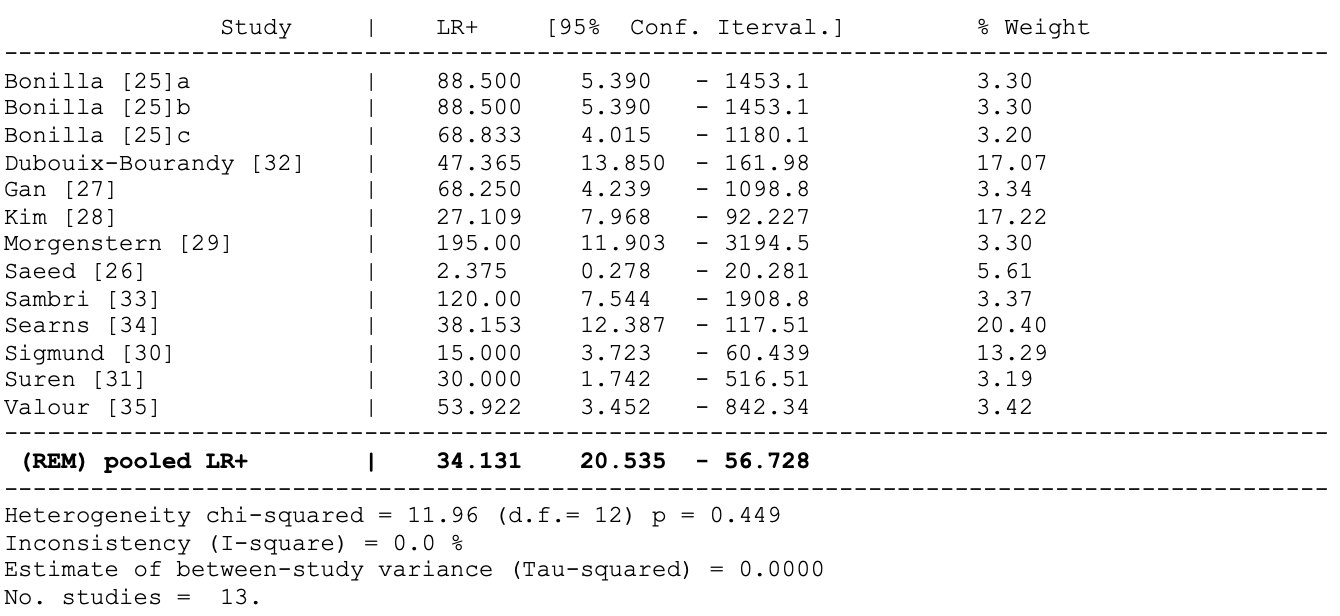


B

C

D


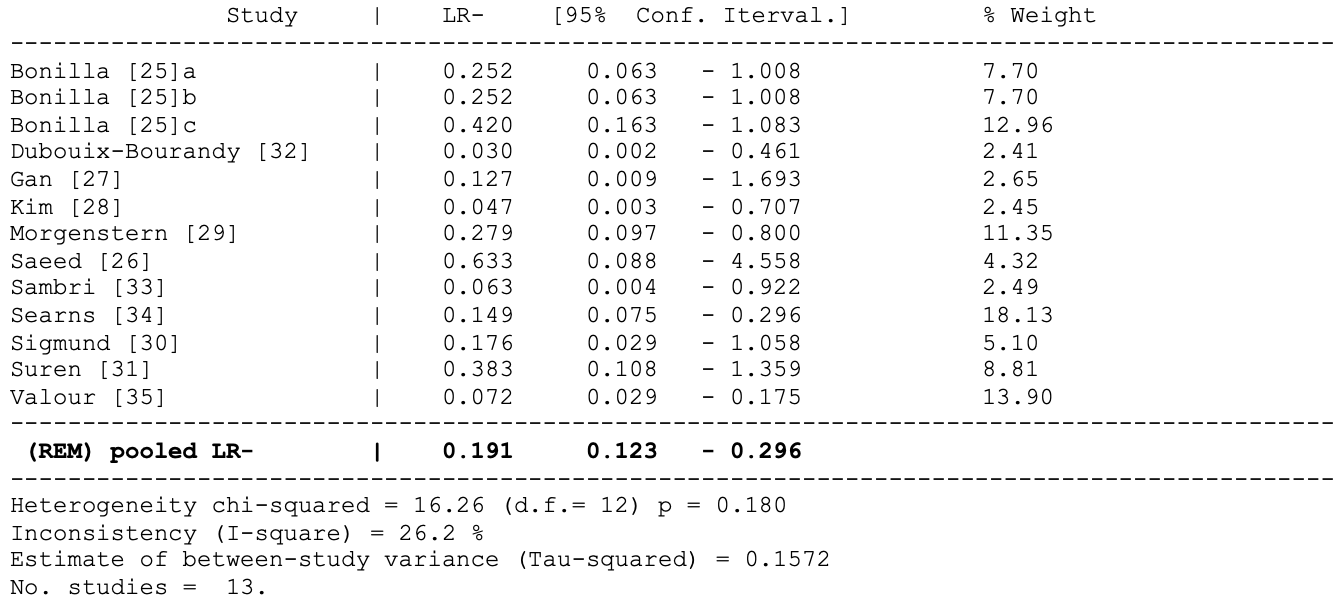


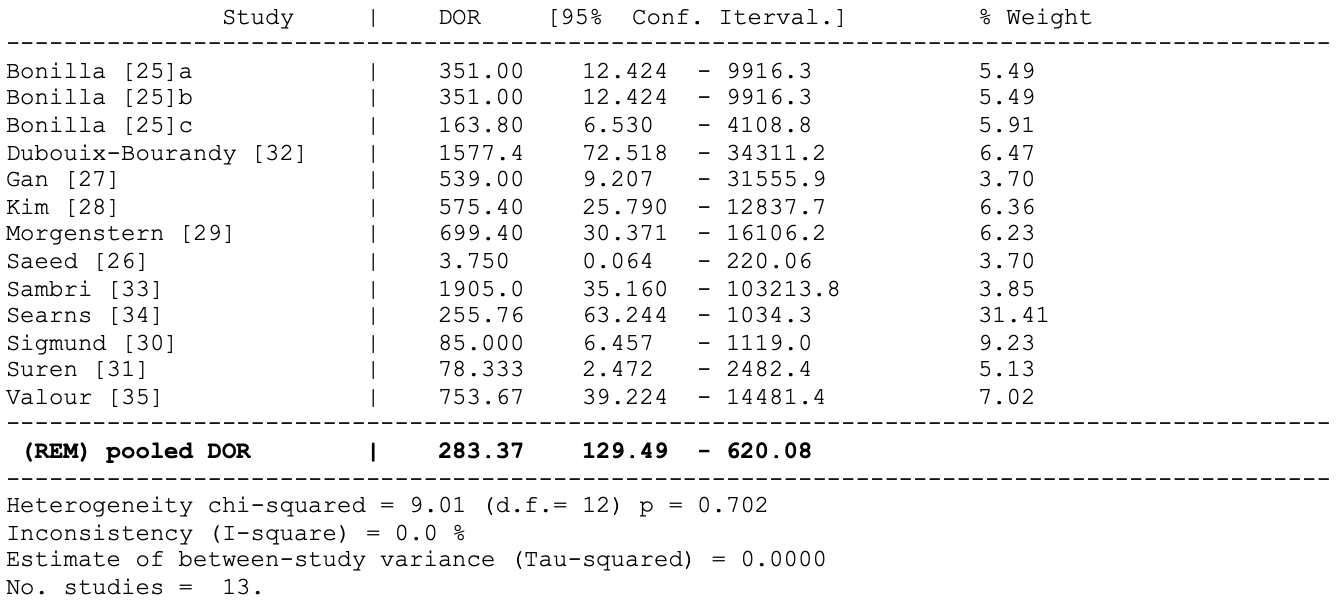


E

**Supplementary Fig. 2 Pooled summary estimates of NAAT for methicillin-sensitive *S. aureus* (MSSA)** detection. (A) pooled sensitivity (B) pooled specificity (C) pooled positive likelihood ratio (PLR) (D) pooled negative likelihood ratio (NLR) (E) pooled diagnostic odds ratio (DOR). Note that Bonilla [25] comprises three datasets that have been designated as Bonilla [25]a, Bonilla [25]b, and Bonilla [25]c to distinguish them. Bonilla [25]a, Bonilla [25]b, and Bonilla [25]c compares the sensitivity/specificity of conventional PCR, LightCycler PCR, and TaqMan PCR to microbiological culture, respectively. It should be noted that the figure of the area under the curve (AUC) has been included in the main text (Figure 4A).

A


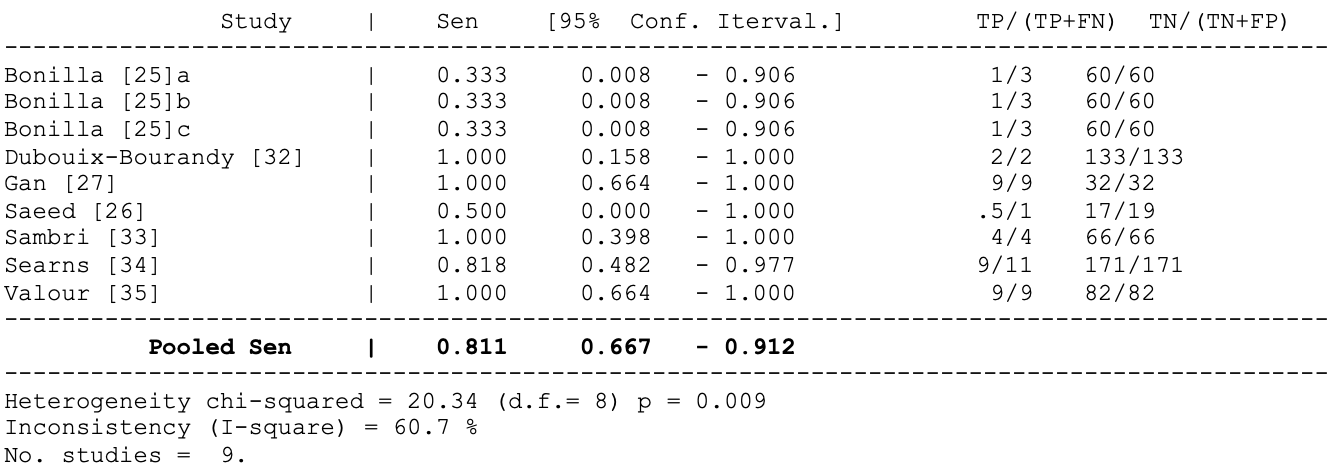


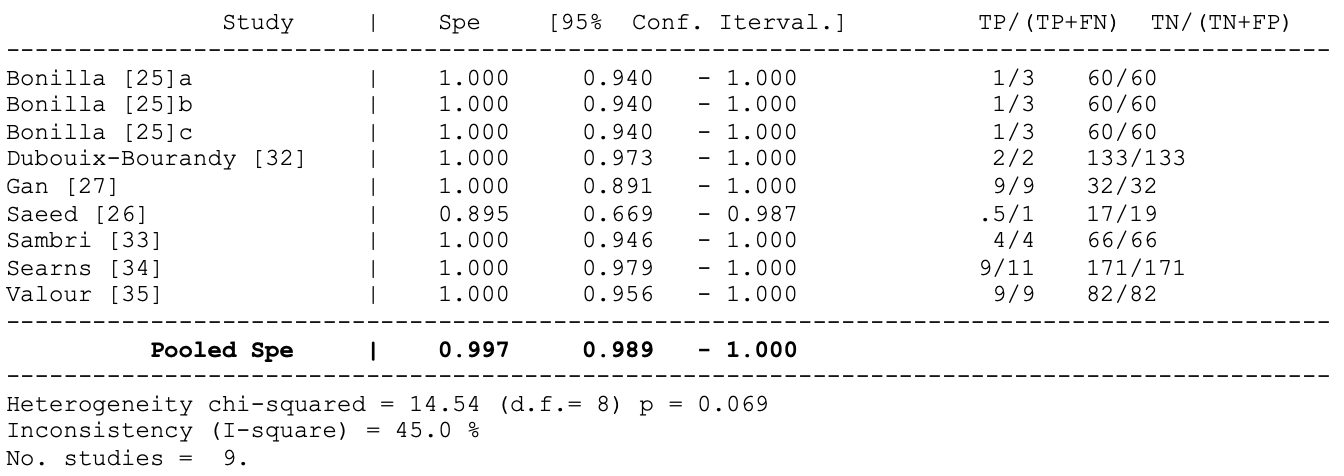


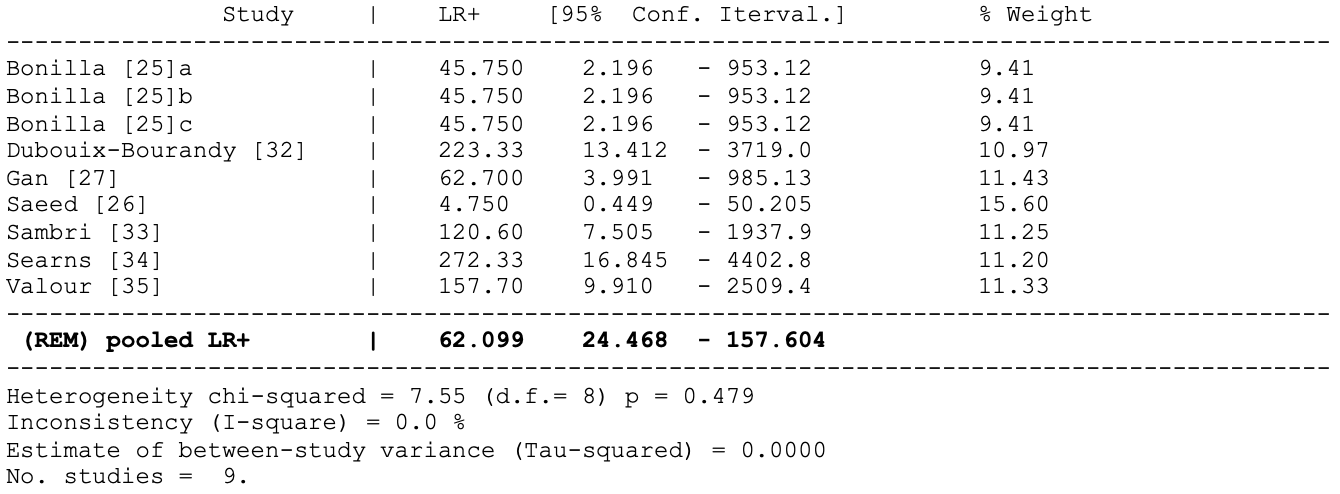


B

C

D


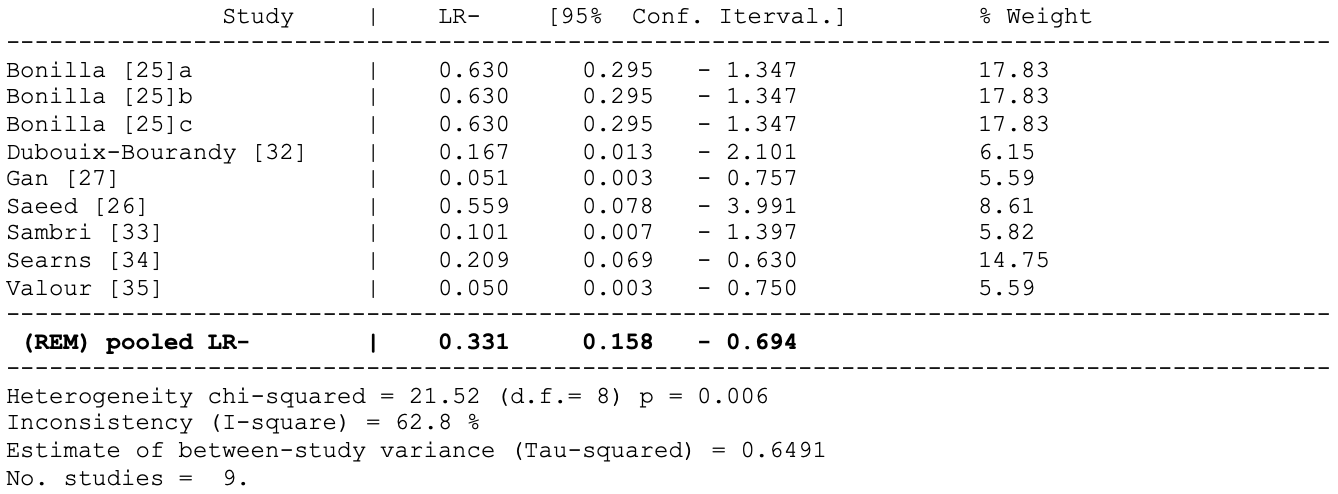


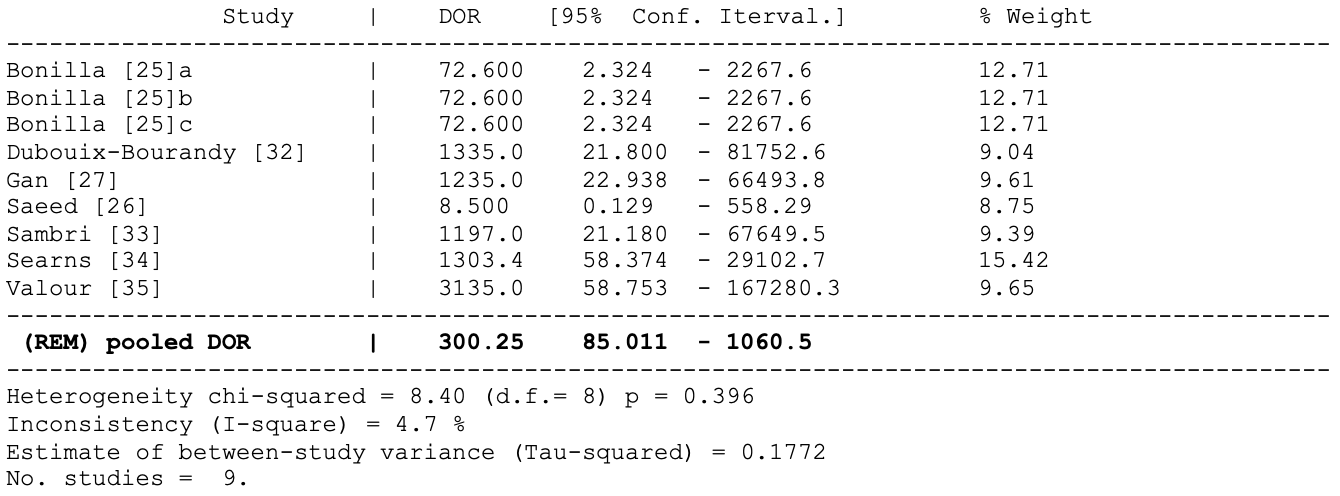


E

**Supplementary Fig. 3 Pooled summary estimates of NAAT for methicillin-resistant *S. aureus* (MRSA)** detection. (A) pooled sensitivity (B) pooled specificity (C) pooled PLR (D) pooled NLR (E) pooled DOR. Note that Bonilla [25] comprises three datasets that have been designated as Bonilla [25]a, Bonilla [25]b, and Bonilla [25]c to distinguish them. Bonilla [25]a, Bonilla [25]b, and Bonilla [25]c compares the sensitivity/specificity of conventional PCR, LightCycler PCR, and TaqMan PCR to microbiological culture, respectively. It should be noted that the AUC figure is included in the main text (Figure 4B).

A


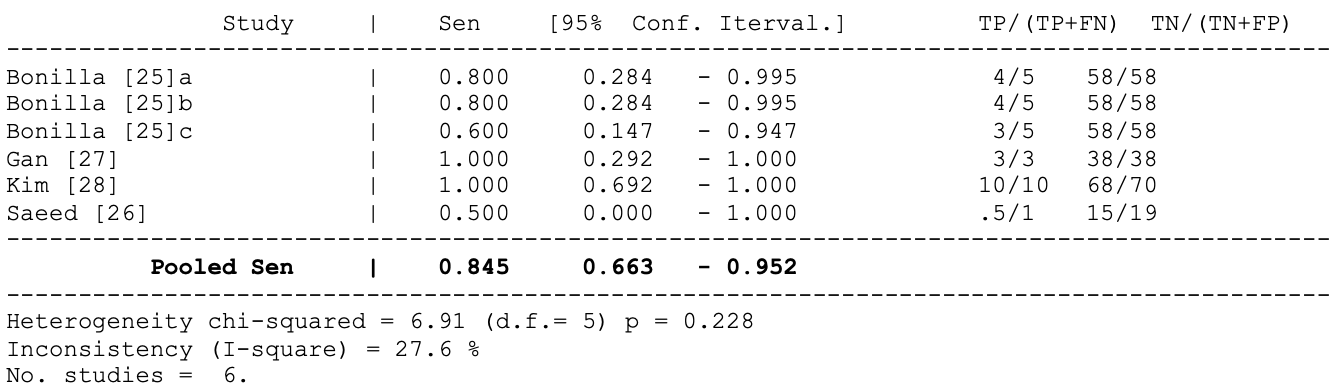


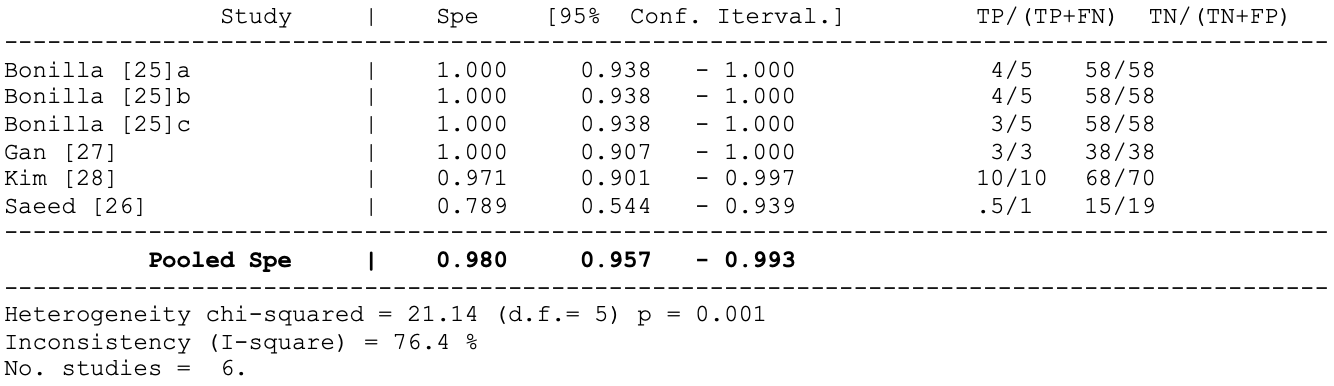


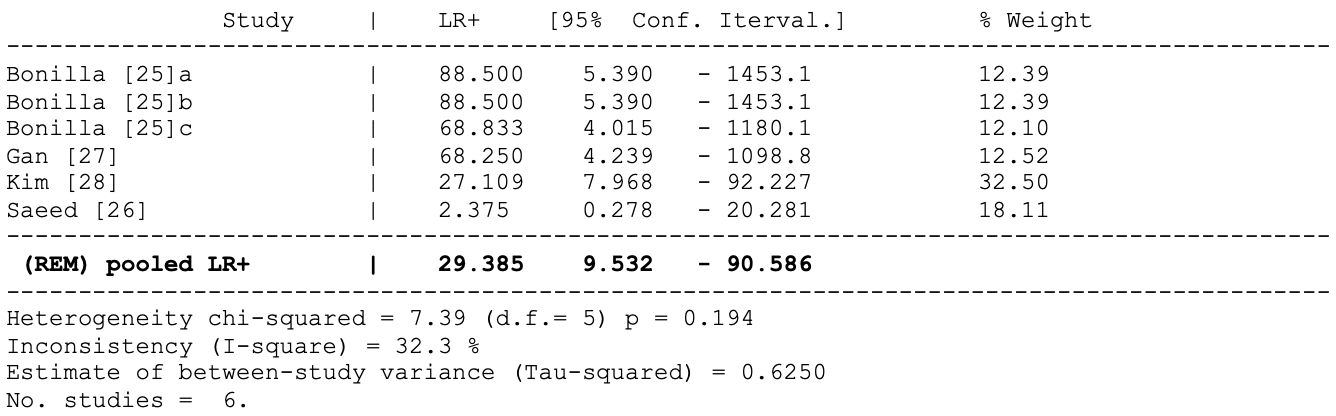


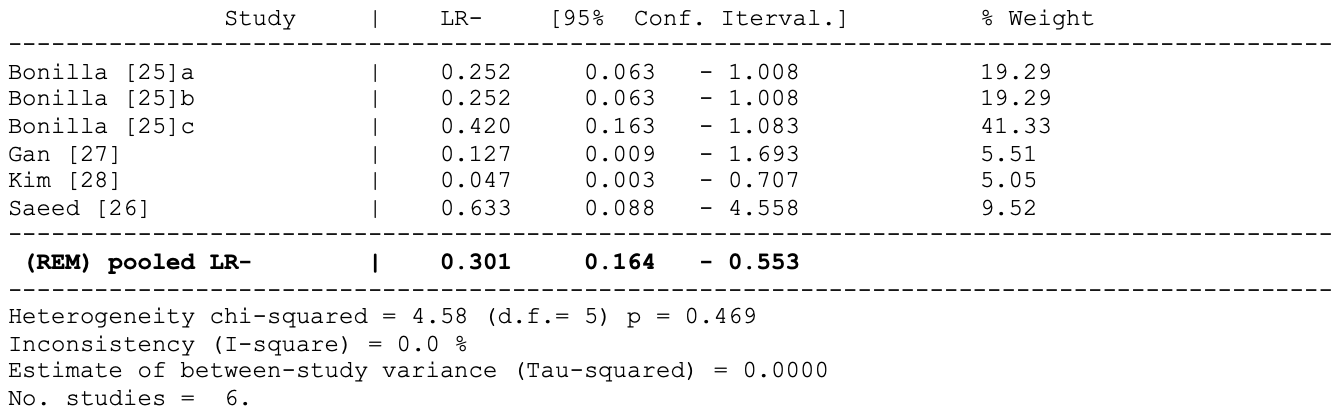


B

C

D

E


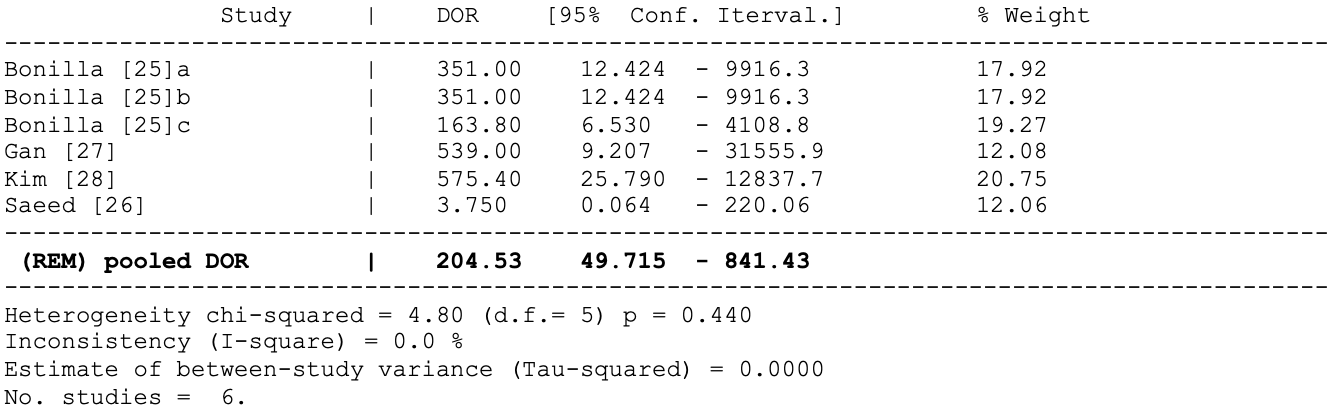

F

**Supplementary Fig. 4 Pooled summary estimates of in-house NAAT for MSSA** detection. (A) pooled sensitivity (B) pooled specificity (C) pooled PLR (D) pooled NLR (E) pooled DOR (F) area under the curve (AUC). It should be noted that Bonilla [25] comprises three datasets that have been designated as Bonilla [25]a, Bonilla [25]b, and Bonilla [25]c to distinguish them. Bonilla [25]a, Bonilla [25]b, and Bonilla [25]c compares the sensitivity/specificity of conventional PCR, LightCycler PCR, and TaqMan PCR to microbiological culture, respectively. It should be noted that some of the datapoints may be obscured due to AUC number overlapping.

A


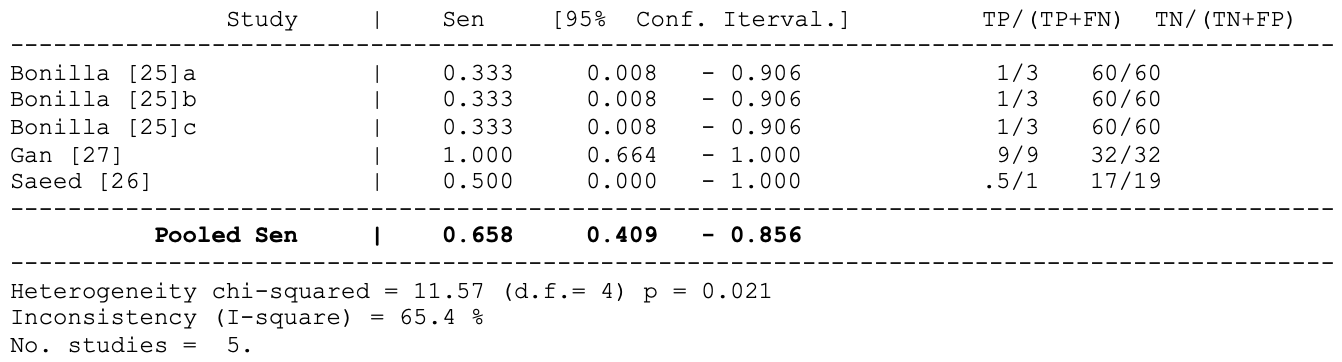


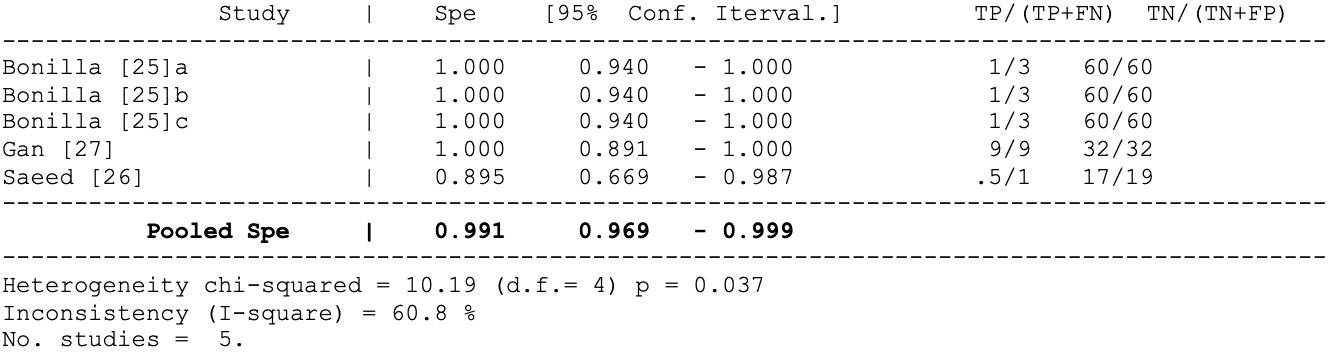


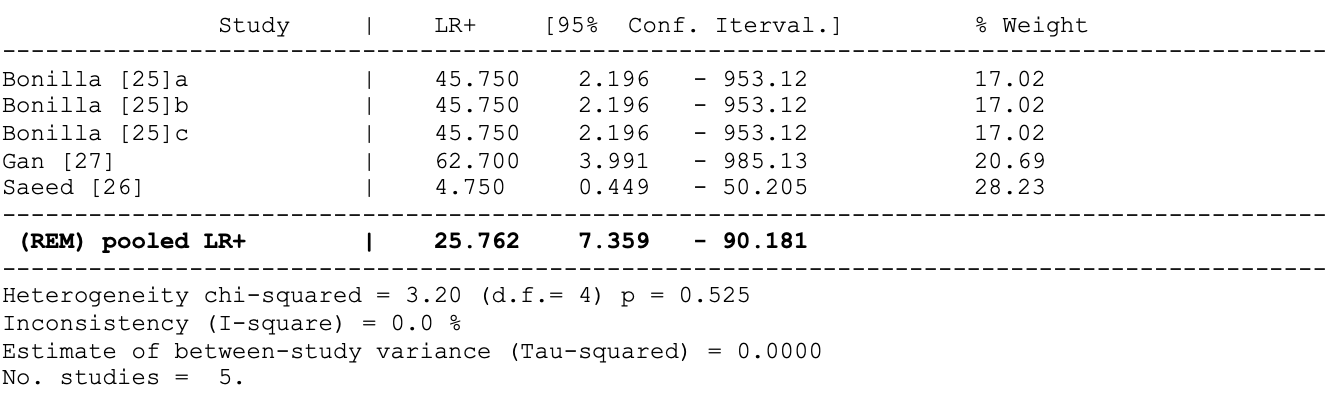


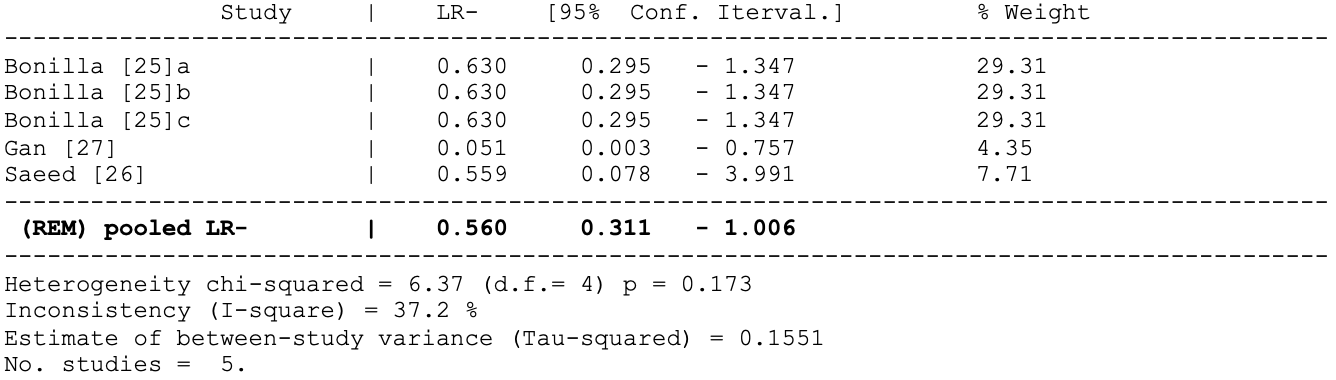


B

C

D


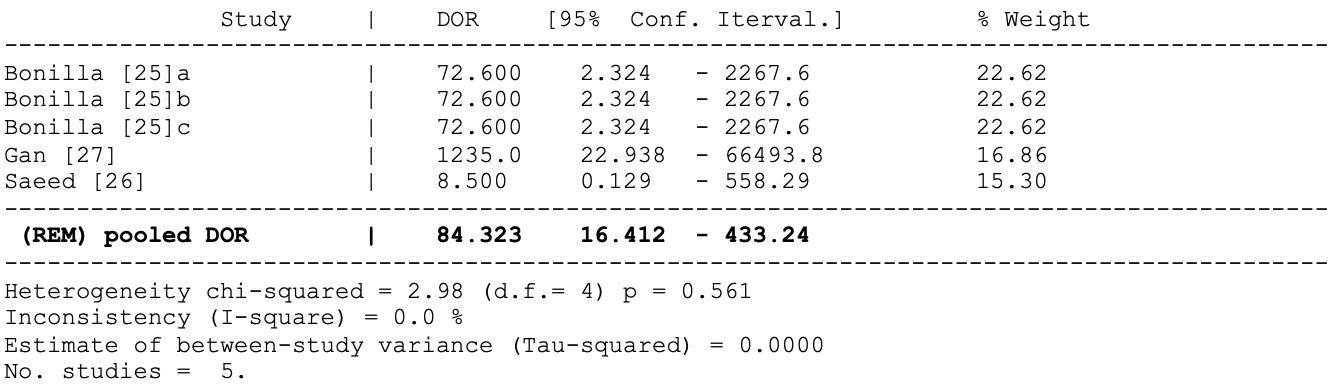


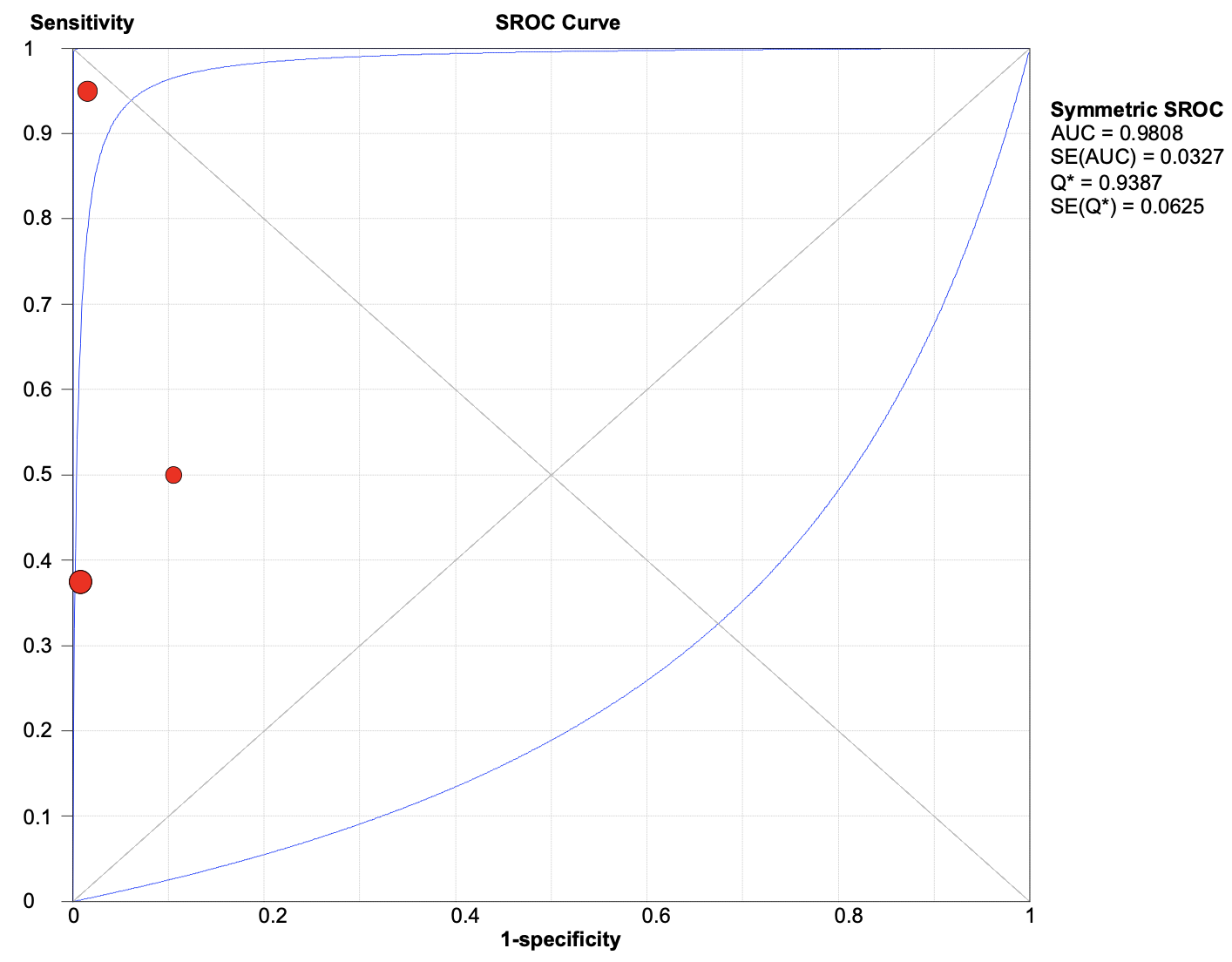


E

F

**Supplementary Fig. 5 Pooled summary estimates of in-house NAAT for MRSA** detection. (A) pooled sensitivity (B) pooled specificity (C) pooled PLR (D) pooled NLR (E) pooled DOR (F) AUC. Note that Bonilla [25] comprises three datasets that have been designated as Bonilla [25]a, Bonilla [25]b, and Bonilla [25]c to distinguish them. Bonilla [25]a, Bonilla [25]b, and Bonilla [25]c compares the sensitivity/specificity of conventional PCR, LightCycler PCR, and TaqMan PCR to microbiological culture, respectively. Note that some of the overlapping of numbers in AUC may be obscured.

A


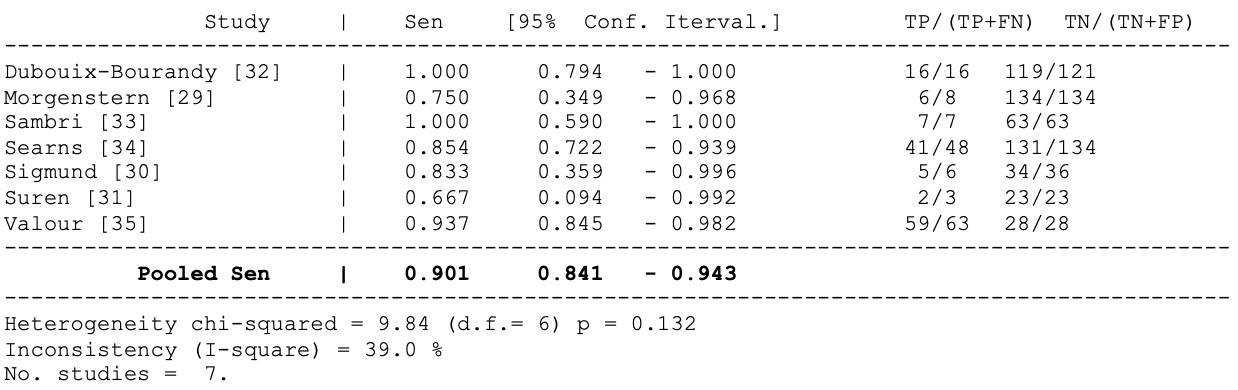


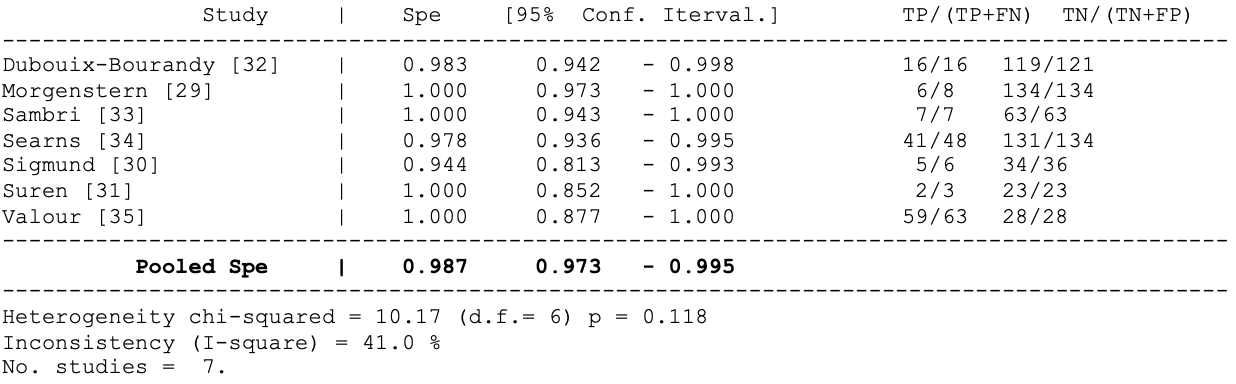


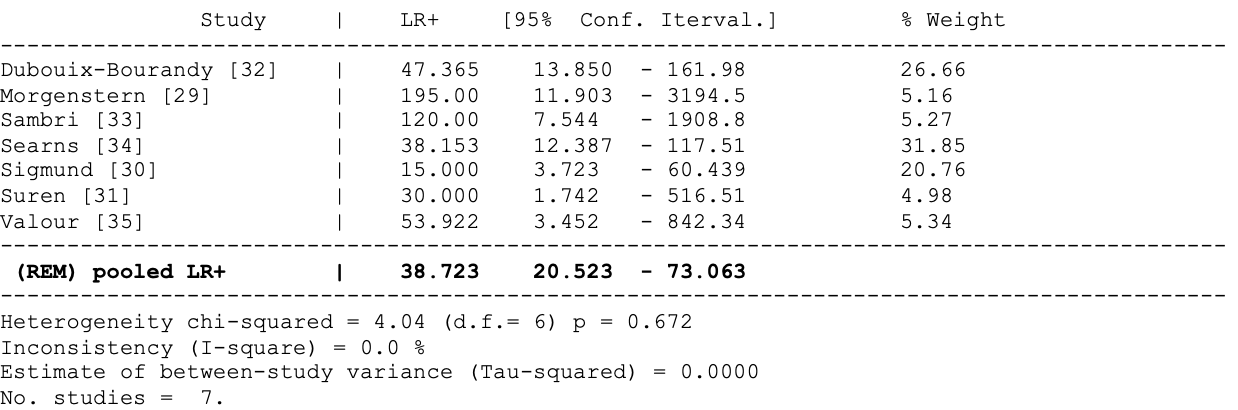


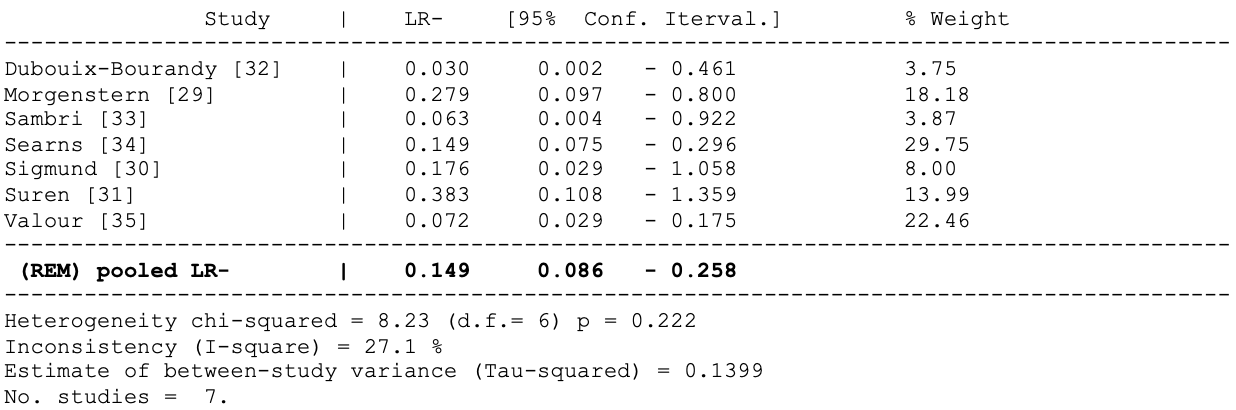


B

C

D


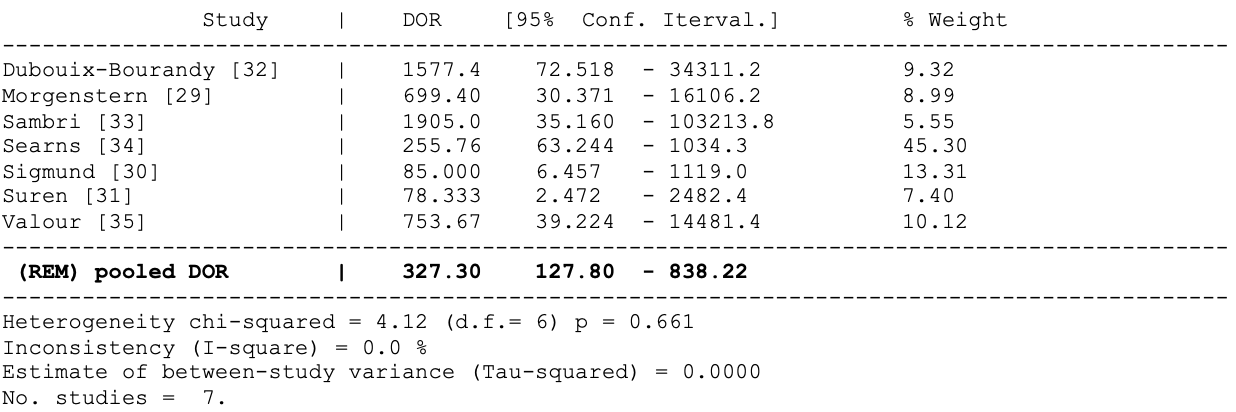


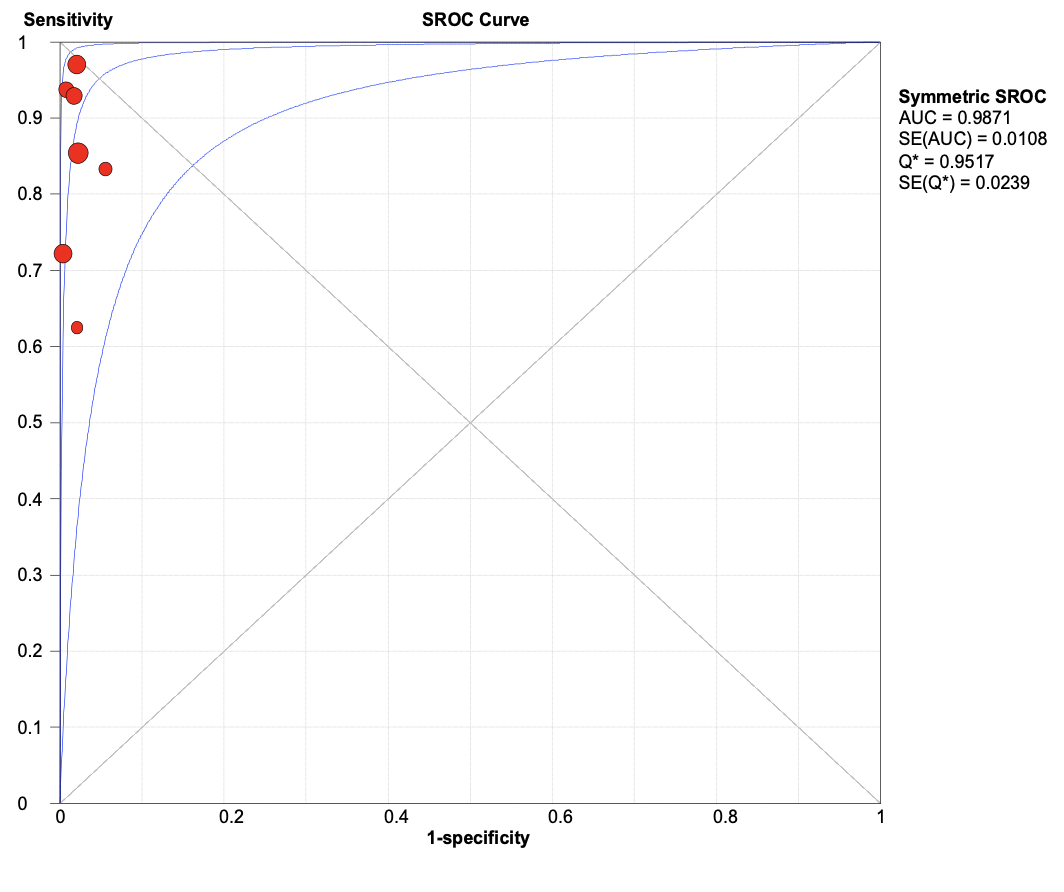


E

F

**Supplementary Fig. 6 Pooled summary estimates of commercial NAAT for MSSA** detection. (A) pooled sensitivity (B) pooled specificity (C) pooled PLR (D) pooled NLR (E) pooled DOR (F) AUC.

A


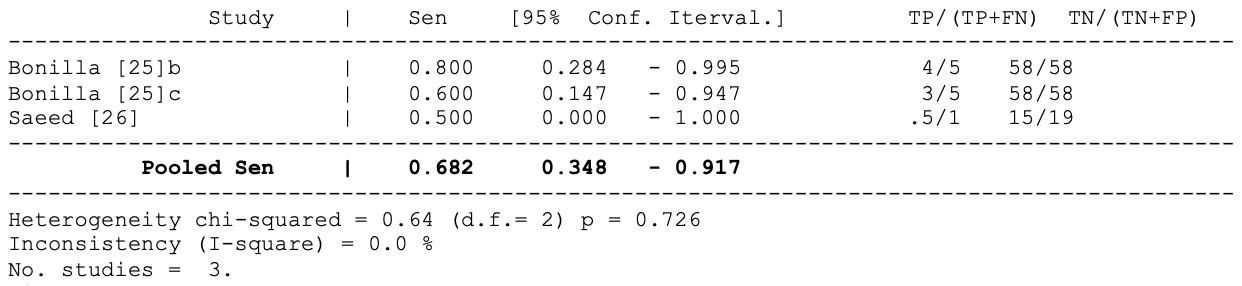


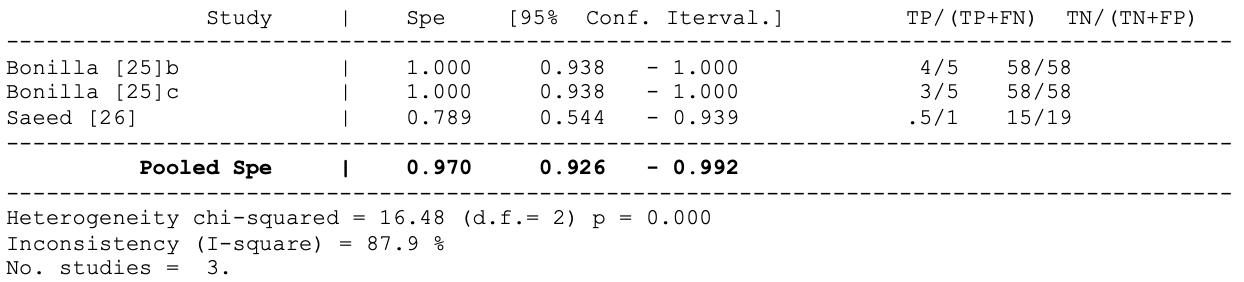


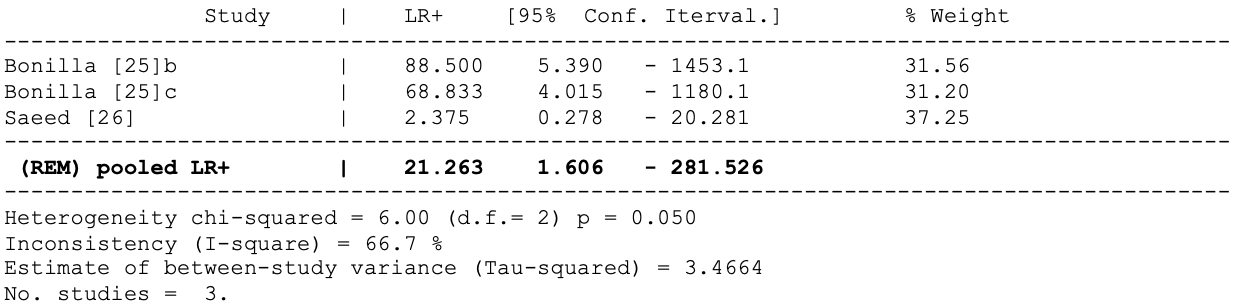


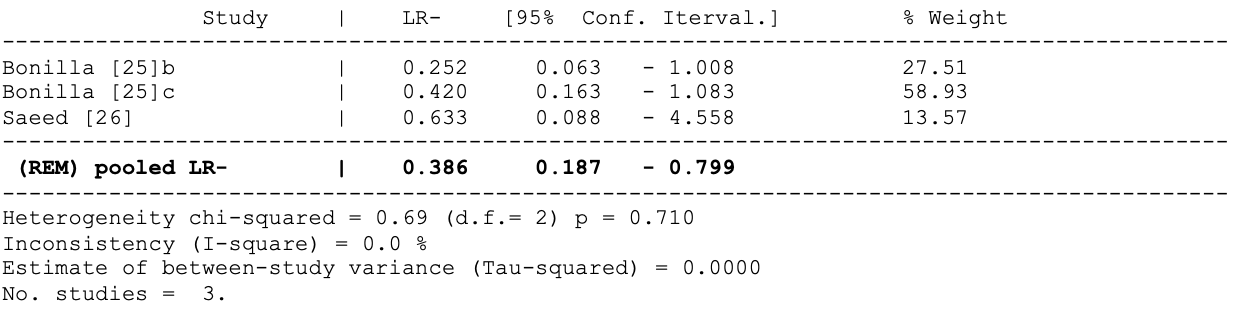


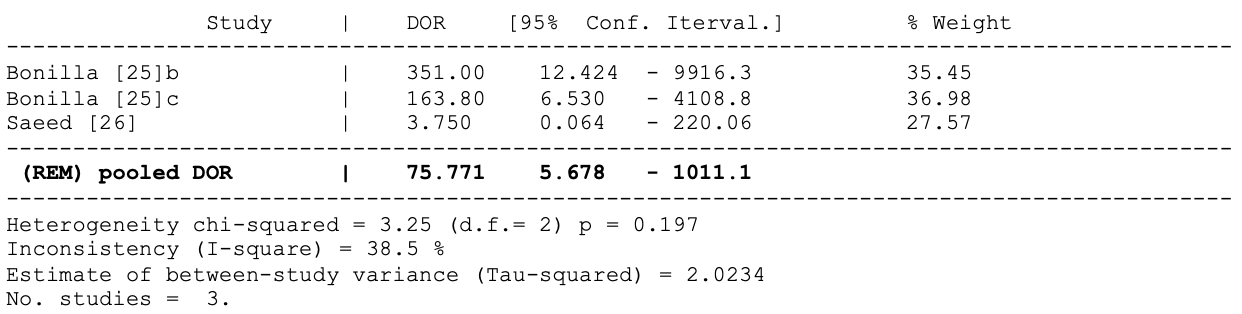


B

C

D

E


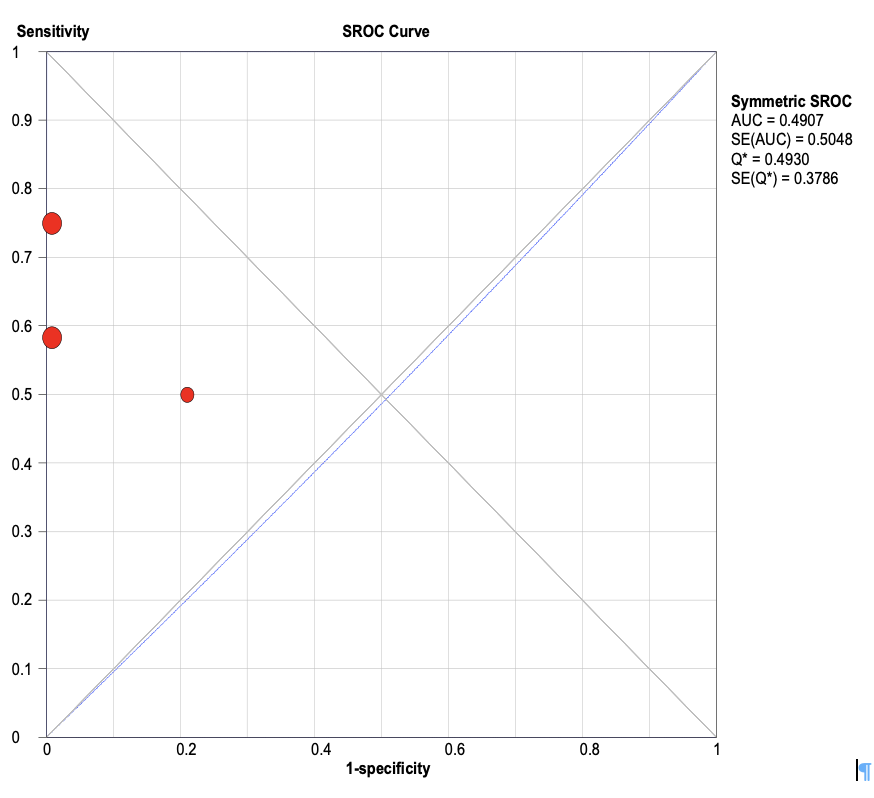


F

**Supplementary Fig. 7 Pooled summary estimates of RT-PCR for MSSA** detection. (A) pooled sensitivity (B) pooled specificity (C) pooled PLR (D) pooled NLR (E) pooled DOR (F) AUC. It should be noted that Bonilla [25] comprises three datasets that have been designated as Bonilla [25]a, Bonilla [25]b, and Bonilla [25]c to distinguish them. Bonilla [25]b and Bonilla [25]c compares the sensitivity/specificity of LightCycler PCR and TaqMan PCR to microbiological culture, respectively.

A


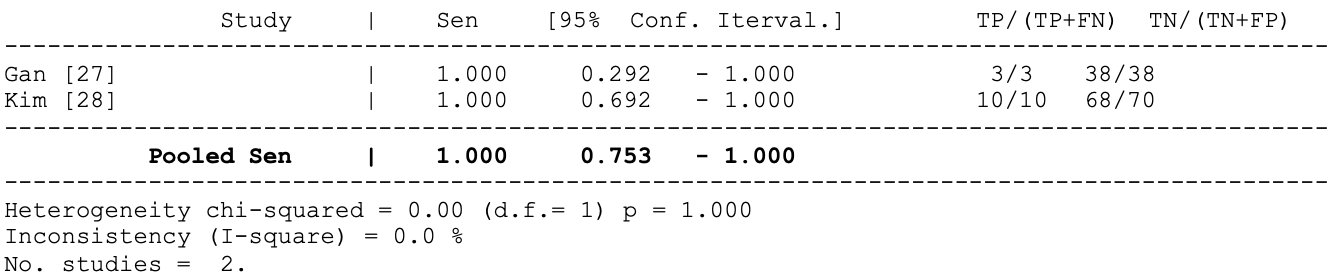


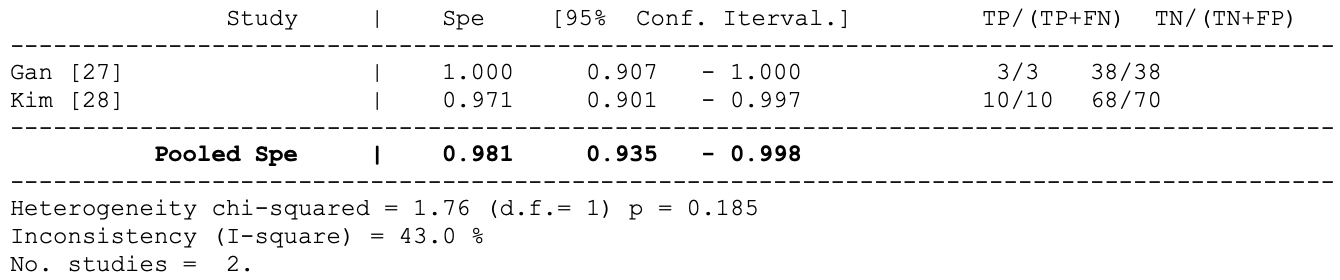


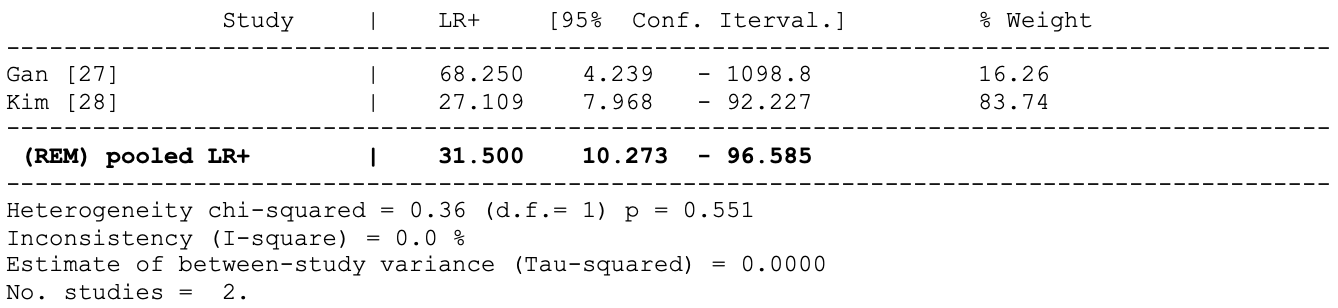


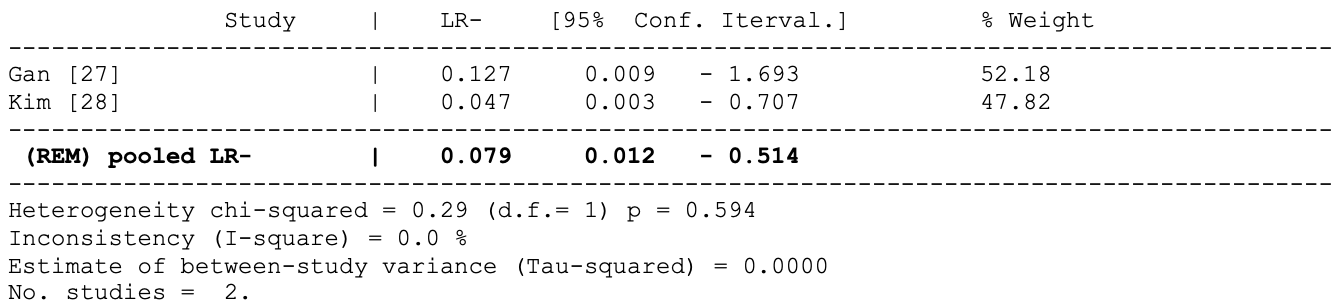


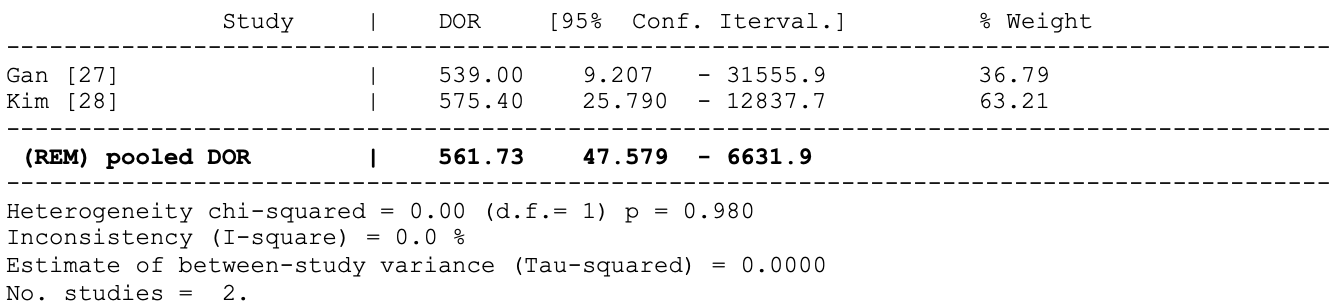


B

C

D

E

**Supplementary Fig. 8 Pooled summary estimates of mPCR for MSSA** detection. (A) pooled sensitivity (B) pooled specificity (C) pooled PLR (D) pooled NLR (E) pooled DOR. It should be noted that AUC curves generated using meta-disc require at least three studies.

A


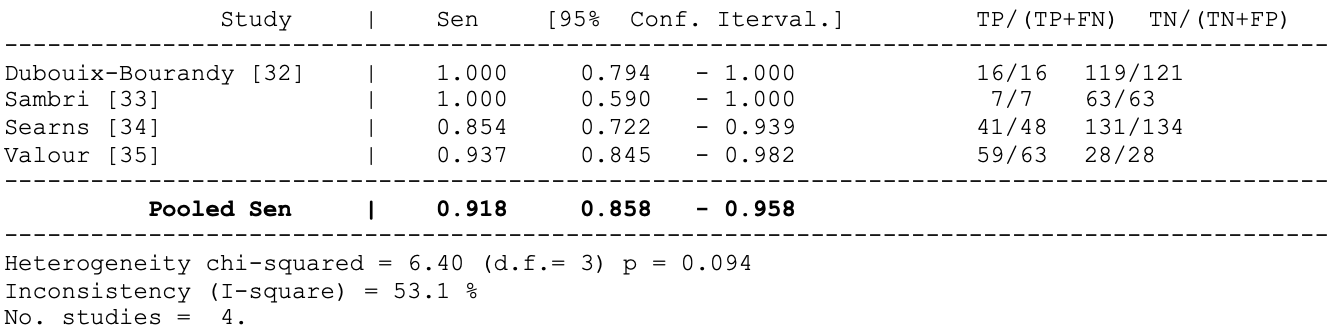


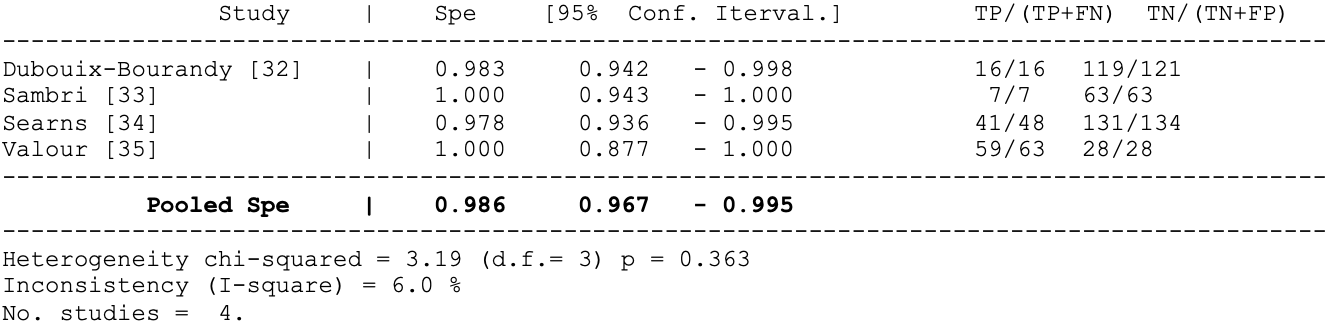


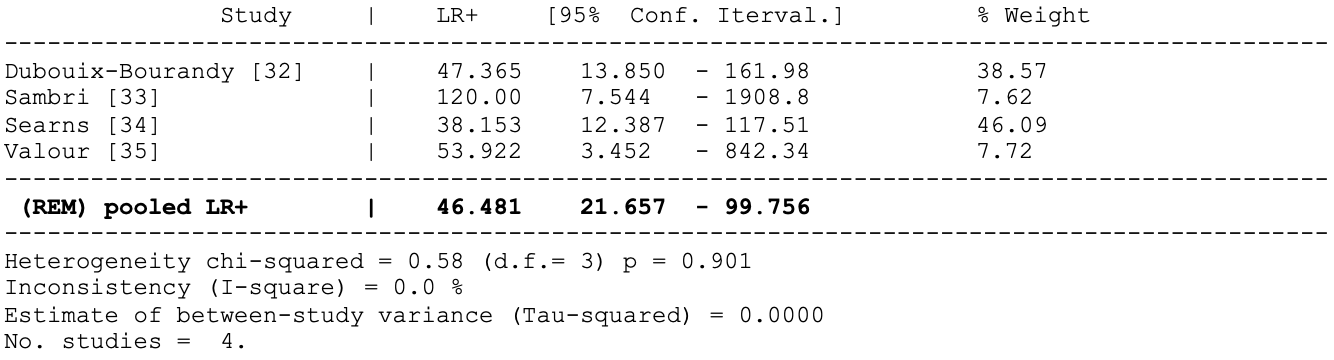


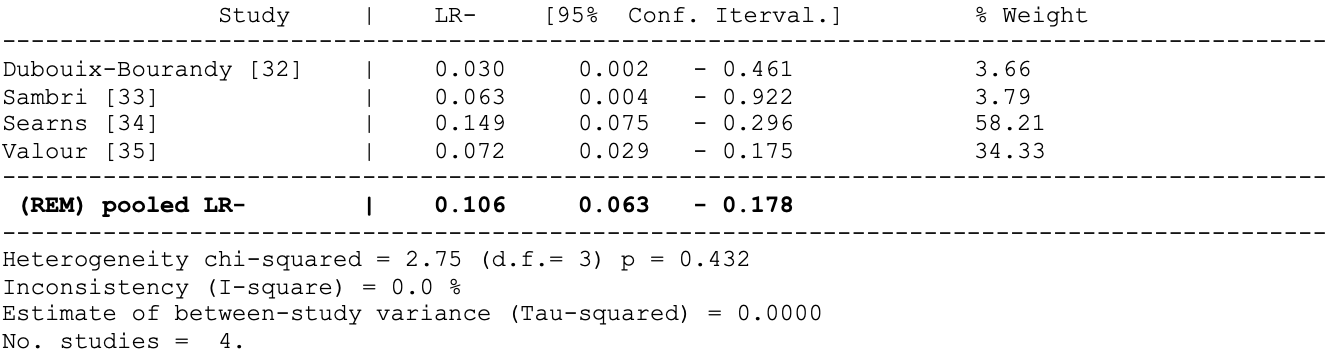


B

C

D


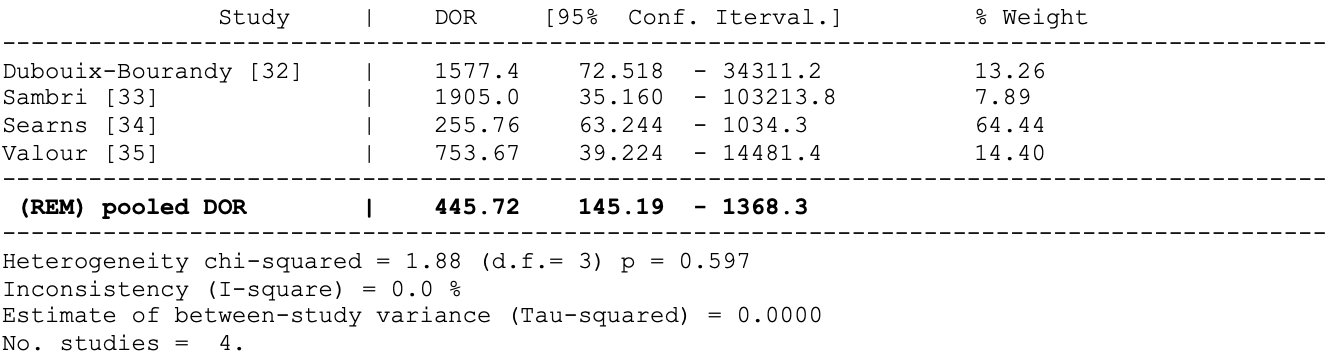


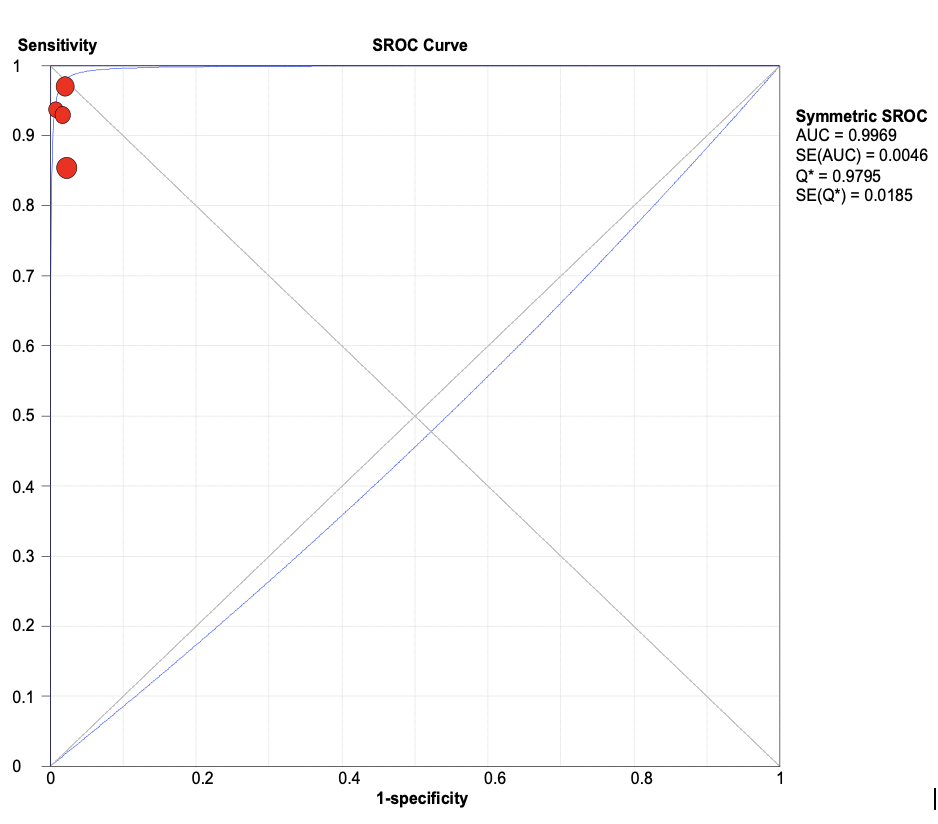


E

F

**Supplementary Fig. 9 Pooled summary estimates of Xpert for MSSA** detection. (A) pooled sensitivity (B) pooled specificity (C) pooled PLR (D) pooled NLR (E) pooled DOR.

A


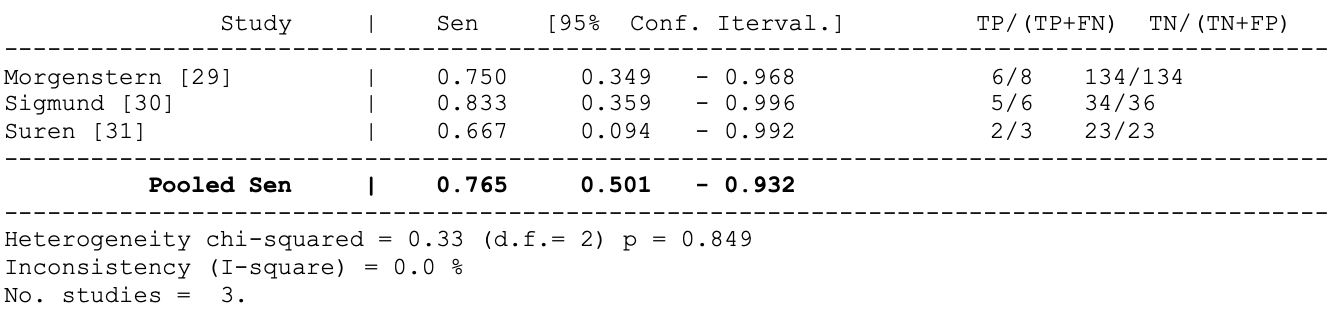


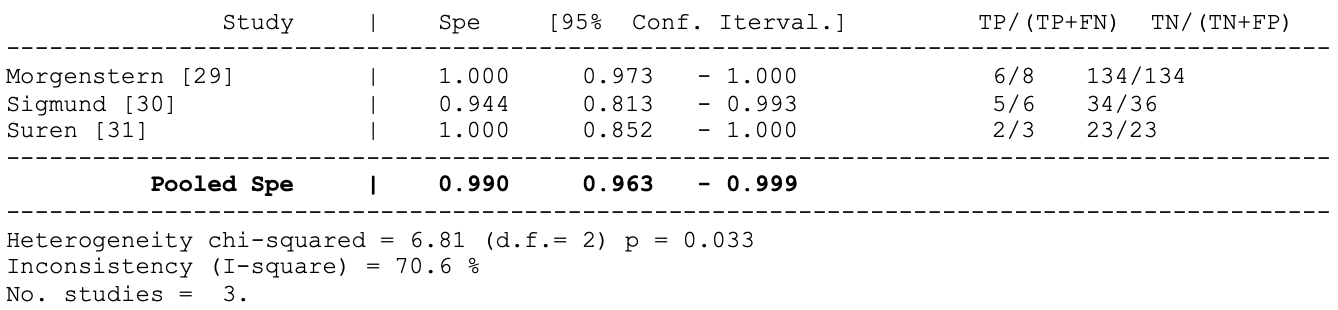


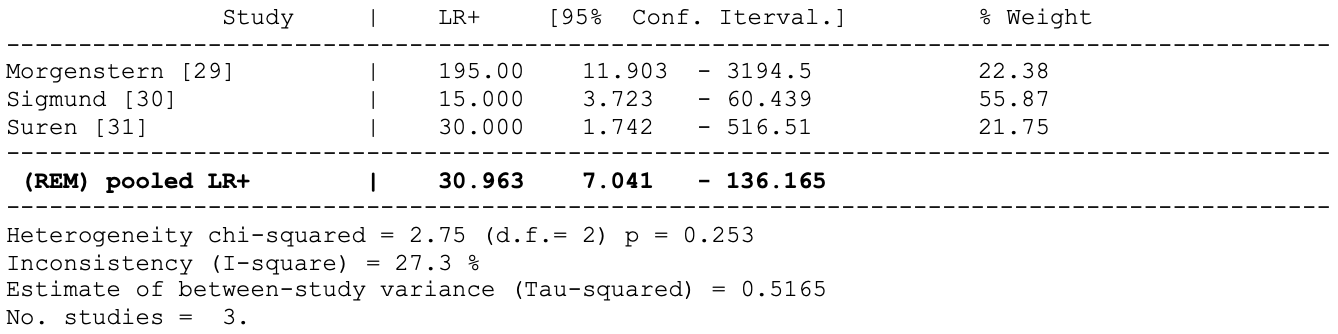


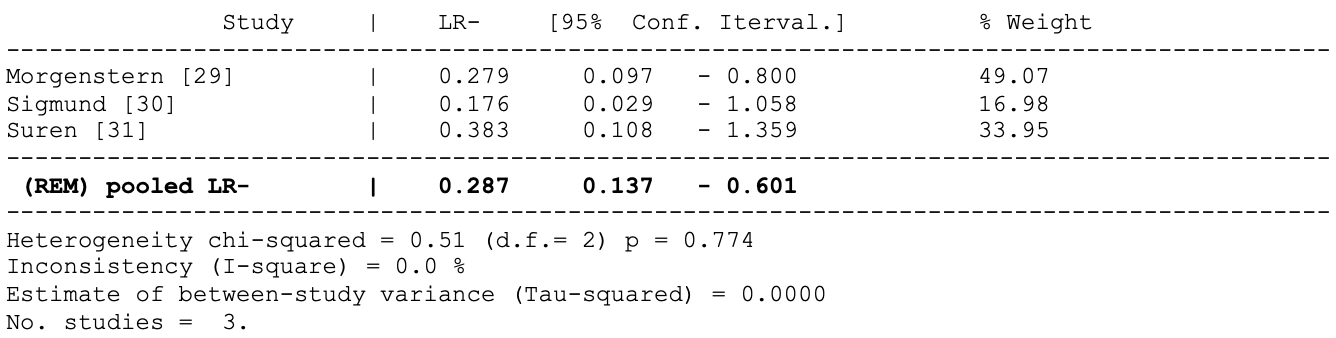


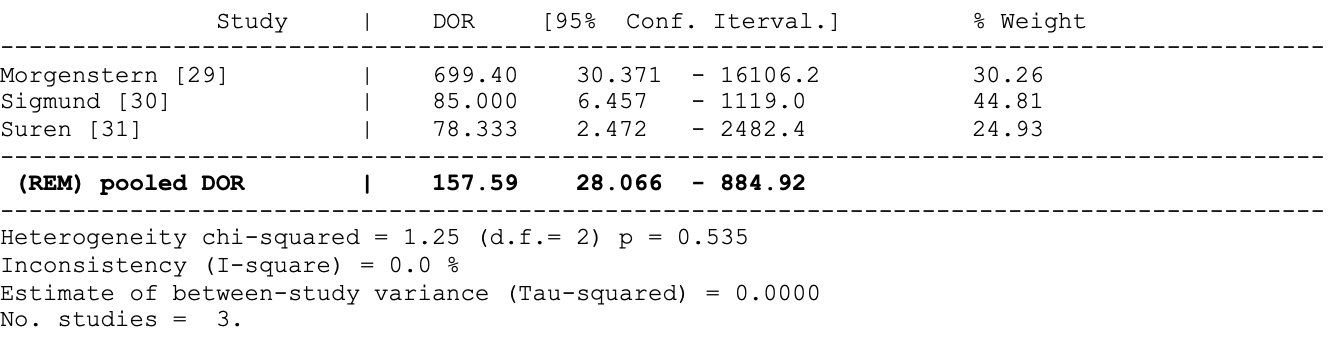


B

C

D

E


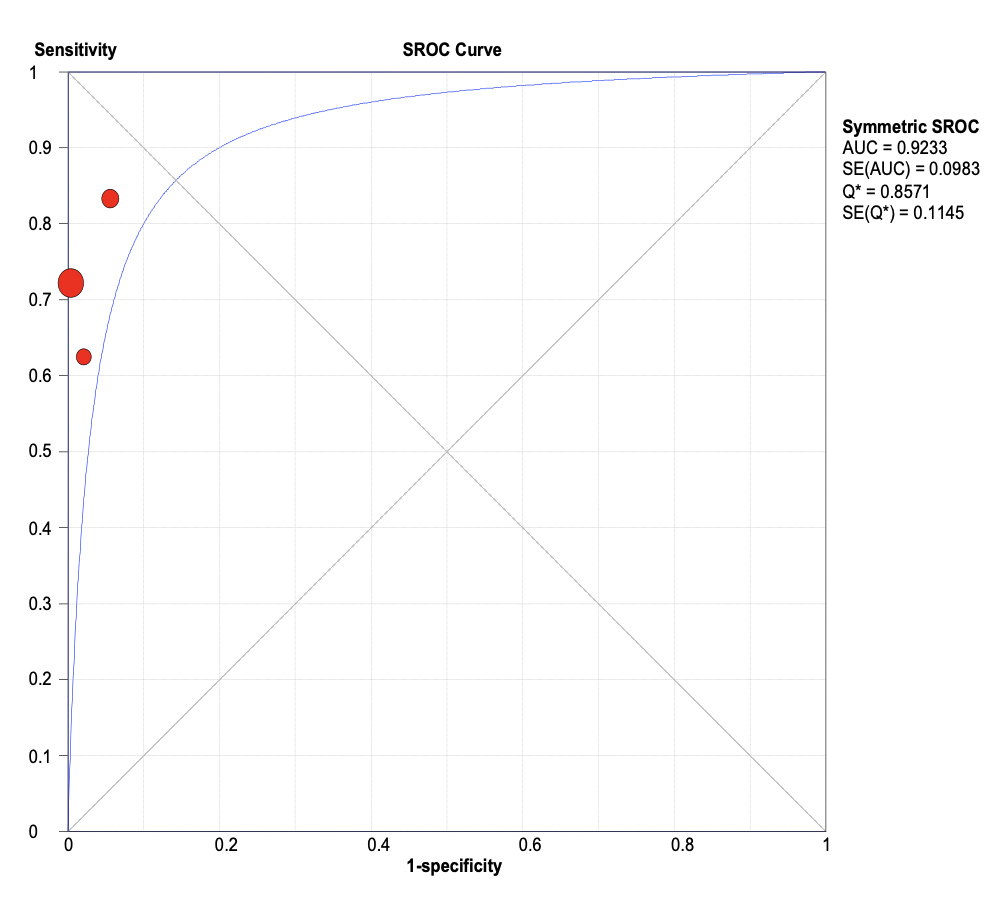


F

**Supplementary Fig. 10 Pooled summary estimates of mPCR-UITI for MSSA** detection. (A) pooled sensitivity (B) pooled specificity (C) pooled PLR (D) pooled NLR (E) pooled DOR (F) AUC

A


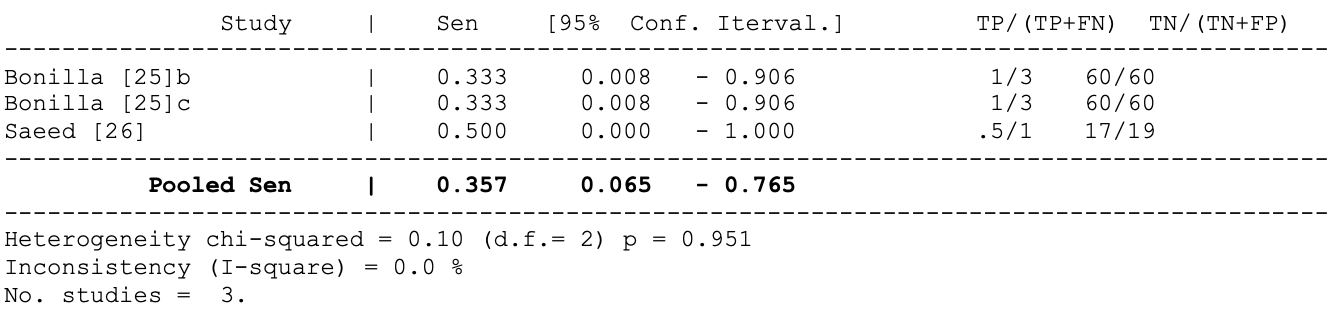


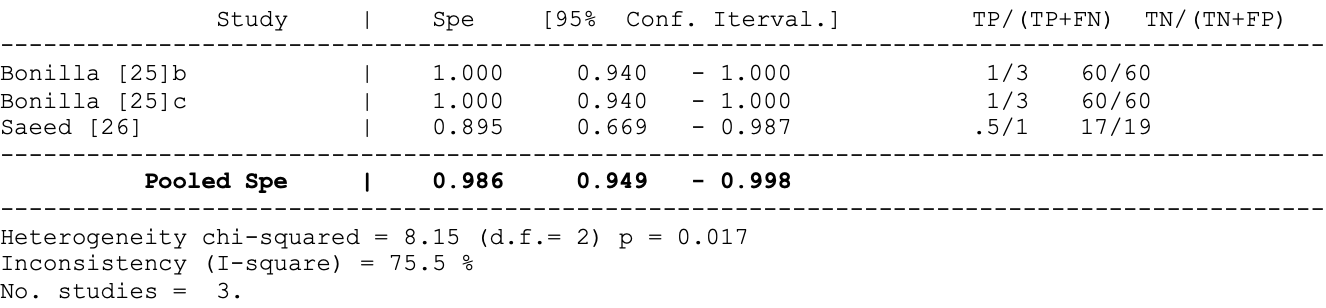


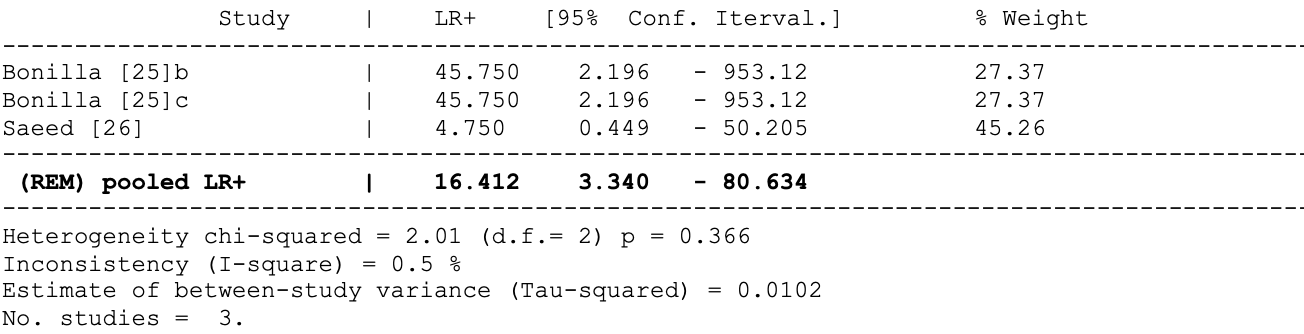


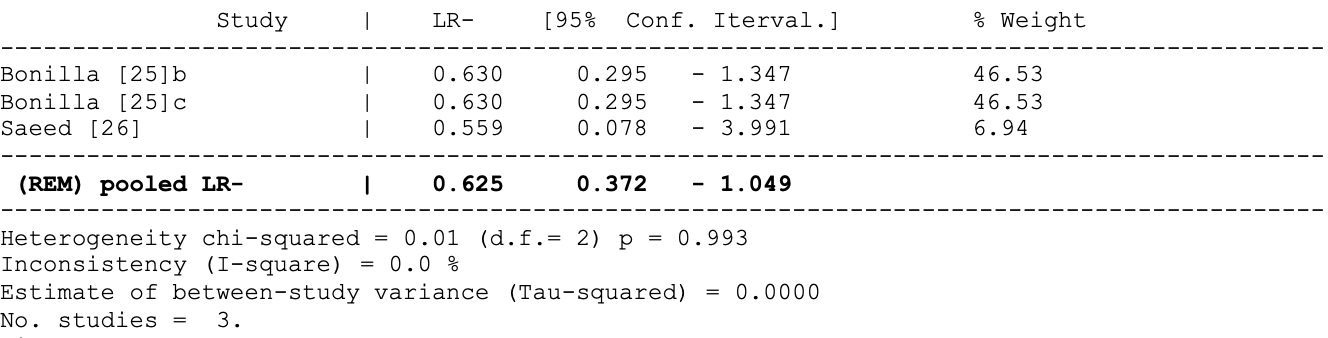


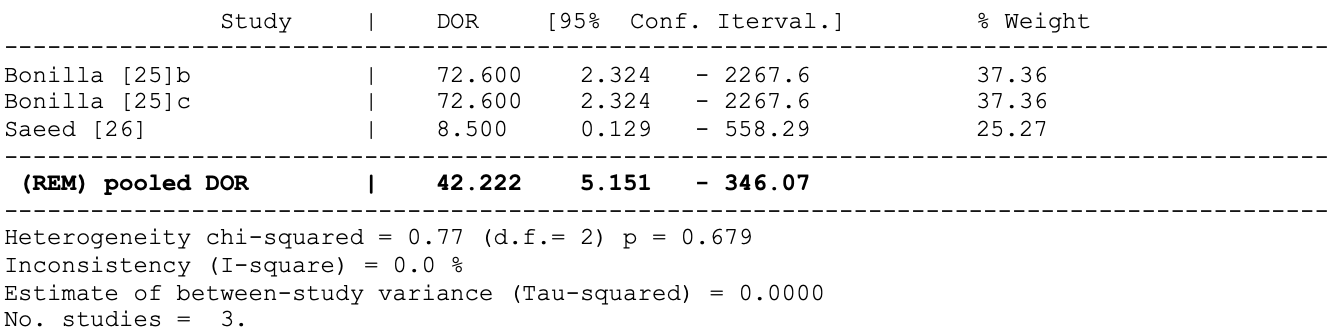


B

C

D

E


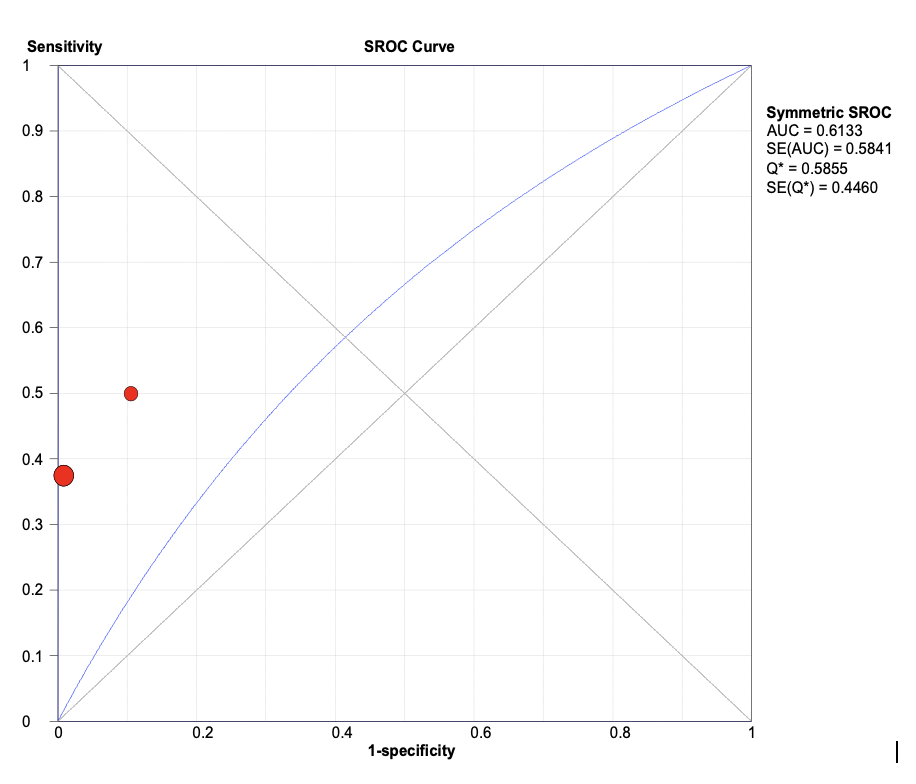


F

**Supplementary Fig. 11 Pooled summary estimates of RT-PCR for MRSA** detection. (A) pooled sensitivity (B) pooled specificity (C) pooled PLR (D) pooled NLR (E) pooled DOR. It should be noted that some of the overlapping numbers in AUC may be obscured.

A


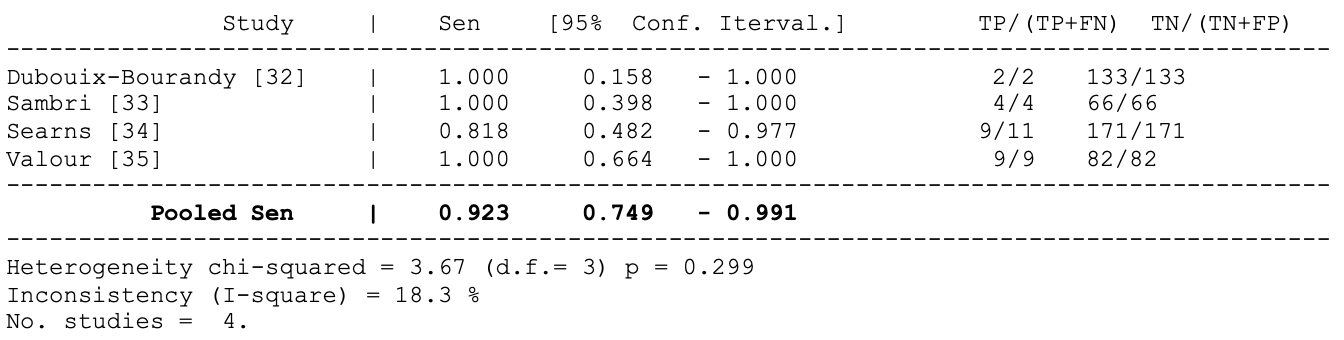


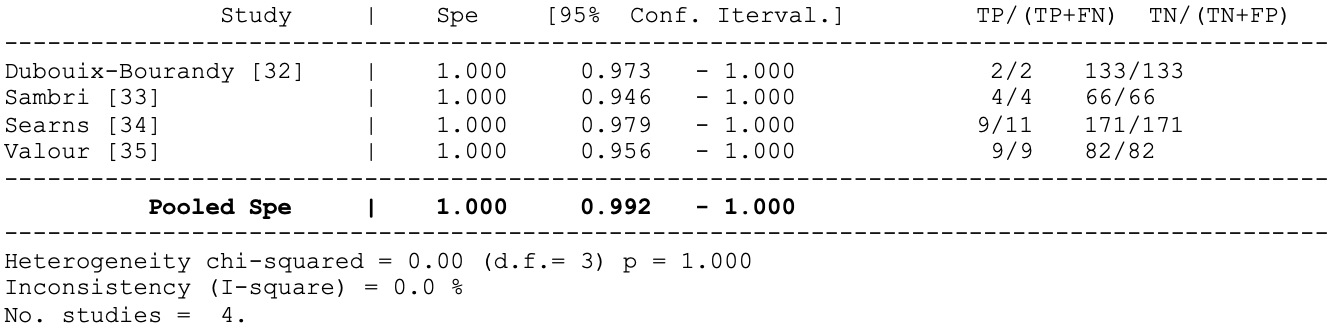


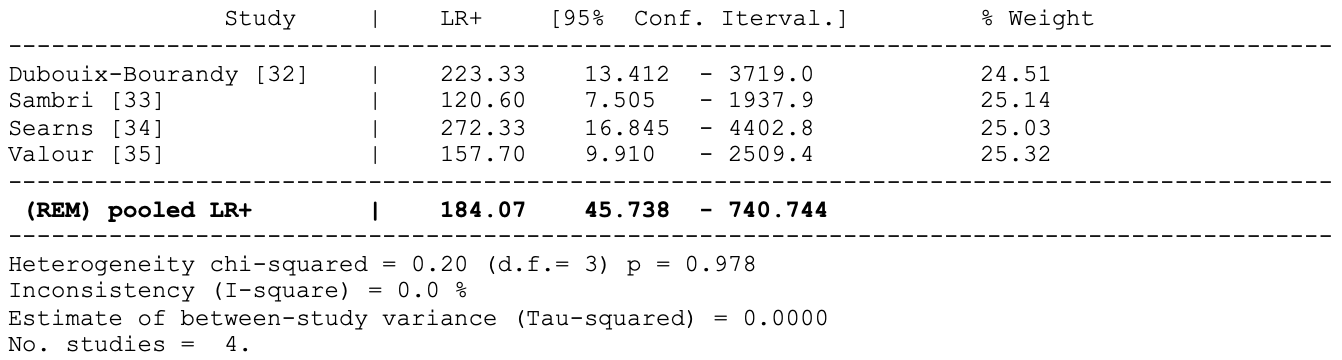


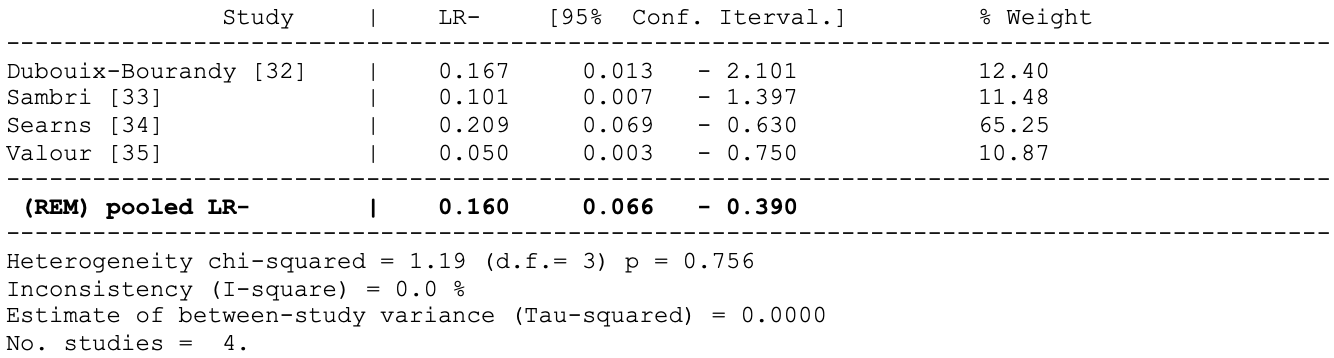


B

v

C

v

D

v


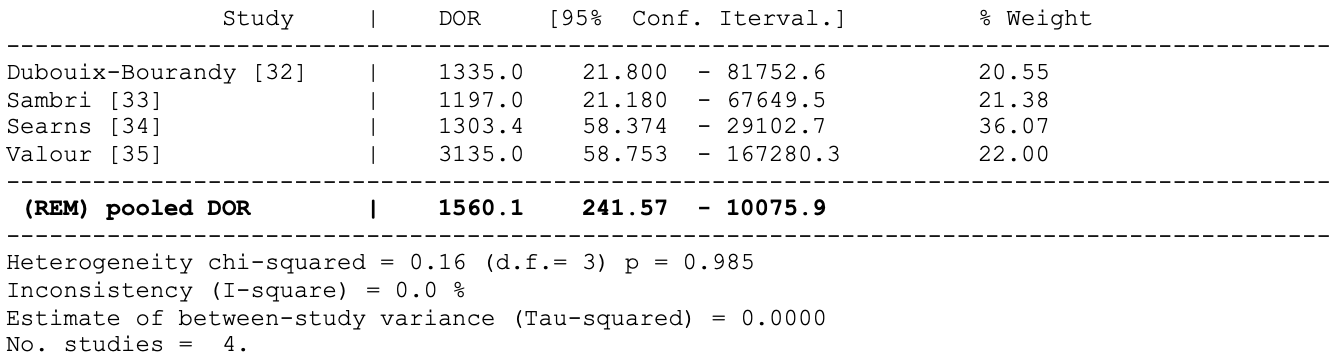


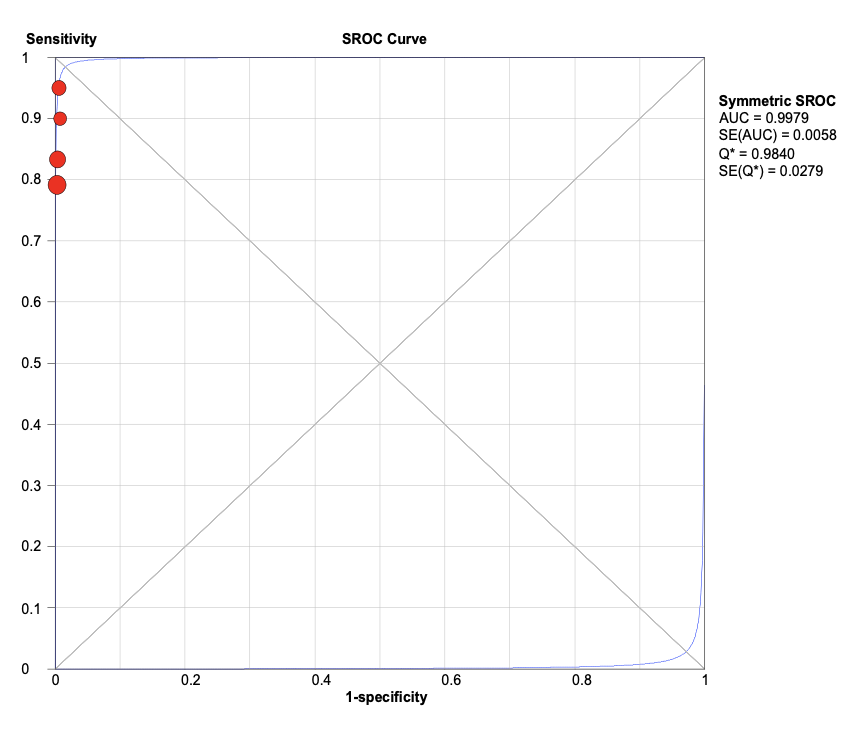


E

v

F

v

**Supplementary Fig. 12 Pooled summary estimates of commercial NAAT (Xpert) for MRSA** detection. (A) pooled sensitivity (B) pooled specificity (C) pooled PLR (D) pooled NLR (E) pooled DOR. Please note that all studies included in commercial tests for MRSA detection are the same as those of xpert detection.

A


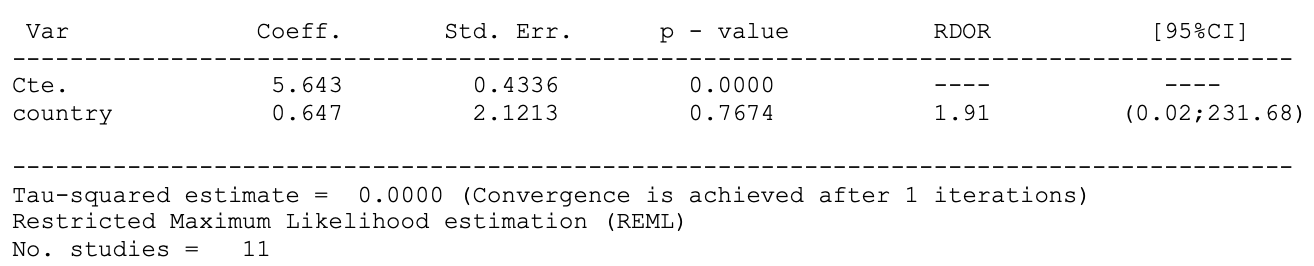


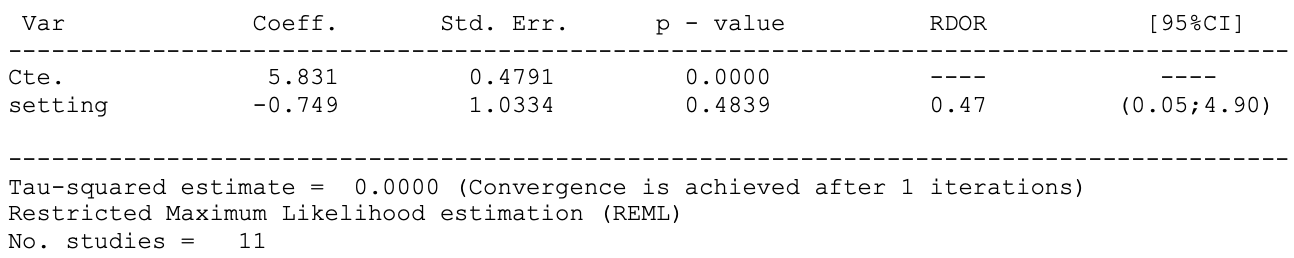


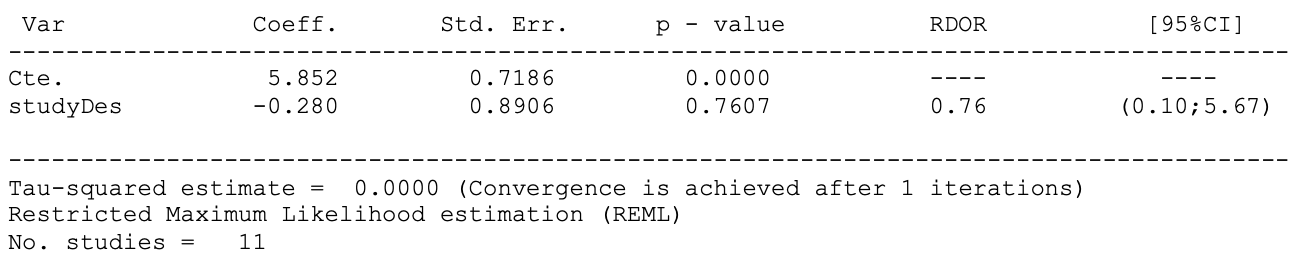


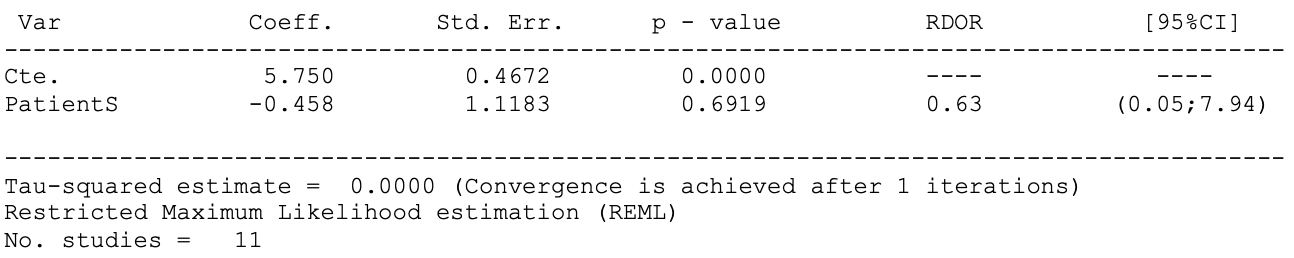


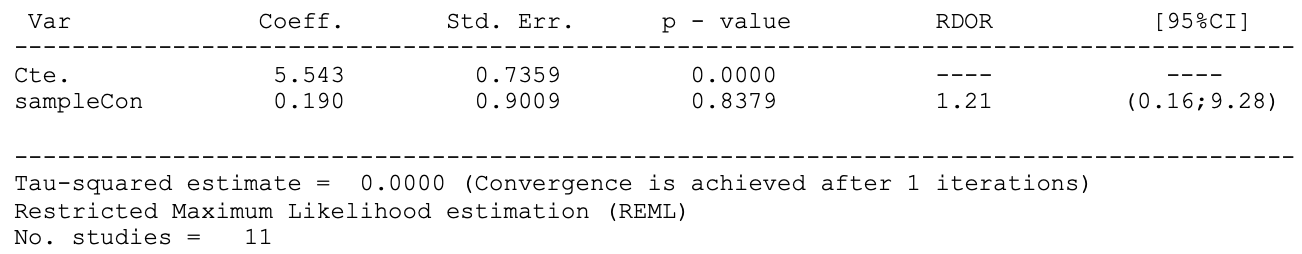


B

C

D

E

**Supplementary Fig. 13 Meta-regression analysis on pre-specified subgroups.** (A) country status (B) setting (C) study design (D) patient selection (E) sample condition





**Supplementary Fig. 14** Deek’s funnel plot asymmetry test for publication bias. STATA 16 was used to generate the graph. The numbers 1–22 represent the datasets used in our research for the diagnosis of MSSA and MRSA. Note that because some of the datasets come from the same studies, the overlapping of numbers may not be displayed.

**Table S1** Details of all the publications that were deemed relevant, and hence, fully screened by the first two authors (*n* = 170). Reasons why studies have been excluded are given (*n* = 159). Studies included in our meta-analysis are shaded with grey color (*n* = 11).

| First author [reference] | Inclusion (I)  /Exclusion (E) | Reasons |
| --- | --- | --- |
| Aamot (1) | E | 2×2 contingency table cannot be obtained |
| Abdel-Haq (2) | E | Characterization of isolated strains |
| Ako-Nai (3) | E | Characterization of isolated strains |
| Aksu (4) | E | Genotyping of isolated strains |
| Alli (5) | E | No sensitivity and specificity |
| Arikan (6) | E | Characterization of isolated strains |
| Askar (7) | E | 2×2 contingency table cannot be obtained |
| Ataee (8) | E | Evaluate presence of enterotoxin C in synovial fluid by ELISA and PCR gene amplification |
| Atala (9) | E | No sensitivity and specificity |
| Aung (10) | E | No sensitivity and specificity |
| Badiou (11) | E | Detection of PVL production in clinical specimens by immunoassays |
| Barraud (12) | E | Case reports |
| Bémer (13) | E | Diagnosis of PJI via serological tests |
| Bergin (14) | E | 2×2 contingency table cannot be obtained |
| Bhattacharya (15) | E | Bacterial isolation from surgical site infections |
| Biendo (16) | E | 2×2 contingency table cannot be obtained |
| Bonilla (17) | I |  |
| Borde (18) | E | 2×2 contingency table cannot be obtained |
| Bouchami (19) | E | Characterization of isolated strains |
| Bouras (20) | E | Characterization of isolated strains |
| Brakstad (21) | E | Verification of method using samples from wound infection |
| Breznicky (22) | E | No sensitivity and specificity |
| Carter (23) | E | 2×2 contingency table cannot be obtained |
| Cazanave (24) | E | 2×2 contingency table cannot be obtained |
| Chen (25) | E | Evaluates accuracy of electrochemical biosensor |
| Chen (26) | E | Characterization of isolated strains |
| Chiappini (27) | E | No sensitivity and specificity |
| Choe (28) | E | Not specific to MSSA and MRSA detection |
| Choe (29) | E | Not specific to MSSA and MRSA detection |
| Choi (30) | E | ﻿Compared culture with 16S rDNA PCR with sequencing |
| Chometon (31) | E | ﻿Compared culture with 16S rDNA PCR with sequencing |
| Cihanoglu (32) | E | No sensitivity and specificity |
| Coiffier (33) | E | 2×2 contingency table cannot be obtained |
| Crandall (34) | E | Characterization of isolated strains |
| Dande (35) | E | No sensitivity and specificity |
| Dash (36) | E | No sensitivity and specificity |
| Dastghey (37) | E | Insights into pathogenesis and resistance |
| Dave (38) | E | Selective screening of nasal swabs |
| Dayer (39) | E | No sensitivity and specificity |
| Daynes (40) | E | No sensitivity and specificity |
| De Man (41) | E | Correspondence |
| Deniz (42) | E | Not specific to BJI |
| Dubouix-Bourandy (43) | I |  |
| Dunyach-Remy (44) | E | 2×2 contingency table cannot be obtained |
| Ehrlich (45) | E | Editorial |
| Ehrlich (46) | E | No sensitivity and specificity |
| Eiamprapai (47) | E | Compared culture with 16S rDNA PCR with sequencing |
| Emberger (48) | E | Case reports |
| Eslami (49) | E | No molecular sensitivity and specificity |
| Fang (50) | E | 2×2 contingency table cannot be obtained |
| Ferroni (51) | E | No sensitivity and specificity |
| Fihman (52) | E | Pathogen identification via 16S rDNA and sequencing |
| Fuursted (53) | E | Compared culture with 16S rDNA PCR with sequencing |
| Gan (54) | I |  |
| Gasbarrini (55) | E | No sensitivity and specificity |
| Gomes (56) | E | No sensitivity and specificity |
| Gomez (57) | E | Compared culture with 16S rDNA PCR with sequencing |
| Gonzalez (58) | E | Case reports |
| Grif (59) | E | Compared culture with 16S rDNA PCR with sequencing |
| Gu (60) | E | Characterize of isolated strains |
| Gu (61) | E | Demonstrate the utility of mNGS in expanding the scope of conventional diagnostic testing to multiple body fluid types |
| Guarner (62) | E | No sensitivity and specificity |
| Gubbay (63) | E | No sensitivity and specificity |
| Gutierrez (64) | E | No sensitivity and specificity |
| Haag (65) | E | Compared culture with 16S rDNA PCR with sequencing |
| Harro (66) | E | Evaluation of antibody-based assays against *S. aureus* biofilm upregulated antigens |
| Helal (67) | E | Confirmation of positive *A. baumannii* isolates in surgical site infections (SSI) |
| Henares (68) | E | 2×2 contingency table cannot be obtained |
| Hinić (69) | E | 2×2 contingency table cannot be obtained |
| Ilharreborde (70) | E | ﻿Evaluation of a new real-time PCR method specific to *K. kingae* detection |
| Jacovides (71) | E | Compared culture with IBIS-based biosensor system |
| Jacquier (72) | E | Compared culture with 16S rDNA PCR with sequencing |
| Jahoda (73) | E | No sensitivity and specificity |
| Jain (74) | E | Characterization of isolated strains |
| Jeon (75) | E | No sensitivity and specificity |
| Jiang (76) | E | No sensitivity and specificity |
| Juchler (77) | E | No sensitivity and specificity |
| Kalogianni (78) | E | Detection of pathogens in a dry reagent-based dipstick format |
| Karbysheva (79) | E | D-lactate biomarker determination for diagnosis of PJI |
| Kathju (80) | E | Development of a method |
| Katsarou (81) | E | Evaluation of toxin genes in selected strains |
| Kawamura (82) | E | 2×2 contingency table cannot be obtained |
| Kechrid (83) | E | Characterization of isolated strains |
| Khosravi (84) | E | Determine the prevalence of toxin genes in staphylococcal strains |
| Kim (85) | E | Prescreening of staphylococcal strains using nasal swab |
| Kim (86) | I |  |
| Kobayashi (87) | E | 2×2 contingency table cannot be obtained |
| Kobayashi (88) | E | Case reports; developed for MRS detection |
| Kobayashi (89) | E | 2×2 contingency table cannot be obtained |
| Kourbatova (90) | E | No sensitivity and specificity |
| Lallemand (91) | E | Compared culture with 16S rDNA PCR with sequencing |
| Lane (92) | E | Evaluation of molecular tools in confirmed strains |
| Levy (93) | E | No sensitivity and specificity |
| Ley (94) | E | No sensitivity and specificity |
| Lourtet-Hascoëtt (95) | E | 2×2 contingency table cannot be obtained |
| Mariani (96) | E | Development of a method |
| Mariani (97) | E | Evaluation of molecular method in animal model |
| Mariaux (98) | E | No sensitivity and specificity |
| McCaskill (99) | E | Characterization of isolated strains |
| Melendez (100) | E | 2×2 contingency table cannot be obtained |
| Metso (101) | E | 2×2 contingency table cannot be obtained |
| Minegishi (102) | E | Compared culture with lateral flow-based assay |
| Miyamae (103) | E | 2×2 contingency table cannot be obtained |
| Moojen (104) | E | Hybridization based method for identification of pathogens |
| Morel (105) | E | 2×2 contingency table cannot be obtained |
| Morgenstern (106) | I |  |
| Morgenstern (107) | E | 2×2 contingency table cannot be obtained |
| Muñoz-Gamito (108) | E | Characterization of isolated strains |
| Mussa (109) | E | No sensitivity and specificity |
| Ong (110) | E | Antimicrobial resistance determination in isolated strains |
| Palmer (111) | E | Compared culture with PCR–ESI–TOF–MS |
| Papan (112) | E | 2×2 contingency table cannot be obtained |
| Parcell (113) | E | Prescreening of samples using nasal swab |
| Pardo (114) | E | Characterization of isolated strains |
| Portillo (115) | E | 2×2 contingency table cannot be obtained |
| Post (116) | E | Characterization of isolated strains |
| Premru (117) | E | Characterization of isolated strains |
| Rahman (118) | E | No sensitivity and specificity |
| Reissier (119) | E | Evaluation of mPCR for infected chronic leg ulcer |
| Renz (120) | E | 2×2 contingency table cannot be obtained |
| Renz (121) | E | 2×2 contingency table cannot be obtained |
| Rincón (122) | E | Characterization of isolated strains |
| Rosey (123) | E | Compared culture with 16 S rDNA and sequencing |
| Rupp (124) | E | No sensitivity and specificity |
| Russell (125) | E | Meta-analysis, no sensitivity and specificity |
| Ryu (126) | E | 2×2 contingency table cannot be obtained |
| Saeed (127) | I |  |
| Sambri (128) | I |  |
| Sampedro (129) | E | 2×2 contingency table cannot be obtained |
| San Juan (130) | E | Characterization of *S. aureus* nasal isolates |
| Sancho-Tello (131) | E | 2×2 contingency table cannot be obtained |
| Sarma (132) | E | No sensitivity and specificity |
| Sauer (133) | E | No sensitivity and specificity |
| Saviauk (134) | E | Evaluation of accuracy via IMS system |
| Sdougkos (135) | E | Characterization of isolated strains |
| Searns (136) | I |  |
| Sebastian (137) | E | Compared culture with 16S rDNA and sequencing |
| Shetty (138) | E | 2×2 contingency table cannot be obtained |
| Sigmund (139) | I |  |
| Sigmund (140) | E | Detection of resistant genes from obtained isolates |
| Sigmund (141) | E | 2×2 contingency table cannot be obtained |
| Stoodley (142) | E | Case reports; confirmation via biosensor-based system |
| Stuhlmeier (143) | E | No sensitivity and specificity |
| Subhadharsini (144) | E | No sensitivity and specificity |
| Suren (145) | I |  |
| Szczȩsny (146) | E | 2×2 contingency table cannot be obtained |
| Tarkin (147) | E | No distinction made among MRSA and MRConS |
| Tenover (148) | E | Letter to the editor |
| Titécat (149) | E | 2×2 contingency table cannot be obtained |
| Titécat (150) | E | Letter to the editor |
| Tsai (151) | E | Isolates were obtained from necrotizing fasciitis |
| Tsuru (152) | E | Evaluates centrifugation effect on gene concentration |
| Uçkay (153) | E | Letter to the editor |
| Valour (154) | I |  |
| Vandercam (155) | E | 2×2 contingency table cannot be obtained |
| Vasoo (156) | E | Letter to the editor |
| Villa (157) | E | 2×2 contingency table cannot be obtained |
| Wang (158) | E | Compared culture with 16 S rDNA and sequencing |
| Wang (159) | E | Development of LAMP method for food-borne *S. aureus* analysis |
| Wang (160) | E | Development of a method |
| Wang (161) | E | Characterization of isolated strains |
| Wang (162) | E | No sensitivity and specificity |
| Wang (163) | E | Case reports |
| Yang (164) | E | compared culture with 16S rRNA and sequencing |
| Yang (165) | E | 2×2 contingency table cannot be obtained |
| Yang (166) | E | 2×2 contingency table cannot be obtained |
| Zegaer (167) | E | Evaluation of molecular tools with confirmed strains |
| Zhao (168) | E | Comparison of mNGS vs microbiological culture |
| Zhu (169) | E | Evaluation of positive blood culture |
| Zhu (170) | E | Evaluation of positive blood culture |

*Abbreviations:* MRSA, methicillin-resistant Staphylococcus aureus; MSSA, methicillin-sensitive Staphylococcus aureus; ConS, coagulase negative staphylococci; SSI, surgical site infections; IMS, ion mobility spectrum; LAMP, loop-mediated isothermal amplification; mNGS, metagenomic next-generation sequencing; spp., species; PCR, polymerase chain reaction; PJI, prosthetic joint infection; BJI, bone and joint infection; AST, antibiotic sensitivity test; ELISA, enzyme-linked immunosorbent assay.

**References**

1. Aamot HV, Johnsen BO, Skråmm I. Rapid diagnostics of orthopedic implant-associated infections using Unyvero ITI implant and tissue infection application is not optimal for Staphylococcus species identification. BMC Research Notes. 2019;12(1).

2. Abdel-Haq N, Al-Tatari H, Chearskul P, Salimnia H, Asmar BI, Fairfax MR, et al. Methicillin-resistant staphylococcus aureus (MRSA) in hospitalized children: Correlation of molecular analysis with clinical presentation and antibiotic susceptibility testing (ABST) results. European Journal of Clinical Microbiology and Infectious Diseases. 2009;28(5):547-51.

3. Ako-Nai KA, Attah OT, Akinyoola AL. Molecular characterization and multiresistance of bacteria isolates among surgical site infection patients. Annals of Tropical Medicine and Public Health. 2019;18(Special issue).

4. Aksu B, Yaǧci A, Ilki A, Söyletir G. Molecular epidemiology of MRSA isolates in the intensive care unit during a 4-year-period. Marmara Med J. 2002;15(3):151-4.

5. Alli OA, Ogbolu DO, Shittu AO, Okorie AN, Akinola JO, Daniel JB. Association of virulence genes with mecA gene in Staphylococcus aureus isolates from Tertiary Hospitals in Nigeria. Indian J Pathol Microbiol. 2015;58(4):464-71.

6. Arikan K, Karadag-Oncel E, Aycan AE, Yuksekkaya S, Sancak B, Ceyhan M. Epidemiologic and Molecular Characteristics of Staphylococcus aureus Strains Isolated From Hospitalized Pediatric Patients. Pediatr Infect Dis J. 2020;39(11):1002-6.

7. Askar M, Sajid M, Nassif Y, Ashraf W, Scammell B, Bayston R. Propidium monoazide-polymerase chain reaction for detection of residual periprosthetic joint infection in two-stage revision. Mol Biol Rep. 2019;46(6):6463-70.

8. Ataee RA, Ataee MH, Alishiri GH, Esmaeili D. Staphylococcal enterotoxin C in synovial fluid of patients with rheumatoid arthritis. Iran Red Crescent MedJ. 2014;16(10):1-6.

9. Atala ML. Bacteriological and molecular study on S. Aureus bacteria. Res J Pharm Technol. 2021;14(3):1380-4.

10. Aung MS, Zi H, Nwe KM, Maw WW, Aung MT, Min WW, et al. Drug resistance and genetic characteristics of clinical isolates of staphylococci in Myanmar: High prevalence of PVL among methicillin-susceptible Staphylococcus aureus belonging to various sequence types. New Microbes New Infect. 2016;10:58-65.

11. Badiou C, Dumitrescu O, George N, Forbes ARN, Drougka E, Chan KS, et al. Rapid Detection of Staphylococcus aureus Panton-Valentine Leukocidin in Clinical Specimens by Enzyme-Linked Immunosorbent Assay and Immunochromatographic Tests. Journal of Clinical Microbiology. 2010;48(4):1384-90.

12. Barraud O, Laurent F, François B, Bes M, Vignon P, Ploy MC. Severe human bone infection due to methicillin-resistant staphylococcus aureus carrying the novel mecC variant. Journal of Antimicrobial Chemotherapy. 2013;68(12):2949-50.

13. Bémer P, Bourigault C, Jolivet-Gougeon A, Plouzeau-Jayle C, Lemarie C, Chenouard R, et al. Assessment of a Multiplex Serological Test for the Diagnosis of Prosthetic Joint Infection: a Prospective Multicentre Study. J Bone Jt Infect. 2020;5(2):89-95.

14. Bergin PF, Doppelt JD, Hamilton WG, Mirick GE, Jones AE, Sritulanondha S, et al. Detection of periprosthetic infections with use of ribosomal RNA-based polymerase chain reaction. J Bone Joint Surg Am. 2010;92(3):654-63.

15. Bhattacharya S, Pal K, Jain S, Chatterjee SS, Konar J. Surgical site infection by methicillin resistant staphylococcus aureus– On decline? Journal of Clinical and Diagnostic Research. 2016;10(9):DC32-DC6.

16. Biendo M, Mammeri H, Pluquet E, Guillon H, Rousseau F, Canarelli B, et al. Value of Xpert MRSA/SA blood culture assay on the Gene Xpert® Dx System for rapid detection of Staphylococcus aureus and coagulase-negative staphylococci in patients with staphylococcal bacteremia. Diagnostic Microbiology and Infectious Disease. 2013;75(2):139-43.

17. Bonilla H, Kepley R, Pawlak J, Belian B, Raynor A, Saravolatz LD. Rapid diagnosis of septic arthritis using 16S rDNA PCR: A comparison of 3 methods. Diagnostic Microbiology and Infectious Disease. 2011;69(4):390-5.

18. Borde JP, Häcker GA, Guschl S, Serr A, Danner T, Hübner J, et al. Diagnosis of prosthetic joint infections using UMD-Universal Kit and the automated multiplex-PCR Unyvero i60 ITI® cartridge system: a pilot study. Infection. 2015;43(5):551-60.

19. Bouchami O, Achour W, Ben Hassen A. Typing of staphylococcal cassette chromosome mec encoding methicillin resistance in staphylococcus aureus strains isolated at the bone marrow transplant centre of Tunisia. Curr Microbiol. 2009;59(4):380-5.

20. Bouras D, Doudoulakakis A, Tsolia M, Vaki I, Giormezis N, Petropoulou N, et al. Staphylococcus aureus osteoarticular infections in children: an 8-year review of molecular microbiology, antibiotic resistance and clinical characteristics. J Med Microbiol. 2018;67(12):1753-60.

21. Brakstad OG, Aasbakk K, Maeland JA. Detection of Staphylococcus aureus by polymerase chain reaction amplification of the nuc gene. Journal of Clinical Microbiology. 1992;30(7):1654-60.

22. Breznicky J, Novak M. The most common etiological agents of prosthetic joint infections in orthopaedics. Med Glas. 2019;16(2):185-9.

23. Carter K, Doern C, Jo CH, Copley LAB. The Clinical Usefulness of Polymerase Chain Reaction as a Supplemental Diagnostic Tool in the Evaluation and the Treatment of Children With Septic Arthritis. J Pediatr Orthop. 2016;36(2):167-72.

24. Cazanave C, Greenwood-Quaintance KE, Hanssen AD, Karau MJ, Schmidt SM, Urena EOG, et al. Rapid Molecular Microbiologic Diagnosis of Prosthetic Joint Infection. Journal of Clinical Microbiology. 2013;51(7):2280-7.

25. Chen JY, Wei HX, Fang XY, Cai YQ, Zhang ZZ, Wang YQ, et al. A pragmatic eLCR for an ultrasensitive detection of methicillin-resistant Staphylococcus aureus in joint synovial fluid: superior to qPCR. Analyst. 2021;146(11):3500-9.

26. Chen P, Sun F, Feng W, Hong H, Li B, Song J. Pathogenic characteristics of Staphylococcus aureus isolates from arthroplasty infections. Int J Artif Organs. 2021;44(3):208-14.

27. Chiappini E, Camposampiero C, Lazzeri S, Indolfi G, De Martino M, Galli L. Epidemiology and management of acute haematogenous osteomyelitis in a tertiary paediatric center. Int J Environ Res Public Health. 2017;14(5).

28. Choe H, Aota Y, Kobayashi N, Nakamura Y, Wakayama Y, Inaba Y, et al. Rapid sensitive molecular diagnosis of pyogenic spinal infections using methicillin-resistant Staphylococcus-specific polymerase chain reaction and 16S ribosomal RNA gene-based universal polymerase chain reaction. Spine J. 2014;14(2):255-62.

29. Choe H, Inaba Y, Kobayashi N, Aoki C, Machida J, Nakamura N, et al. Use of real-time polymerase chain reaction for the diagnosis of infection and differentiation between gram-positive and gram-negative septic arthritis in children. J Pediatr Orthop. 2013;33(3):e28-e33.

30. Choi SH, Sung H, Kim SH, Lee SO, Lee SH, Kim YS, et al. Usefulness of a direct 16S rRNA gene PCR assay of percutaneous biopsies or aspirates for etiological diagnosis of vertebral osteomyelitis. Diagnostic Microbiology and Infectious Disease. 2014;78(1):75-8.

31. Chometon S, Benito Y, Chaker M, Boisset S, Ploton C, Bérard J, et al. Specific real-time polymerase chain reaction places Kingella kingae as the most common cause of osteoarticular infections in young children. Pediatr Infect Dis J. 2007;26(5):377-81.

32. Cihanoglu N, Adaleti R, Nakipoglu Y. Investigation of Fibronectin Binding Protein (FBP) and Panton Valentine Leukocidin (PVL) Viulance Factors in Clinical Methicillin Sensitive and Resistant Staphylococcus Aureus Strains. Clin Lab. 2019;65(1).

33. Coiffier G, David C, Gauthier P, Le Bars H, Guggenbuhl P, Jolivet-Gougeon A, et al. Broad-range 16 s rDNA PCR in synovial fluid does not improve the diagnostic performance of septic arthritis in native joints in adults: cross-sectional single-center study in 95 patients. Clin Rheumatol. 2019.

34. Crandall H, Kapusta A, Killpack J, Heyrend C, Nilsson K, Dickey M, et al. Clinical and molecular epidemiology of invasive Staphylococcus aureus infection in Utah children; continued dominance of MSSA over MRSA. PLoS ONE. 2020;15(9 September).

35. Dande A, Not LG, Bucs G, Kocsis B, Lorinczy D, Wiegand N. Efficacy of microbiological culturing in the diagnostics of joint and periprosthetic infections. Injury-International Journal of the Care of the Injured. 2021;52:S48-S52.

36. Dash N, Panigrahi D, Al Zarouni M, Yassin F, Al-Shamsi M. Incidence of community-acquired methicillin-resistant Staphylococcus aureus carrying Pantone-Valentine leucocidin gene at a referral hospital in United Arab Emirates. APMIS. 2014;122(4):341-6.

37. Dastghey S, Parvizi J, Shapiro IM, Hickok NJ, Otto M. Effect of biofilms on recalcitrance of staphylococcal joint infection to antibiotic treatment. Journal of Infectious Diseases. 2015;211(4):641-50.

38. Dave J, Jenkins PJ, Hardie A, Smith M, Gaston P, Gibb AP, et al. A selected screening programme was less effective in the detection of methicillin-resistant Staphylococcus aureus colonisation in an orthopaedic unit. International Orthopaedics. 2014;38(1):163-7.

39. Dayer R, Alzahrani MM, Saran N, Ouellet JA, Journeau P, Tabard-Fougère A, et al. Spinal infections in children: a multicentre retrospective study. Bone Joint J. 2018;100-b(4):542-8.

40. Daynes J, Roth MF, Zekaj M, Hudson I, Pearson C, Vaidya R. Adult Native Septic Arthritis in an Inner City Hospital: Effects on Length of Stay. Orthopedics. 2016;39(4):E674-E9.

41. De Man FHR, Graber P, Lüem M, Zimmerli W, Ochsner PE, Sendi P. Broad-range PCR in selected episodes of prosthetic joint infection. Infection. 2009;37(3):292-4.

42. Deniz NY, Bayram Y, Parlak M, Irden Ş, Güdücüoğlu H. The determination of meca gene presence in mrsa strains isolated from intensive care unit by conventional, automated and pcr method. Eastern Journal of Medicine. 2021;26(1):99-103.

43. Dubouix-Bourandy A, De Ladoucette A, Pietri V, Mehdi N, Benzaquen D, Guinand R, et al. Direct detection of Staphylococcus osteoarticular infections by use of xpert MRSA/SA SSTI real-time PCR. Journal of Clinical Microbiology. 2011;49(12):4225-30.

44. Dunyach-Remy C, Carrere C, Marchandin H, Schuldiner S, Guedj AM, Cellier N, et al. Performance of the automated multiplex PCR Unyvero implant and tissue infections system in the management of diabetic foot osteomyelitis. Future Microbiol. 2018;13(15):1669-81.

45. Ehrlich GD. Next-Generation Molecular Diagnostics Provide Evidence Suggestive of a Role for Nontraditional Bacterial Pathogens in Osteoarthritis of the Knee. Gent Test and Mol Biomarkers. 2016;20(12):719-20.

46. Ehrlich GD, Hu FZ, Sotereanos N, Sewicke J, Parvizi J, Nara PL, et al. What role do periodontal pathogens play in osteoarthritis and periprosthetic joint infections of the knee? J Appl Biomater Funct Mater. 2014;12(1):13-20.

47. Eiamprapai P, Matsumura Y, Hiraumi H, Yamamoto N, Takakura S, Ito J. Rapid detection of bacterial DNA in mastoid granulation tissue with nested-PCR technique. J Med Assoc Thai. 2013;96(4):460-6.

48. Emberger M, Koller J, Laimer M, Hell M, Oender K, Trost A, et al. Nosocomial staphylococcal scalded skin syndrome caused by intra-articular injection. J Eur Acad Dermatol Venereol. 2011;25(2):227-31.

49. Eslami A, Can NT, Ng DL. Infectious disease diagnosed by fine needle aspiration biopsy. J Am Soc Cytopathology. 2020;9(3):152-8.

50. Fang XY, Li WB, Zhang CF, Huang ZD, Zeng HY, Dong Z, et al. Detecting the Presence of Bacterial DNA and RNA by Polymerase Chain Reaction to Diagnose Suspected Periprosthetic Joint Infection after Antibiotic Therapy. Orthop Surg. 2018;10(1):40-6.

51. Ferroni A, Al Khoury H, Dana C, Quesne G, Berche P, Glorion C, et al. Prospective survey of acute osteoarticular infections in a French paediatric orthopedic surgery unit. Clinical Microbiology and Infection. 2013;19(9):822-8.

52. Fihman V, Hannouche D, Bousson V, Bardin T, Lioté F, Raskine L, et al. Improved diagnosis specificity in bone and joint infections using molecular techniques. Journal of Infection. 2007;55(6):510-7.

53. Fuursted K, Arpi M, Lindblad BE, Pedersen LN. Broad-range PCR as a supplement to culture for detection of bacterial pathogens in patients with a clinically diagnosed spinal infection. Scandinavian Journal of Infectious Diseases. 2008;40(10):772-7.

54. Gan C, Hu JF, Cao Q, Zhao RK, Li YC, Wang ZG, et al. Rapid identification of pathogens involved in pediatric osteoarticular infections by multiplex PCR. Annals of Translational Medicine. 2020;8(5).

55. Gasbarrini A, Boriani L, Salvadori C, Mobarec S, Kreshak J, Nanni C, et al. Biopsy for suspected spondylodiscitis. Eur Rev Med Pharmacol Sci. 2012;16(SUPPL. 2):26-34.

56. Gomes RT, Lyra TG, Alves NN, Caldas RM, Barberino MG, Nascimento-Carvalho CM. Methicillin-resistant and methicillin-susceptible community-acquired Staphylococcus aureus infection among children. Brazilian Journal of Infectious Diseases. 2013;17(5):573-8.

57. Gomez E, Cazanave C, Cunningham SA, Greenwood-Quaintance KE, Steckelberg JM, Uhl JR, et al. Prosthetic joint infection diagnosis using broad-range PCR of biofilms dislodged from knee and hip arthroplasty surfaces using sonication. Journal of Clinical Microbiology. 2012;50(11):3501-8.

58. Gonzalez BE, Martinez-Aguilar G, Hulten KG, Hammerman WA, Coss-Bu J, Avalos-Mishaan A, et al. Severe staphylococcal sepsis in adolescents in the era of community-acquired methicillin-resistant Staphylococcus aureus. Pediatrics. 2005;115(3):642-8.

59. Grif K, Heller I, Prodinger WM, Lechleitner K, Lass-Flörl C, Orth D. Improvement of detection of bacterial pathogens in normally sterile body sites with a focus on orthopedic samples by use of a commercial 16S rRNA broad-range PCR and sequence analysis. Journal of Clinical Microbiology. 2012;50(7):2250-4.

60. Gu FF, Han LZ, Chen X, Wang YC, Shen H, Wang JQ, et al. Molecular characterization of Staphylococcus aureus from surgical site infections in orthopedic patients in an orthopedic trauma clinical medical center in Shanghai. Surg Infect (Larchmt). 2015;16(1):97-104.

61. Gu W, Deng X, Lee M, Sucu YD, Arevalo S, Stryke D, et al. Rapid pathogen detection by metagenomic next-generation sequencing of infected body fluids. Nat Med. 2021;27(1):115-24.

62. Guarner J, Packard MM, Nolte KB, Paddock CD, Shieh WJ, Tondella ML, et al. Usefulness of immunohistochemical diagnosis of Streptococcus pneumoniae in formalin-fixed, paraffin-embedded specimens compared with culture and gram stain techniques. American Journal of Clinical Pathology. 2007;127(4):612-8.

63. Gubbay JB, Gosbell IB, Barbagiannakos T, Vickery AM, Mercer JL, Watson M. Clinical features, epidemiology, antimicrobial resistance, and exotoxin genes (including that of Panton-Valentine leukocidin) of gentamicin-susceptible methicillin-resistant Staphylococcus aureus (GS-MRSA) isolated at a paediatric teaching hospital in New South Wales, Australia. Pathology. 2008;40(1):64-71.

64. Gutierrez J, Guimaraes AO, Lewin-Koh N, Berhanu A, Xu M, Cao Y, et al. Sustained Circulating Bacterial Deoxyribonucleic Acid Is Associated With Complicated Staphylococcus aureus Bacteremia. Open Forum Infect Dis. 2019;6(4):ofz090.

65. Haag H, Locher F, Nolte O. Molecular diagnosis of microbial aetiologies using SepsiTest™ in the daily routine of a diagnostic laboratory. Diagnostic Microbiology and Infectious Disease. 2013;76(4):413-8.

66. Harro JM, Shirtliff ME, Arnold W, Kofonow JM, Dammling C, Achermann Y, et al. Development of a Novel and Rapid Antibody-Based Diagnostic for Chronic Staphylococcus aureus Infections Based on Biofilm Antigens. Journal of Clinical Microbiology. 2020;58(5).

67. Helal S, El Anany M, Ghaith D, Rabeea S. The Role of MDR-Acinetobacter baumannii in Orthopedic Surgical Site Infections. Surgical Infections. 2015;16(5):518-22.

68. Henares D, Brotons P, Buyse X, Latorre I, de Paz HD, Muñoz-Almagro C. Evaluation of the eazyplex MRSA assay for the rapid detection of Staphylococcus aureus in pleural and synovial fluid. International Journal of Infectious Diseases. 2017;59:65-8.

69. Hinić V, Aittakorpi A, Suter S, Turan S, Schultheiss E, Frei R, et al. Evaluation of the novel microarray-based Prove-it™ Bone&Joint assay for direct detection of pathogens from normally sterile body sites in comparison with culture and broad-range bacterial PCR. J Microbiol Methods. 2014;107:38-40.

70. Ilharreborde B, Bidet P, Lorrot M, Even J, Mariani-Kurkdjian P, Liguori S, et al. New real-time PCR-based method for Kingella kingae DNA detection: application to samples collected from 89 children with acute arthritis. J Clin Microbiol. 2009;47(6):1837-41.

71. Jacovides CL, Kreft R, Adeli B, Hozack B, Ehrlich GD, Parvizi J. Successful identification of pathogens by polymerase chain reaction (PCR)-based electron spray ionization time-of-flight mass spectrometry (ESI-TOF-MS) in culture-negative periprosthetic joint infection. J Bone Jt Surg Ser A. 2012;94(24):2247-54.

72. Jacquier H, Fihman V, Amarsy R, Vicaut E, Bousson V, Cambau E, et al. Benefits of polymerase chain reaction combined with culture for the diagnosis of bone and joint infections: A prospective test performance study. Open Forum Infectious Diseases. 2019;6(12).

73. Jahoda D, Landor I, Benedík J, Pokorný D, Judl T, Barták V, et al. PCR diagnostic system in the treatment of prosthetic joint infections. Folia Microbiol (Praha). 2015;60(5):385-91.

74. Jain S, Chowdhury R, Datta M, Chowdhury G, Mukhopadhyay AK. Characterization of the clonal profile of methicillin resistant Staphylococcus aureus isolated from patients with early post-operative orthopedic implant based infections. Ann Clin Microbiol Antimicrob. 2019;18(1):8.

75. Jeon HS, Hong SP, Cho BO, Mulyukin A, Choi JY, Kim SG. Hematogenous infection of the human temporomandibular joint. Oral Surg Oral Med Oral Pathol Oral Radiol Endod. 2005;99(2):E11-7.

76. Jiang B, Wang Y, Feng Z, Xu L, Tan L, Zhao S, et al. Panton-Valentine leucocidin (PVL) as a potential indicator for prevalence, duration, and severity of Staphylococcus aureus osteomyelitis. Front Microbiol. 2017;8(NOV).

77. Juchler C, Spyropoulou V, Wagner N, Merlini L, Dhouib A, Manzano S, et al. The Contemporary Bacteriologic Epidemiology of Osteoarticular Infections in Children in Switzerland. Journal of Pediatrics. 2018;194:190-6.e1.

78. Kalogianni DP, Goura S, Aletras AJ, Christopoulos TK, Chanos MG, Christofidou M, et al. Dry reagent dipstick test combined with 23S rRNA PCR for molecular diagnosis of bacterial infection in arthroplasty. Anal Biochem. 2007;361(2):169-75.

79. Karbysheva S, Yermak K, Grigoricheva L, Renz N, Perka C, Trampuz A. Synovial Fluid D-Lactate-A Novel Pathogen-Specific Biomarker for the Diagnosis of Periprosthetic Joint Infection. Journal of Arthroplasty. 2020;35(8):2223-+.

80. Kathju S, Lasken RS, Satish L, Johnson S, Stoodley P, Post JC, et al. Multiple displacement amplification as an adjunct to PCR-based detection of Staphylococcus aureus in synovial fluid. BMC Research Notes. 2010;3.

81. Katsarou I, Paraskevopoulou NM, Papadimitriou-Olivgeris M, Giormezis N, Militsopoulou M, Kolonitsiou F, et al. Fatality of Staphylococcus aureus infections in a Greek university hospital: role of inappropriate empiric treatment, methicillin resistance, and toxin genes' presence. Eur J Clin Microbiol Infect Dis. 2020;39(3):443-50.

82. Kawamura M, Kobayashi N, Inaba Y, Choe H, Tezuka T, Kubota S, et al. A new multiplex real-time polymerase chain reaction assay for the diagnosis of periprosthetic joint infection. Mod Rheumatol. 2017;27(6):1072-8.

83. Kechrid A, Pérez-Vázquez M, Smaoui H, Hariga D, Rodríguez-Baños M, Vindel A, et al. Molecular analysis of community-acquired methicillin-susceptible and resistant Staphylococcus aureus isolates recovered from bacteraemic and osteomyelitis infections in children from Tunisia. Clin Microbiol Infect. 2011;17(7):1020-6.

84. Khosravi AD, Hoveizavi H, Farshadzadeh Z. The prevalence of genes encoding leukocidins in Staphylococcus aureus strains resistant and sensitive to methicillin isolated from burn patients in Taleghani hospital, Ahvaz, Iran. Burns. 2012;38(2):247-51.

85. Kim DH, Spencer M, Davidson SM, Li L, Shaw JD, Gulczynski D, et al. Institutional prescreening for detection and eradication of methicillin-resistant Staphylococcus aureus in patients undergoing elective orthopaedic surgery. J Bone Jt Surg Ser A. 2010;92(9):1820-6.

86. Kim H, Kim J, Ihm C. The usefulness of multiplex PCR for the identification of bacteria in joint infection. Journal of Clinical Laboratory Analysis. 2010;24(3):175-81.

87. Kobayashi N, Inaba Y, Choe H, Aoki C, Ike H, Ishida T, et al. Simultaneous Intraoperative Detection of Methicillin-Resistant Staphylococcus and Pan-Bacterial Infection During Revision Surgery Use of Simple DNA Release by Ultrasonication and Real-Time Polymerase Chain Reaction. Journal of Bone and Joint Surgery-American Volume. 2009;91A(12):2896-902.

88. Kobayashi N, Inaba Y, Choe H, Iwamoto N, Ishida T, Yukizawa Y, et al. Rapid and sensitive detection of methicillin-resistant Staphylococcus periprosthetic infections using real-time polymerase chain reaction. Diagn Microbiol Infect Dis. 2009;64(2):172-6.

89. Kobayashi N, Procop GW, Krebs V, Kobayashi H, Bauer TW. Molecular identification of bacteria from aseptically loose implants. Clin Orthop Relat Res. 2008;466(7):1716-25.

90. Kourbatova EV, Halvosa JS, King MD, Ray SM, White N, Blumberg HM. Emergence of community-associated methicillin-resistant Staphylococcus aureus USA 300 clone as a cause of health care-associated infections among patients with prosthetic joint infections. Am J Infect Control. 2005;33(7):385-91.

91. Lallemand E, Coiffier G, Arvieux C, Brillet E, Guggenbuhl P, Jolivet-Gougeon A. MALDI-TOF MS performance compared to direct examination, culture, and 16S rDNA PCR for the rapid diagnosis of bone and joint infections. European Journal of Clinical Microbiology and Infectious Diseases. 2016;35(5):857-66.

92. Lane MA, Ganeshraj N, Gu A, Warren DK, Burnham CD. Lack of Additional Diagnostic Yield of 16s rRNA Gene PCR for Prosthetic Joint Infections. J Appl Lab Med. 2019;4(2):224-8.

93. Levy PY, Fournier PE, Fenollar F, Raoult D. Systematic PCR Detection in Culture-negative Osteoarticular Infections. American Journal of Medicine. 2013;126(12).

94. Ley BE, Linton CJ, Bennett DM, Jalal H, Foot AB, Millar MR. Detection of bacteraemia in patients with fever and neutropenia using 16S rRNA gene amplification by polymerase chain reaction. Eur J Clin Microbiol Infect Dis. 1998;17(4):247-53.

95. Lourtet-Hascoëtt J, Bicart-See A, Félicé MP, Giordano G, Bonnet E. Is Xpert MRSA/SA SSTI real-time PCR a reliable tool for fast detection of methicillin-resistant coagulase-negative staphylococci in periprosthetic joint infections? Diagnostic Microbiology and Infectious Disease. 2015;83(1):59-62.

96. Mariani BD, Levine MJ, Booth RE, Tuan RS. Development of a novel, rapid processing protocol for polymerase chain reaction-based detection of bacterial infections in synovial fluids. Molecular Biotechnology. 1995;4(3):227-37.

97. Mariani BD, Martin DS, Chen AF, Yagi H, Lin SS, Tuan RS. Polymerase Chain Reaction molecular diagnostic technology for monitoring chronic osteomyelitis. J Exp Orthop. 2014;1(1):1-10.

98. Mariaux S, Tafin UF, Borens O. Diagnosis Of Persistent Infection In Prosthetic Two-Stage Exchange: PCR analysis of Sonication fluid From Bone Cement Spacers. J Bone Jt Infect. 2017;2(4):218-23.

99. McCaskill ML, Mason Jr EO, Kaplan SL, Hammerman W, Lamberth LB, Hultén KG. Increase of the USA300 clone among community-acquired methicillin- susceptible Staphylococcus aureus causing invasive infections. Pediatric Infectious Disease Journal. 2007;26(12):1122-7.

100. Melendez DP, Greenwood-Quaintance KE, Berbari EF, Osmon DR, Mandrekar JN, Hanssen AD, et al. Evaluation of a Genus- and Group-Specific Rapid PCR Assay Panel on Synovial Fluid for Diagnosis of Prosthetic Knee Infection. Journal of Clinical Microbiology. 2016;54(1):120-6.

101. Metso L, Mäki M, Tissari P, Remes V, Piiparinen P, Kirveskari J, et al. Efficacy of a novel PCR-and microarray-based method in diagnosis of a prosthetic joint infection. Acta Orthop. 2014;85(2):165-70.

102. Minegishi Y, Uchiyama K, Sakurai K, Ibe S, Kanda H, Nihonyanagi S, et al. Clinical usefulness of multiplex PCR-lateral flow for the diagnosis of orthopedic-related infections. Mod Rheumatol. 2019;29(5):867-73.

103. Miyamae Y, Inaba Y, Kobayashi N, Choe H, Ike H, Momose T, et al. Quantitative evaluation of periprosthetic infection by real-time polymerase chain reaction: A comparison with conventional methods. Diagnostic Microbiology and Infectious Disease. 2012;74(2):125-30.

104. Moojen DJF, Spijkers SNM, Schot CS, Nijhof MW, Vogely HC, Fleer A, et al. Identification of orthopaedic infections using broad-range polymerase chain reaction and reverse line blot hybridization. J Bone Jt Surg Ser A. 2007;89(6):1298-305.

105. Morel AS, Dubourg G, Prudent E, Edouard S, Gouriet F, Casalta JP, et al. Complementarity between targeted real-time specific PCR and conventional broad-range 16S rDNA PCR in the syndrome-driven diagnosis of infectious diseases. European Journal of Clinical Microbiology & Infectious Diseases. 2015;34(3):561-70.

106. Morgenstern C, Cabric S, Perka C, Trampuz A, Renz N. Synovial fluid multiplex PCR is superior to culture for detection of low-virulent pathogens causing periprosthetic joint infection. Diagnostic Microbiology and Infectious Disease. 2018;90(2):115-9.

107. Morgenstern C, Renz N, Cabric S, Perka C, Trampuz A. Multiplex polymerase chain reaction and microcalorimetry in synovial fluid: Can pathogen-based detection assays improve the diagnosis of septic arthritis? J Rheumatol. 2018;45(11):1588-93.

108. Muñoz-Gamito G, Cuchí E, Roigé J, Gómez L, Jaén À, Matamala A, et al. Higher accuracy of genotypic identification compared to phenotyping in the diagnosis of coagulase-negative staphylococcus infection in orthopedic surgery. Infect Dis (Lond). 2020;52(12):883-90.

109. Mussa M, Manciulli T, Corbella M, Mariani B, Cambieri P, Gipsz N, et al. Epidemiology and microbiology of prosthetic joint infections: a nine-year, single-center experience in Pavia, Northern Italy. Musculoskelet Surg. 2021;105(2):195-200.

110. Ong MHL, Ho WY, Ng WW, Chew CH. High prevalence of tetM as compared to tetK amongst methicillin-resistant Staphylococcus aureus (MRSA) isolates from hospitals in Perak, Malaysia. Jundishapur Journal of Microbiology. 2017;10(6).

111. Palmer MP, Melton-Kreft R, Nistico L, Louisa Hiller N, Kim LHJ, Altman GT, et al. Polymerase Chain Reaction-Electrospray-Time-of-Flight Mass Spectrometry Versus Culture for Bacterial Detection in Septic Arthritis and Osteoarthritis. Gent Test and Mol Biomarkers. 2016;20(12):721-31.

112. Papan C, Meyer-Buehn M, Laniado G, Huebner J. Evaluation of the multiplex PCR based assay Unyvero implant and tissue infection application for pathogen and antibiotic resistance gene detection in children and neonates. Infection. 2019;47(2):195-200.

113. Parcell BJ, Phillips G. Use of Xpert® MRSA PCR point-of-care testing beyond the laboratory. Journal of Hospital Infection. 2014;87(2):119-21.

114. Pardo L, Vola M, Macedo-Viñas M, Machado V, Cuello D, Mollerach M, et al. Community-associated methicillin-resistant Staphylococcus aureus in children treated in Uruguay. J Infect Dev Ctries. 2013;7(1):10-6.

115. Portillo ME, Salvadó M, Sorli L, Alier A, Martínez S, Trampuz A, et al. Multiplex PCR of sonication fluid accurately differentiates between prosthetic joint infection and aseptic failure. Journal of Infection. 2012;65(6):541-8.

116. Post V, Wahl P, Uçkay I, Ochsner P, Zimmerli W, Corvec S, et al. Phenotypic and genotypic characterisation of Staphylococcus aureus causing musculoskeletal infections. Int J Med Microbiol. 2014;304(5-6):565-76.

117. Premru MM, Spik VC, Furlan SL, Zupanc TL. Clinical appearance of Staphylococcus aureus spondylodiscitis and molecular characterization of the isolates. Scand J Infect Dis. 2010;42(10):763-6.

118. Rahman MM, Amin KB, Rahman SMM, Khair A, Rahman M, Hossain A, et al. Investigation of methicillin-resistant Staphylococcus aureus among clinical isolates from humans and animals by culture methods and multiplex PCR. BMC Vet Res. 2018;14(1):300.

119. Reissier S, Lazareth I, Adjiman L, Couzigou C, Vidal B, Mizrahi A, et al. Evaluation of the Unyvero i60 ITI (R) multiplex PCR for infected chronic leg ulcers diagnosis. J Microbiol Methods. 2020;168.

120. Renz N, Cabric S, Morgenstern C, Schuetz MA, Trampuz A. Value of PCR in sonication fluid for the diagnosis of orthopedic hardware-associated infections: Has the molecular era arrived? Injury. 2018;49(4):806-11.

121. Renz N, Feihl S, Cabric S, Trampuz A. Performance of automated multiplex PCR using sonication fluid for diagnosis of periprosthetic joint infection: a prospective cohort. Infection. 2017;45(6):877-84.

122. Rincón S, Reyes J, Carvajal LP, Rojas N, Cortés F, Panesso D, et al. Cefazolin high-inoculum effect in methicillin-susceptible Staphylococcus aureus from South American hospitals. J Antimicrob Chemother. 2013;68(12):2773-8.

123. Rosey AL, Abachin E, Quesnes G, Cadilhac C, Pejin Z, Glorion C, et al. Development of a broad-range 16S rDNA real-time PCR for the diagnosis of septic arthritis in children. J Microbiol Methods. 2007;68(1):88-93.

124. Rupp M, Kern S, Weber T, Menges TD, Schnettler R, Heiß C, et al. Polymicrobial infections and microbial patterns in infected nonunions - a descriptive analysis of 42 cases. BMC Infect Dis. 2020;20(1):667.

125. Russell CD, Ramaesh R, Kalima P, Murray A, Gaston MS. Microbiological characteristics of acute osteoarticular infections in children. J Med Microbiol. 2015;64(Pt 4):446-53.

126. Ryu SY, Greenwood-Quaintance KE, Hanssen AD, Mandrekar JN, Patel R. Low sensitivity of periprosthetic tissue PCR for prosthetic knee infection diagnosis. Diagnostic Microbiology and Infectious Disease. 2014;79(4):448-53.

127. Saeed K, Ahmad N, Pallett A, Guiver M, Marsh P. Specific staphylococcal polymerase chain reaction can be a complementary tool for identifying causative organisms and guiding antibiotic management in orthopaedic infections. Curr Orthop Pract. 2010;21(6):628-31.

128. Sambri A, Pignatti G, Romagnoli M, Donati D, Marcacci M, Cadossi M. Intraoperative diagnosis of Staphylococcus aureus and coagulase-negative Staphylococcus using Xpert MRSA/SA SSTI assay in prosthetic joint infection. New Microbiol. 2017;40(2):130-4.

129. Sampedro MF, Huddleston PM, Piper KE, Karau MJ, Dekutoski MB, Yaszemski MJ, et al. A biofilm approach to detect bacteria on removed spinal implants. Spine. 2010;35(12):1218-24.

130. San Juan R, Chaves F, López Gude MJ, Díaz-Pedroche C, Otero J, Cortina Romero JM, et al. Staphylococcus aureus poststernotomy mediastinitis: description of two distinct acquisition pathways with different potential preventive approaches. J Thorac Cardiovasc Surg. 2007;134(3):670-6.

131. Sancho-Tello S, Bravo D, Borrás R, Costa E, Muñoz-Cobo B, Navarro D. Performance of the lightCycler septiFast test M grade in detecting microbial pathogens in purulent fluids. Journal of Clinical Microbiology. 2011;49(8):2988-91.

132. Sarma JB, Ahmed GU. Characterisation of methicillin resistant S. aureus strains and risk factors for acquisition in a teaching hospital in northeast India. Indian J Med Microbiol. 2010;28(2):127-9.

133. Sauer P, Gallo J, Kesselová M, Kolár M, Koukalová D. Universal primers for detection of common bacterial pathogens causing prosthetic joint infection. Biomed Pap Med Fac Univ Palacky Olomouc Czech Repub. 2005;149(2):285-8.

134. Saviauk T, Kiiski JP, Nieminen MK, Tamminen NN, Roine AN, Kumpulainen PS, et al. Electronic Nose in the Detection of Wound Infection Bacteria from Bacterial Cultures: A Proof-of-Principle Study. Eur Surg Res. 2018;59(1-2):1-11.

135. Sdougkos G, Chini V, Papanastasiou DA, Christodoulou G, Stamatakis E, Vris A, et al. Community-associated Staphylococcus aureus infections and nasal carriage among children: molecular microbial data and clinical characteristics. Clin Microbiol Infect. 2008;14(11):995-1001.

136. Searns JB, Robinson CC, Wei Q, Yuan J, Hamilton S, Pretty K, et al. Validation of a novel molecular diagnostic panel for pediatric musculoskeletal infections: Integration of the Cepheid Xpert MRSA/SA SSTI and laboratory-developed real-time PCR assays for clindamycin resistance genes and Kingella kingae detection. J Microbiol Methods. 2019;156:60-7.

137. Sebastian S, Malhotra R, Sreenivas V, Kapil A, Chaudhry R, Dhawan B. A Clinico-Microbiological Study of Prosthetic Joint Infections in an Indian Tertiary Care Hospital: Role of Universal 16S rRNA Gene Polymerase Chain Reaction and Sequencing in Diagnosis. Indian J Orthop. 2019;53(5):646-54.

138. Shetty DJ, Mulki SS, Kamath BNJ, Bhat S, Bhat A. Identification of cultivable and non-cultivable organisms causing intraarticular and bone infections using molecular diagnostic techniques. European Journal of Molecular and Clinical Medicine. 2021;8(3):643-51.

139. Sigmund IK, Holinka J, Sevelda F, Staats K, Heisinger S, Kubista B, et al. Performance of automated multiplex polymerase chain reaction (mPCR) using synovial fluid in the diagnosis of native joint septic arthritis in adults. Bone Joint J. 2019;101-b(3):288-96.

140. Sigmund IK, Renz N, Feihl S, Morgenstern C, Cabric S, Trampuz A. Value of multiplex PCR for detection of antimicrobial resistance in samples retrieved from patients with orthopaedic infections. BMC Microbiol. 2020;20(1):88.

141. Sigmund IK, Windhager R, Sevelda F, Staats K, Puchner SE, Stenicka S, et al. Multiplex PCR Unyvero i60 ITI application improves detection of low-virulent microorganisms in periprosthetic joint infections. Int Orthop. 2019;43(8):1891-8.

142. Stoodley P, Conti SF, DeMeo PJ, Nistico L, Melton-Kreft R, Johnson S, et al. Characterization of a mixed MRSA/MRSE biofilm in an explanted total ankle arthroplasty. FEMS Immunol Med Microbiol. 2011;62(1):66-74.

143. Stuhlmeier R, Stuhlmeier KM. Fast, simultaneous, and sensitive detection of staphylococci. J Clin Pathol. 2003;56(10):782-5.

144. Subhadharsini S, Gopinath P. Detection of the presence of Bbp gene for bone bound sialoprotein among clinical isolates of Staphylococcus aureus. Res J Pharm Technol. 2016;9(9):1430-2.

145. Suren C, Feihl S, Cabric S, Banke IJ, Haller B, Trampuz A, et al. Improved pre-operative diagnostic accuracy for low-grade prosthetic joint infections using second-generation multiplex Polymerase chain reaction on joint fluid aspirate. International Orthopaedics. 2020;44(9):1629-37.

146. Szczȩsny G, Interewicz B, Swoboda-Kopeć E, Olszewski WL, Górecki A, Wasilewski P. Bacteriology of Callus of Closed Fractures of Tibia and Femur. Journal of Trauma - Injury, Infection and Critical Care. 2008;65(4):837-42.

147. Tarkin IS, Henry TJ, Fey PI, Iwen PC, Hinrichs SH, Garvin KL. PCR rapidly detects methicillin-resistant staphylococci periprosthetic infection. Clin Orthop Relat Res. 2003(414):89-94.

148. Tenover FC, Eloi K, Tickler IA, Cohen S, Schneider GB, Vlad SC. Strain types of Staphylococcus aureus nasal isolates from persons undergoing joint replacement surgery. Journal of Hospital Infection. 2018;98(2):168-70.

149. Titécat M, Loïez C, Senneville E, Wallet F, Dezèque H, Legout L, et al. Evaluation of rapid mecA gene detection versus standard culture in staphylococcal chronic prosthetic joint infections. Diagnostic Microbiology and Infectious Disease. 2012;73(4):318-21.

150. Titécat M, Wallet F, Robineau O, Valette M, Migaud H, Senneville E, et al. Focus on MRSA/SA SSTI assay failure in prosthetic joint infections: 213 consecutive patients later. Journal of Clinical Microbiology. 2017;55(2):635-7.

151. Tsai YH, Chen PH, Yu PA, Chen CL, Kuo LT, Huang KC. A multiplex PCR assay for detection of Vibrio vulnificus, Aeromonas hydrophila, methicillin-resistant Staphylococcus aureus, Streptococcus pyogenes, and Streptococcus agalactiae from the isolates of patients with necrotizing fasciitis. Int J Infect Dis. 2019;81:73-80.

152. Tsuru A, Setoguchi T, Kawabata N, Hirotsu M, Yamamoto T, Nagano S, et al. Enrichment of bacteria samples by centrifugation improves the diagnosis of orthopaedics-related infections via real-time PCR amplification of the bacterial methicillin-resistance gene. BMC Research Notes. 2015;8(1).

153. Uçkay I, Teterycz D, Ferry T, Harbarth S, Lübbeke A, Emonet S, et al. Poor utility of MRSA screening to predict staphylococcal species in orthopaedic implant infections. Journal of Hospital Infection. 2009;73(1):89-91.

154. Valour F, Blanc-Pattin V, Freydière AM, Bouaziz A, Chanard E, Lustig S, et al. Rapid detection of Staphylococcus aureus and methicillin resistance in bone and joint infection samples: Evaluation of the GeneXpert MRSA/SA SSTI assay. Diagnostic Microbiology and Infectious Disease. 2014;78(3):313-5.

155. Vandercam B, Jeumont S, Cornu O, Yombi JC, Lecouvet F, Lefèvre P, et al. Amplification-based DNA analysis in the diagnosis of prosthetic joint infection. J Mol Diagn. 2008;10(6):537-43.

156. Vasoo S, Cunningham SA, Greenwood-Quaintance KE, Mandrekar JN, Hanssen AD, Abdel MP, et al. Evaluation of the FilmArray blood culture ID panel on biofilms dislodged from explanted arthroplasties for prosthetic joint infection diagnosis. Journal of Clinical Microbiology. 2015;53(8):2790-2.

157. Villa F, Toscano M, De Vecchi E, Bortolin M, Drago L. Reliability of a multiplex PCR system for diagnosis of early and late prosthetic joint infections before and after broth enrichment. Int J Med Microbiol. 2017;307(6):363-70.

158. Wang CX, Huang Z, Fang X, Li W, Yang B, Zhang W. Comparison of broad-range polymerase chain reaction and metagenomic next-generation sequencing for the diagnosis of prosthetic joint infection. International Journal of Infectious Diseases. 2020;95:8-12.

159. Wang DG. Novel primers for increased specificity and sensitivity for the detection of Staphylococcus aureus by real-time LAMP. Cyta-Journal of Food. 2016;14(1):88-91.

160. Wang DG, Wang YZ, Xiao FG, Guo WY, Zhang YQ, Wang AP, et al. A Comparison of In-House Real-Time LAMP Assays with a Commercial Assay for the Detection of Pathogenic Bacteria. Molecules. 2015;20(6):9487-95.

161. Wang FD, Wu PF, Chen SJ. Distribution of virulence genes in bacteremic methicillin-resistant Staphylococcus aureus isolates from various sources. J Microbiol Immunol Infect. 2019;52(3):426-32.

162. Wang LJ, Dong F, Qian SY, Yao KH, Song WQ. Clinical and Molecular Epidemiology of Invasive Staphylococcus aureus Infections in Chinese Children: A Single-center Experience. Chinese Medical Journal. 2017;130(23):2889-90.

163. Wang WY, Lee SY, Chiueh TS, Lu JJ. Molecular and phenotypic characteristics of methicillin-resistant and vancomycin-intermediate staphylococcus aureus isolates from patients with septic arthritis. J Clin Microbiol. 2009;47(11):3617-23.

164. Yang B, Fang X, Cai Y, Yu Z, Li W, Zhang C, et al. Detecting the presence of bacterial RNA by polymerase chain reaction in low volumes of preoperatively aspirated synovial fluid from prosthetic joint infections. Bone Jt Res. 2020;9(5):219-24.

165. Yang F, Choe H, Kobayashi N, Tezuka T, Oba M, Miyamae Y, et al. An automated real-time PCR assay for synovial fluid improves the preoperative etiological diagnosis of periprosthetic joint infection and septic arthritis. J Orthop Res. 2021;39(2):348-55.

166. Yang S, Ramachandran P, Hardick A, Hsieh YH, Quianzon C, Kuroki M, et al. Rapid PCR-based diagnosis of septic arthritis by early gram-type classification and pathogen identification. Journal of Clinical Microbiology. 2008;46(4):1386-90.

167. Zegaer BH, Ioannidis A, Babis GC, Ioannidou V, Kossyvakis A, Bersimis S, et al. Detection of Bacteria Bearing Resistant Biofilm Forms, by Using the Universal and Specific PCR is Still Unhelpful in the Diagnosis of Periprosthetic Joint Infections. Front Med (Lausanne). 2014;1:30.

168. Zhao M, Tang K, Liu F, Zhou W, Fan J, Yan G, et al. Metagenomic Next-Generation Sequencing Improves Diagnosis of Osteoarticular Infections From Abscess Specimens: A Multicenter Retrospective Study. Front Microbiol. 2020;11.

169. Zhu LX, Shen DX, Zhou QM, Li ZX, Fang XD, Li QZ. A Locked Nucleic Acid (LNA)-Based Real-Time PCR Assay for the Rapid Detection of Multiple Bacterial Antibiotic Resistance Genes Directly from Positive Blood Culture. Plos One. 2015;10(3).

170. Zhu LX, Shen DX, Zhou QM, Liu CJ, Li ZX, Fang XD, et al. Universal ProbeLibrary based real-time PCR for rapid detection of bacterial pathogens from positive blood culture bottles. World Journal of Microbiology & Biotechnology. 2014;30(3):967-75.
